# Comparing Symmetry-Adapted Perturbation Theory to Intermolecular Energy Decomposition Analysis Based on Absolutely Localized Molecular Orbitals

**DOI:** 10.1021/acs.jctc.6c01446

**Published:** 2026-10-10

**Authors:** Narayanan Puthillathu, Montgomery Gray, John M. Herbert

**Affiliations:** † Department of Chemistry & Biochemistry, 2647 The Ohio State University, Columbus, Ohio 43210, United States; ‡ Biophysics Graduate Program, The Ohio State University, Columbus, Ohio 43210, United States

## Abstract

Energy decomposition analysis (EDA) can provide insight into noncovalent interactions, even though the individual energy components (electrostatics, Pauli repulsion, polarization, dispersion, etc.) are not uniquely defined within quantum mechanics. The present work examines the two titular EDA schemes and elucidates the degree of transferability between their components. We first demonstrate that a many-body dispersion (MBD) model can replace the conventional definition of dispersion in symmetry-adapted perturbation theory (SAPT), preserving reasonable accuracy as compared to third-order SAPT but reducing the computational cost to be comparable to density functional theory (DFT). Individual SAPT + MBD energy components exhibit good correlation with those computed using the absolutely localized molecular orbital (ALMO) EDA scheme, which is built around DFT, such that qualitative explanations and chemical trends are likely to be similar in either approach. SAPT + MBD and ALMO-EDA electrostatic energies are nearly identical, provided that a SAPT-style direct-product reference state is used for ALMO-EDA, but other energy components exhibit substantial quantitative differences. These conclusions hold across a range of density functionals used in the ALMO-EDA calculations, and for different choices of the “dispersion-free” functional that is used to isolate the dispersion energy from the target functional’s interaction energy. The present work provides calibration plots to translate between SAPT + MBD and ALMO-EDA energy components. For a large data set of dimers representing ligand–protein interactions, SAPT + MBD calculations afford superior agreement with the best *ab initio* benchmarks, as compared to calculations using the state-of-the-art ωB97M-V functional, especially when triple-ζ basis sets are used for SAPT. This conclusion holds even when three-body (Axilrod–Teller–Muto) dispersion corrections are added to the ωB97M-V results.

## Graphical abstract

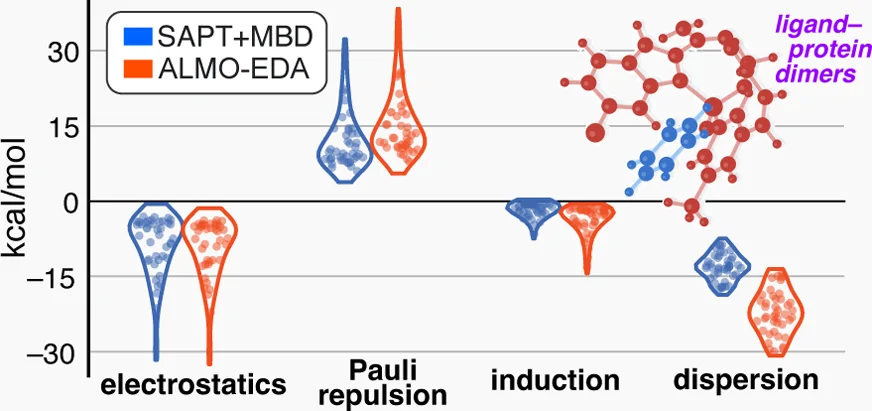

## 1 Introduction

Numerous schemes for *a posteriori* decomposition of noncovalent interaction energies into physically meaningful components have been developed for use with electronic structure calculations,^1–44^ some dating to the early days of quantum chemistry,^1,2^ and these have been extensively reviewed elsewhere.^42–54^ Definitions of energy components such as electrostatics, polarization, Pauli repulsion, and dispersion are not unique, however, and none corresponds directly to a physical observable. This has made energy decomposition analysis (EDA) into a contentious matter,^55–61^ at least among theorists, even as these methods remain widely used in applications. Two of the present authors have gone so far as to liken EDA to the question of “how many kJ/mol can dance on the head of a pin?”.^62^ Despite this inherent ambiguity, we nevertheless believe that calls to abandon the analysis of weak interactions using EDA, as some have suggested,^61^ are misguided and overly cynical. There is value in understanding chemical trends using EDA, even if numerical values of the energy components must be interpreted with caution and not taken as absolute arbiters of what is correct.

EDA for covalent bonds also has a long history,^63–74^ but the present work concerns only the decomposition of intermolecular interaction energies. Within that scope, an alternative approach to EDA is symmetry-adapted perturbation theory (SAPT).^75–83^ Whereas *a posteriori* EDA schemes start from a supramolecular calculation of the interaction energy for dimer A···B,

1 $$\Delta E_{\mathrm{int}}\left(A\cdot \cdot \cdot B\right)=E\left(A\cdot \cdot \cdot B\right)-E\left(A\right)-E\left(B\right)$$

SAPT builds up the interaction energy starting from wave functions for the isolated monomers A and B. Energy components are computed directly from perturbation theory, not by subtraction, and their sum affords the total interaction energy. Practitioners of SAPT have sometimes argued that it is better grounded in quantum mechanics as compared to other EDAs,^58^ although it is not obvious to us that this is true. Certainly, there is some arbitrariness in how the terms in perturbation theory are grouped into energy components and labeled.^78^ That said, individual terms in the perturbation expansion are independent of one another and this provides SAPT-based methods with a certain flexibility, according to which hybrid variants can be constructed that model various energy components in different ways.^37–44^ This is the basis of an “extended” (X)­SAPT approach that has been pursued by our group.^42–44^

Against this backdrop, the present work aims to elucidate similarities between energy components in two widely used EDA schemes. Recent comparisons of SAPT results to EDA methods based on density functional theory (DFT) have concluded that the partitions are roughly similar,^53,85,86^ with the largest deviations in the case of dispersion.^53^ This is consistent with our own analysis,^62^ in which we compared SAPT to DFT-based energy components obtained from a second-generation EDA based on absolutely localized molecular orbitals (ALMO-EDA2),^23–25^ the details of which are described in Section 2.3. The ALMO-EDA2 approach predicts dispersion energies that are systematically smaller than SAPT values (Figure 1). In the present work, this preliminary conclusion is examined for a wider range of density functionals and a broader array of noncovalent dimers.

**Figure 1:**
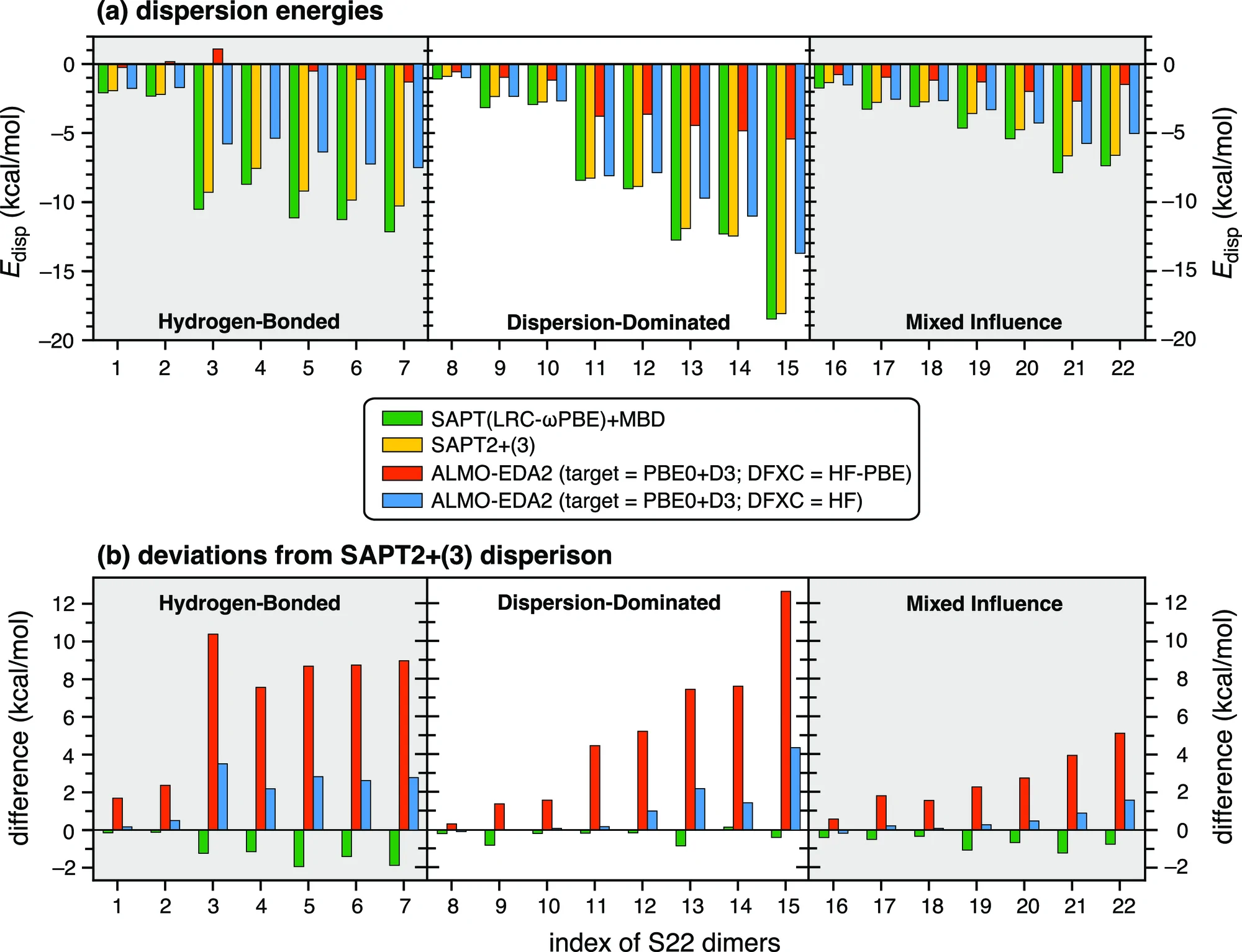
(a) Dispersion energies for the S22 data set computed using various methods. (b) Differences relative to SAPT2+(3) dispersion energies. The ALMO-EDA2 calculations use PBE0+D3­(BJ) as the target functional and HF exchange, either with or without PBE correlation, as the dispersion-free functional. Dimers are grouped into the three canonical subsets of S22. All DFT calculations use the def2-QZVPD basis set. The SAPT2+(3) calculations use aug-cc-pVTZ and are taken from ref 84.

The predicate for comparing SAPT- and DFT-based EDA schemes is an assumption that exchange repulsion and Pauli repulsion describe the same physics, and similarly that polarization, induction, and orbital interactions are understood to refer to the same general phenomena.^53,87^ The extent to which these correspondences hold, quantitatively, is not immediately clear because it has been suggested that DFT-based EDA methods may intermingle the correlation that contributes to dispersion with the semilocal exchange that is also responsible for Pauli repulsion.^62,88–90^ Furthermore, ALMO-EDA2 calculations require the user to select a “dispersion-free” density functional, by means of which the dispersion contribution is isolated from the total interaction energy. This provides an additional degree of freedom, according to which some correlation that is not dispersion by one definition may be labeled as dispersion in ALMO-EDA2 calculations.^62^ This is not easy to isolate, as indicated by the dramatically different results in Figure 1 when correlation is added to the dispersion-free functional in the ALMO-EDA2 calculations.

In the present work, we explore this issue more thoroughly via side-by-side comparison of SAPT and ALMO-EDA2 energy components. For the latter, we examine a variety of density functionals that offer good performance for noncovalent interactions in small-molecule dimers, along with two different choices for the dispersion-free functional. Whereas previous accounts of close correspondence between SAPT and ALMO-EDA2 energy components were based strictly on small dimer benchmarks, with correspondingly weak interaction energies, quantitative differences between EDA schemes might be expected to grow with the magnitude of the interaction energy. We elucidate these differences using a new benchmark data set of larger biomolecular fragments, representing protein–ligand interactions.^91^

## 2 Theory

This section describes the EDA schemes that will be compared: conventional SAPT (Section 2.1), a hybrid SAPT approach that is scalable to larger molecules (Section 2.2), and finally ALMO-EDA2 (Section 2.3).

### 2.1 Conventional SAPT

Starting from a direct product of Hartree–Fock (HF) determinants for the monomers, conventional SAPT involves a perturbative expansion for the intermolecular interaction energy (*E*_int_), partitioned into components that include electrostatics (*E*_elst_), exchange or Pauli repulsion (*E*_exch_), induction (*E*_ind_), and dispersion (*E*_disp_):

2 $$E_{\mathrm{int}}=E_{elst}+E_{exch}+E_{ind}+E_{disp}$$

We use *E*_int_ here, rather than Δ*E*_int_ (as in eq 1), to emphasize that the SAPT interaction energy is not computed by subtraction. Mathematical expressions for the components in eq 2 can be found elsewhere.^75,92,93^ These are improvable order-by-order in perturbation theory.

Most SAPT calculations employ the second-order variant known as SAPT0,^78^ which is zeroth-order in the Møller–Plesset fluctuation potentials (meaning that monomer electron correlation is neglected) and second-order in the intermolecular interaction. However, third-order terms are needed in order to achieve accuracy on par with methods that include triple excitations, such as coupled-cluster theory with single, double, and perturbative triple excitations [CCSD­(T)]. Of the commonly used variants of SAPT, the most accurate is the one known as SAPT2+(3),^78–81,94^ which includes a subset of third-order terms and can achieve sub-kcal/mol accuracy with respect to CCSD­(T).^78–80,94,95^ SAPT2+(3) dispersion energies are available for some small dimers,^84^ and we use these as benchmarks in the comparisons to follow.

Whereas the SAPT0 dispersion energy is

3 $$E_{disp}^{SAPT0}=E_{disp}^{\left(20\right)}+E_{exch‐disp}^{\left(20\right)}$$

with indices indicating second-order expressions in the intermolecular perturbation and zeroth-order in the fluctuation potentials, the dispersion energy for SAPT2+(3) is taken to be^78,84^

$$\begin{array}{c} E_{disp}^{SAPT2+\left(3\right)}=E_{disp}^{\left(20\right)}+E_{disp}^{\left(30\right)}+E_{disp}^{\left(21\right)}+E_{disp}^{\left(22\right)}+E_{exch‐disp}^{\left(20\right)}. \end{array}$$ 4

This definition affords good correlation with alternative EDAs that directly decompose CCSD­(T) interaction energies.^96^

### 2.2 SAPT­(KS) + MBD

Second-order dispersion is not a benchmark-quality method^79,97^ and third-order SAPT exhibits *O*(*N* ^7^) scaling.^81^ As an intermediate alternative, our group has pursued hybrid approaches that substitute affordable models for *E*_disp_, in conjunction with a Kohn–Sham (KS) description of the monomers.^42–44^ This affords a cubic-scaling method^43,98^ that is capable of benchmark-quality accuracy.^42–44^ The variant pursued here is SAPT­(KS) + MBD,^40–43^ which uses a version of the many-body dispersion (MBD) model developed by Tkatchenko and co-workers.^99,100^

#### 2.2.1 Energy Components

SAPT­(KS) + MBD uses second-order SAPT for all components in eq 2 except for dispersion:^43^

5a $$E_{elst}^{SAPT\left(KS\right)}=E_{elst}^{\left(10\right)}$$

5b $$E_{exch}^{SAPT\left(KS\right)}=E_{exch}^{\left(10\right)}$$

5c $$E_{ind}^{SAPT\left(KS\right)}=E_{ind}^{\left(20\right)}+E_{exch‐ind}^{\left(20\right)}$$

This is akin to SAPT0 except that the reference-state wave functions are KS determinants, computed with a tuned range-separated hybrid functional.^97^ The physical meaning of these energy components has been discussed elsewhere.^82,101^

The SAPT induction energy (eq 5c) contains both polarization and charge transfer.^39,43^ There are ways to disentangle these two contributions^39,102–105^ but we have not done so. An additional correction

$$\delta_{HF}=\Delta E_{\mathrm{int}}^{HF}-E_{elst}^{\left(10\right)}-E_{exch}^{\left(10\right)}-E_{ind,resp}^{\left(20\right)}-E_{exch‐ind,resp}^{\left(20\right)}$$ 6

is sometimes added to the SAPT induction energy.^78,81^ Here, a counterpoise-corrected, supramolecular HF interaction energy (Δ*E*_int_^HF^) is used to account for infinite-order induction effects, albeit without electron correlation. The δ_HF_ correction can be significant in some cases, especially for hydrogen bonds,^42,97^ but is not included here except where explicitly stipulated. (Its effect is considered in Section 4.3.3) Response corrections to the induction energies,^106^ obtained from solving coupled-perturbed equations and indicated by the “resp” subscripts in eq 6, are also sometimes included in the second-order SAPT induction energy. These are not considered in the present work, except insofar as the δ_HF_ correction is included.

The dispersion energy in SAPT­(KS) + MBD is described using the range-separated, self-consistent screening (rsSCS) variant of the MBD model,^99^ augmented with a screened dipole–quadrupole (*C*_8_) dispersion interaction that extends MBD@rsSCS to shorter intermolecular separations.^40^ Together, this is

7 $$E_{disp}^{SAPT+MBD}=E_{disp}^{MBD@rsSCS}-s_{8}\sum_{A>B}^{atoms}f_{8}\left(R_{AB}\right)\frac{C_{8}^{AB}}{R_{AB}^{8}}$$

where *E*_disp_^MBD@rsSCS^ is the model developed in ref 99. Damping parameters for the *C*_8_ term are taken from the implementation of SAPT + MBD in ref 41.

#### 2.2.2 Electrostatics in Detail

For later comparison to ALMO-EDA, we elaborate here on the definition of electrostatics. The first-order electrostatic energy in eq 5a is simply the Coulomb interaction between charge densities for the isolated monomers, assembled at the geometry of the complex.^43,101^ It can be decomposed into nuclear–nuclear, electron–electron, and electron–nuclear contributions,

8 $$E_{elst}=E_{elst}^{nn}+E_{elst}^{ee}+E_{elst}^{en}$$

Expressions for these terms, written in atomic units (where 4πε_0_ = 1), are^43,101^

9a $$E_{elst}^{nn}=\sum_{a\in A}\sum_{b\in B}\frac{Z_{a}Z_{b}}{R_{ab}}$$

9b $$E_{elst}^{ee}=\int \frac{\rho_{A}\left(r_{1}\right)\rho_{B}\left(r_{2}\right)}{∥r_{1}-r_{2}∥}dr_{1}dr_{2}$$

and

9c $$E_{elst}^{en}=-\sum_{a\in A}\int \frac{Z_{a}\rho_{B}\left(r\right)}{∥r-R_{a}∥}dr-\sum_{b\in B}\int \frac{Z_{b}\rho_{A}\left(r\right)}{∥r-R_{b}∥}dr.$$

Here, *Z*_*a*_ and *Z*_*b*_ are atomic numbers for nuclei located at **R**_*a*_ and **R**_*b*_. The quantities ρ_A_(r) and ρ_B_(r) are electron densities for isolated monomers A and B, and the signs in eq 9c reflect the fact that these densities are typically defined to be positive-definite even though electrons are negatively charged. A compact version of eqs 8 and 9 is

10 $$E_{elst}=\int \frac{\rho_{A}^{total}\left(r_{1}\right)\rho_{B}^{total}\left(r_{2}\right)}{∥r_{1}-r_{2}∥}dr_{1}dr_{2}$$

where

11 $$\rho_{M}^{total}\left(r\right)=-\rho_{M}\left(r\right)+\sum_{m\in M}Z_{m}\delta \left(r-R_{m}\right)$$

is the total charge density for monomer *M* (= A or B), including both nuclei (*Z*_*m*_) and electrons (ρ_*M*_).

Electron densities ρ_A_(r) and ρ_B_(r) derive from monomer wave functions computed in isolation, which is how the zeroth-order states are defined in SAPT. The isolated-monomer wave functions are not antisymmetric with respect to A ↔ B exchange and that effect is incorporated perturbatively within SAPT. The first-order exchange energy in eq 5b is the leading term that includes an antisymmetrizer, so it is appropriate to equate that term with Pauli repulsion. Higher-order terms in perturbation theory acquire exchange counterparts, as in the definition of the induction energy (eq 5c).

In the DFT-based EDA literature, the electrostatic formula in eq 10 is sometimes called the “quasiclassical” Coulomb energy.^71^ An alternative is to use an antisymmetrized (Heitler-London) reference state,^24^ which would correspond more closely to^107^

12 $$E_{elst+exch}=E_{elst}+E_{exch}$$

including both quasiclassical electrostatics and exchange (Pauli) repulsion. There are several disadvantages to the use of an antisymmetrized reference state, however. For example, use of a Heitler-London reference state in SAPT places terms that are formally zeroth-order in the intermolecular perturbation into the interaction energy.^108^ A more tangible objection is that an antisymmetrized reference state causes the interaction energy in Ne_2_ to be described as primarily electrostatic rather than dispersion-dominated, at the equilibrium separation of 3.1 Å.^24^ Finally, the direct-product reference state makes more sense from the standpoint of modeling intermolecular interactions based on monomer properties, because charge densities ρ_*M*_(r) can be approximated using classical multipoles^85^ while induction and dispersion can be understood in terms of monomer polarizabilities. In contrast, an antisymmetrized reference state entangles the monomer charge densities and makes it difficult to use EDA to understand intermolecular interactions in terms of monomer properties.

### 2.3 ALMO-EDA

This approach begins from a block-localization algorithm to separate the polarization contribution to Δ*E*_int_ in eq 1, by means of a locally projected “self-consistent field for molecular interactions” [SCF­(MI)] procedure.^109–115^ The result is a partition of Δ*E*_int_ into a polarization component (Δ*E*_pol_) and a remainder that is designated as the “frozen” energy (Δ*E*_frz_):

13 $$\Delta E_{\mathrm{int}}=\Delta E_{pol}+\Delta E_{frz}$$

The present work employs the second-generation version of this procedure (“ALMO-EDA2”),^23–25^ in which Δ*E*_frz_ is further decomposed into electrostatics, Pauli repulsion, and dispersion contributions:

14 $$\Delta E_{frz}=\Delta E_{elec}+\Delta E_{Pauli}+\Delta E_{disp}$$

In eqs 13 and 14, we adorn the energy components with a “Δ”, in contrast to the SAPT decomposition in eq 2. This serves to remind that the ALMO-EDA2 components stem from an interaction energy obtained by supramolecular energy difference (eq 1), whereas the SAPT components do not.

In the present work, supramolecular Δ*E*_int_ calculations used in ALMO-EDA2 are always counterpoise corrected. Otherwise, basis-set superposition error (BSSE) manifests in the CT term,^21,26^ where it would affect the comparison to SAPT induction energies.^39,43^ Previous literature also suggests that convergence to the complete basis-set (CBS) limit is crucial for making comparisons to SAPT calculations,^87^ because the counterpoise correction can modify energy components.^116–118^ Thus, many of the numerical comparisons to follow employ a quadruple-ζ basis set (see Section 3.2.2 for details), although we will also present results for biomolecular dimers using more realistic double- and triple-ζ basis sets.

The remainder of this section describes the energy components in eq 14. Separation of dispersion from the frozen energy is discussed in Section 2.3.1 followed by electrostatics in Section 2.3.2. Pauli repulsion is what remains: Δ*E*_Pauli_ = Δ*E*_int_ – Δ*E*_pol_ – Δ*E*_disp_ – Δ*E*_elec_.

#### 2.3.1 Dispersion

In the ALMO-EDA2 scheme, dispersion is isolated from Δ*E*_frz_ by selecting a “dispersion-free” exchange-correlation (DFXC) functional. This means a functional that does not describe dispersion interactions well, or perhaps not at all. In contrast, the “target” functional that is used to compute Δ*E*_int_ should be one that is appropriate for describing noncovalent interactions. Once a user selects these two functionals, the ALMO-EDA2 dispersion energy is defined as^24,25^

15 $$\Delta E_{disp}=\Delta E_{xc}^{target}-\Delta E_{xc}^{DFXC}$$

Terms on the right in eq 15 are defined as

16 $$\Delta E_{xc}^{f}\left(AB\right)=E_{xc}^{f}\left(AB\right)-E_{xc}^{f}\left(A\right)-E_{xc}^{f}\left(B\right)$$

for dimer A···B and functional *f* ∈ {target, DFXC}. If the target functional is a hybrid, it is suggested to use HF theory (exact exchange without correlation) as the dispersion-free functional.^25,52^ If the target functional is semilocal, then it is suggested to use revPBE^119^ for the DFXC functional.^25,52^

Use of HF as the DFXC functional means that all of the correlation in Δ*E*_xc_^target^ is assigned to dispersion,^62^ although there are still correlation effects on SCF densities that manifest in other energy components. The problematic separation of semilocal correlation from dispersion at van der Waals contact distances has been discussed elsewhere,^88–90^ and there is no obvious way to accomplish this separation in a rigorous manner. A rudimentary attempt to pair a correlation functional with HF exchange, using HF-PBE as the DFXC functional for PBE0+D3­(BJ) calculations, does not afford results that are straightforward to interpret.^62^ In fact, that choice moves the ALMO-EDA2 dispersion energies further away from SAPT2+(3) benchmarks, relative to the use of HF (only) as the DFXC functional (Figure 1b).

In comparing Figure 1 to a similar figure in previous work,^62^ note that the latter used antisymmetrized product wave functions to define Δ*E*_disp_, leading to different dispersion energies as compared to those reported in Figure 1. One conclusion of the present work will be that antisymmetrized products should not be used where comparison to SAPT energetics is the goal, for theoretical reasons that are discussed in Section 2.3.2 and numerical reasons that are documented in Section 4.

A more systematic strategy to isolate dispersion from the semilocal XC functional is to parametrize a “dispersionless” density functional,^120–122^ in which accurate dispersion energies have been subtracted from the training data prior to parameter fitting. The present work examines the dispersionless hybrid functional called dlDF^120^ as one possible choice for the DFXC functional in eq 15. The dlDF functional was parametrized using the same functional form as M05-2X,^123^ using training data from which SAPT­(DFT)^76^ dispersion energies had been subtracted *a priori*.^120^ While this represents an attempt to use “dispersionless” training data, in reality the excision is imperfect. One may take the present work as an exercise in examining the extent to which such a partition is useful.

The possibility of using dlDF as the DFXC functional in eq 15 has been raised several times in the discussion of ALMO-EDA2 energy components.^24,25,52^ It has been suggested that dlDF might be an appropriate choice if the target functional is semilocal,^52^ even though the parent M05-2X functional is a hybrid. To the best of our knowledge, however, dlDF has not been tested in this capacity, however. Such tests are reported here, for both hybrid and semilocal functionals.

#### 2.3.2 Electrostatics

It is tempting to identify Δ*E*_elec_ and Δ*E*_Pauli_ in eq 14 with SAPT electrostatics (*E*_elst_) and exchange repulsion (*E*_exch_), respectively, but these definitions are actually quite different because ALMO-EDA2 energy components are computed using an antisymmetrized reference state.^24^ This distorts the monomer densities relative to their isolated counterparts, and we denote these distorted densities as ${\mathrm{\rho ̃}}_{A}\left(r\right)$ and ${\mathrm{\rho ̃}}_{B}\left(r\right)$ . The definition of Δ*E*_elec_ parallels the SAPT definition of *E*_elst_ (eq 10) but uses the distorted densities,

17 $$\Delta E_{elec}=\int \frac{{\mathrm{\rho ̃}}_{A}^{total}\left(r_{1}\right){\mathrm{\rho ̃}}_{B}^{total}\left(r_{2}\right)}{∥r_{1}-r_{2}∥}dr_{1}dr_{2}$$

The antisymmetrized reference state abandons the idea that energy components should be relatable to properties of the monomer wave functions, because ${\mathrm{\rho ̃}}_{A}^{total}\left(r\right)$ and ${\mathrm{\rho ̃}}_{B}^{total}\left(r\right)$ depend on the SCF density for the dimer A···B.

To facilitate comparison to SAPT electrostatics, we use the “classical electrostatics” energy (Δ*E*_elec_^cls^) as an alternative definition of electrostatics in the ALMO-EDA2 calculations.^85^ The quantity Δ*E*_elec_^cls^ is defined as in eq 10, using SCF densities for monomers A and B that are obtained from the target functional. The Pauli repulsion term is correspondingly modified (to Δ*E*_Pauli_^mod^), so that

18 $$\Delta E_{frz}=\Delta E_{elec}^{cls}+\Delta E_{Pauli}^{\mathrm{mod}}+\Delta E_{disp}$$

is the same frozen energy as in eq 14, partitioned differently (The dispersion energy is still defined by eq 15.) That Δ*E*_elec_^cls^ was introduced in the context of parametrizing classical force fields^85^ is a concession to the utility of monomer-based definitions of the energy components.

## 3 Methods

### 3.1 Data Sets

Standard data sets for noncovalent interactions are used to compare EDA schemes. The smallest systems considered are the equilibrium S22 and S66 complexes,^124,125^ which range up to 36 atoms for the dimer. SAPT2+(3) benchmarks are available for S22,^84^ which will serve as a check for replacing SAPT2+(3) with SAPT­(KS) + MBD. Benchmark interaction energies at the CCSD­(T)/CBS level are available for both S22 and S66.^124,125^

The S66 data set was designed to cover a more representative swath of intermolecular interactions as compared to S22.^125^ In Figure 1, these complexes are organized into three subsets: hydrogen-bonded dimers, dispersion-bound dimers, and dimers with mixed-influence interactions.^125^ The hydrogen-bonded dimers are dominated by electrostatics, in the sense that

$$\left|E_{elst}\right|\geq 2\left|E_{disp}\right|,\left(electrostatics‐dominated\right)$$ 19

whereas the dispersion-dominated dimers are defined by the opposite criterion

$$\left|E_{disp}\right|\geq 2\left|E_{elst}\right|,\left(dispersion‐dominated\right)$$ 20

If neither of these conditions is met, then the dimer is classified as “mixed influence”.

We also consider the L7 data set,^126^ where the dimers range up to 112 atoms (73 non-hydrogen atoms) and CCSD­(T)/CBS benchmarks are also available.^127,128^ A benchmark comparison of DFT and SAPT + MBD interaction energies for the L7 dimers can be found elsewhere.^62^ Here, we are interested in how the energy components compare.

Finally, we will examine a newer “quantum interacting dimer” (QUID) database of biomolecular dimers representing elements of ligand–protein interactions.^91^ The QUID data set contains 42 equilibrium and 128 nonequilibrium geometries for dimers ranging up to 64 atoms in size, and containing elements {H,N,C,O,F,P,S,Cl}. Intermolecular interactions in the QUID data set are mostly mixed or dispersion-dominated, according to the criteria introduced above; see Figure S1. The 42 parent dimers are classified into folded, semifolded, and linear categories.^91^ Nonequilibrium configurations are generated for 16 of these dimers by scanning along an intermolecular separation coordinate R = *qR*_e_, where *R*_e_ is the equilibrium separation and the scale factor *q* = 0.9–2.0. Benchmark interaction energies are available,^91^ computed at the CCSD­(T)/CBS level using a localized natural orbital (LNO) approximation.^129^

### 3.2 Quantum Chemistry Calculations

Most calculations were performed using Q-Chem v. 6.3,^130^ for which the default version of ALMO-EDA is the second-generation method. A few calculations using the D4 dispersion model^131^ were performed using Q-Chem v. 7.0. Sample Q-Chem input files for both the ALMO-EDA2 calculations and the SAPT + MBD calculations are provided in Figures S2–S4.

#### 3.2.1 Density Functionals

The main purpose of this work is to make connections between energy components computed using SAPT versus ALMO-EDA2. For that purpose, we test two different DFXC functionals in eq 15, namely, the HF functional (exact exchange and no correlation), as recommended elsewhere for this purpose,^25,52^ and also the “dispersionless” functional dlDF.^120^

For the target functional in the ALMO-EDA2 calculations, we select from a cross section of choices that perform well for intermolecular interaction energies in small dimers, as gauged in a recent survey.^62^ Specifically, we examine the dispersion-inclusive functionals ωB97M-V^132^ and M06-2X,^133^ along with dispersion-corrected functionals including ωB97X-D,^134^ BLYP + D3­(BJ), PBE0+D3­(BJ), and B3LYP + D3­(BJ). Here, “D3­(BJ)” indicates the D3 dispersion correction^135^ with Becke-Johnson (BJ) damping.^136^ The SG-2 quadrature grid^137^ is used for the B97-based functionals, the SG-3 grid^137^ is used for M06-2X, and the SG-1 grid^138^ is used for other functionals.

Among the target functionals tested, BLYP is the only one that is not a hybrid. For semilocal functionals, it has been recommended to use revPBE^119^ as the dispersion-free functional.^25,52^ For consistency, however, we shall only use HF and dlDF as the DFXC, for all target functionals. For molecular quantum chemistry applications, hybrid functionals are significantly better options than semilocal functionals and our primary interest lies in this type of application.

SAPT­(KS) + MBD calculations employ the long-range corrected (LRC) functional LRC-ωPBE.^139^ To emphasize this, we will refer to the composite SAPT approach as SAPT­(LRC-ωPBE) + MBD. For SAPT­(KS) calculations on the S22, S66, and L7 data sets, the range-separation parameter ω in LRC-ωPBE is adjusted in a monomer-specific way using the global density-dependent (GDD) tuning procedure.^97,140,141^ This ensures proper asymptotic behavior of the exchange-correlation potential for each monomer, which is important when KS orbitals and energy levels are inserted into the SAPT formalism.^142^ For calculations on the QUID data set, we used fixed values of ω to understand its effect. Range-separation parameters used for each data set are given in Tables S3–S5.

#### 3.2.2 Basis Sets

All ALMO-EDA calculations are counterpoise-corrected to eliminate BSSE. Calculations for the S22, S66, and L7 data sets employ the def2-QZVPD basis set^143^ and should be very close to the DFT/CBS limit.^144^

In practical calculations for larger molecules, def2-QZVPD may not be feasible. In an effort to understand the basis-set dependence of the ALMO-EDA versus SAPT­(LRC-ωPBE) + MBD comparison, we examine results for the QUID dimers using a sequence of Karlsruhe basis sets ranging from def2-SVP to def2-ma-TZVP, where the latter is a minimally augmented (“ma”) version of def2-TZVP obtained by systematically pruning the diffuse functions with higher angular momenta.^145^ Similarly, def2-ma-SVP is a minimally augmented version of def2-SVP and a proper subset of def2-SVPD.^145^

## 4 Results and Discussion

We begin our analysis with a thorough comparison of dispersion energies (Section 4.1), establishing the extent to which MBD and ALMO-EDA2 dispersion agree with one another and with SAPT2+(3) dispersion. Other energy components are considered in Section 4.2. In both cases, ALMO-EDA2 is tested using a variety of density functionals. In Section 4.3, we examine the QUID dimers in detail using SAPT­(LRC-ωPBE) + MBD and ALMO-EDA2­(ωB97M-V).

### 4.1 Dispersion Energies

In a recent comparison between SAPT and two different DFT-based EDA schemes, dispersion was found to exhibit the largest deviations between the two approaches.^53^ Thus, we first consider dispersion in detail. For each of the six target functionals that are listed in Table 1, we test ALMO-EDA2 with two different choices for the DFXC functional (dlDF or HF) that is used to isolate the dispersion energy via eq 15.

**Table 1:** Deviation Between ALMO-EDA2 and SAPT2+(3) Dispersion for the S22 Dimers^a^

target functional	mean absolute deviation			
	DFXC = dlDF		DFXC = HF	
	(kcal/mol)	(%)	(kcal/mol)	(%)
M06-2X	0.64	9.4	1.66	23.1
ωB97M-V	0.58	7.7	1.26	17.4
ωB97X-D	0.66	10.4	1.31	17.4
PBE0+D3(BJ)	0.47	7.0	1.26	15.6
B3LYP + D3(BJ)	0.48	6.9	0.94	12.3
BLYP + D3(BJ)	0.67	10.3	1.16	17.4
MBD^b^	0.71	13.8		

a SAPT2+(3)/aug-cc-pVTZ (ref 84) versus ALMO-EDA2/def2-QZVPD.

b SAPT­(LRC-ωPBE) + MBD/def2-QZVPD, which does not require a DFXC functional but is included here for comparison.

This analysis will proceed in stages. In Section 4.1.1, we make comparison to SAPT2+(3)/aug-cc-pVTZ benchmarks for the S22 data set.^84^ This will establish that SAPT­(LRC-ωPBE) + MBD affords an adequate proxy for SAPT2+(3) dispersion that can be applied to much larger systems, as its efficacy is *O*(*N* ^3^) rather than *O*(*N* ^7^). Then, we compare ALMO-EDA2 and SAPT­(LRC-ωPBE) + MBD dispersion energies for the S66 data set (Section 4.1.2) and for the L7 data set (Section 4.1.3). These comparisons afford somewhat contradictory results, so in Section 4.1.4 we turn to the larger QUID data set for a resolution.

#### 4.1.1 Comparison to SAPT2+(3) Using S22

The ωB97M-V functional is the best all-around method for noncovalent interactions in contemporary DFT, according to small-molecule benchmarks.^146^ Figure 2a correlates ALMO-EDA2­(ωB97M-V) dispersion energies against SAPT2+(3) values. Using dlDF to define dispersion in ALMO-EDA2 affords excellent correlation with SAPT2+(3) dispersion energies (*R* ^2^ = 0.964), with a near-unit slope and a very small offset of 0.2 kcal/mol. The correlation is degraded (*R* ^2^ = 0.931) when the recommended choice of HF is used for the DFXC functional, with a slope of 0.8 indicating that ALMO-EDA2 dispersion is about 20% less attractive than SAPT2+(3) dispersion in this case.

**Figure 2:**
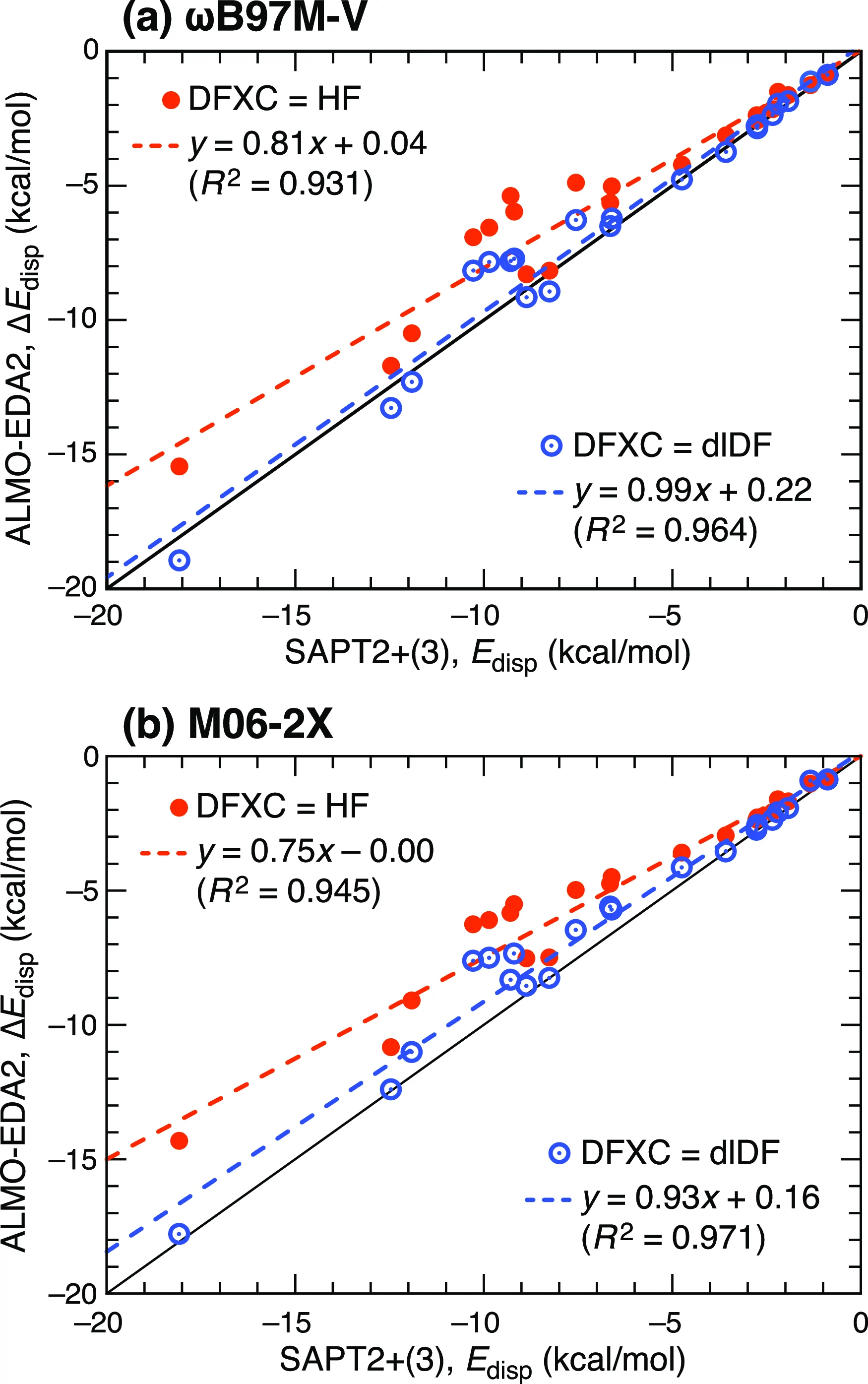
Dispersion energies for the S22 dimers, correlating SAPT2+(3)/aug-cc-pVTZ and ALMO-EDA2/def2-QZVPD values where the latter are calculated with either (a) ωB97M-V or (b) M06-2X as the target functional. In either case, results are presented using either of two DFXC functionals. Linear fits to both data sets are shown as broken lines, with the solid line indicating *y* = *x*. SAPT2+(3) data are taken from ref 84.

Whereas ωB97M-V is interesting due to its superior performance for noncovalent interactions, M06-2X is an interesting test because the dlDF functional was parametrized using the same functional *ansatz*.^120^ Therefore, one might expect correlations with SAPT dispersion to be near-optimal for the pairing of M06-2X as the target functional and dlDF as the DFXC functional. The correlation is certainly very good, with *R* ^2^ = 0.971 and a linear regression slope of 0.93 (Figure 2b), although not as good as when ωB97M-V is the target functional. This inspires some confidence that the near-unit slope observed for pairing ωB97M-V with dlDF is not simply a result of the fact that dlDF was fit using SAPT dispersion data.

Plots analogous to those in Figure 2, for four other target functionals, can be found in Figure S6. A statistical summary is provided in Table 1 and linear regression parameters can be found in Table S1. Slopes of the linear fits range from 0.91–1.01 when dlDF is the DFXC functional, indicating excellent correlation across a range of target functionals. Mean absolute differences (MADs) with respect to SAPT2+(3) dispersion energies are smaller than 0.7 kcal/mol (≲ 10%).

Differences are larger when HF is selected as the DFXC functional, ranging from 12%–23% of the dispersion energy itself, depending on the target functional. Linear regression indicates that ALMO-EDA2 dispersion is 16%–25% less attractive as compared to SAPT2+(3) dispersion in this case. Intercepts in these fits are smaller than 0.2 kcal/mol in magnitude (Table S1), except for PBE0+D3­(BJ) where the intercept is −0.4 kcal/mol.

ALMO-EDA2 dispersion energies are computed using a quasiclassical version of electrostatics based on isolated-monomer charge densities (Section 2.3.2). This will continue to be our basis for comparison to SAPT, although we pause here to note that ALMO-EDA2 dispersion energies for the S22 complexes are not significantly affected if distorted charge densities (stemming from an antisymmetrized reference state) are used instead. These differences are summarized in Table S2, which is analogous to Table 1 but uses distorted monomer densities for ALMO-EDA2. However, this similarity for the S22 data set is likely an artifact of small monomer size and relatively weak interactions for those particular complexes, and should not be taken as a general result.

For the larger QUID dimers, the use of an antisymmetrized reference for ALMO-EDA2 results in significant discrepancies with SAPT, not just for electrostatics but propagating also into Pauli repulsion. The agreement between *E*_exch_^SAPT(KS)^ (eq 5b) and Δ*E*_Pauli_^mod^ (eq 18) is much improved when the quasiclassical electrostatics definition is used; see Figure S7. Thus, energy components that employ an antisymmetrized (Heitler–London) reference should not be compared to SAPT energy components, even if there are deceptively strong correlations between the two in certain cases.

SAPT2+(3) benchmarks are typically unavailable for systems much larger than the S22 dimers. Fortunately, MBD and SAPT2+(3) dispersion correlate quite well, as shown in Figure 3. As such, we will use the dispersion energy from SAPT­(LRC-ωPBE) + MBD as a point of reference for SAPT dispersion in larger complexes.

**Figure 3:**
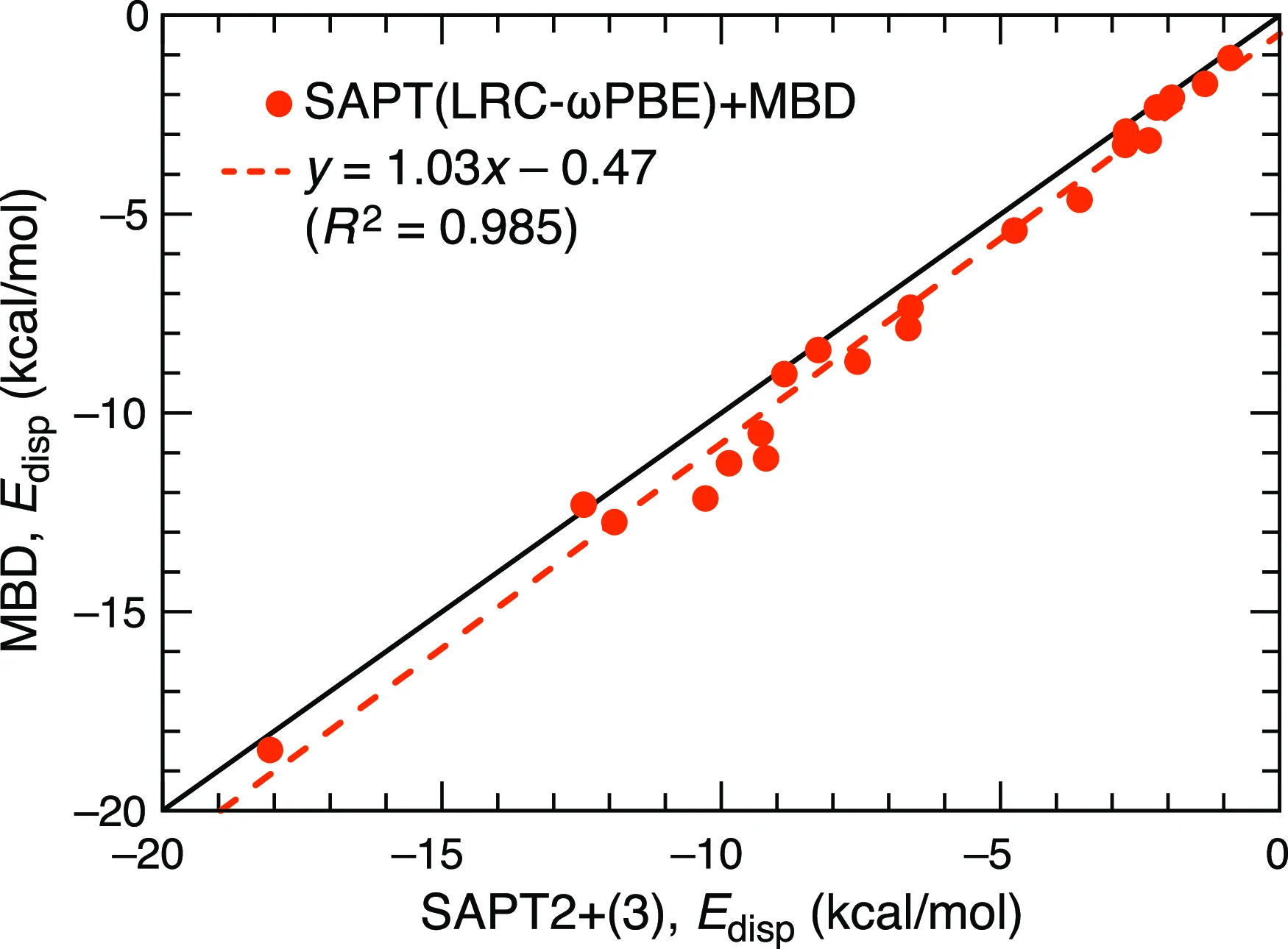
Dispersion energies for the S22 dimers, illustrating the correlation between SAPT2+(3)/aug-cc-pVTZ data (from ref 84) and SAPT­(LRC-ωPBE) + MBD/def2-QZVPD values.

#### 4.1.2 Comparison to MBD for S66

For the S66 dimers, we analyze correlations between MBD (eq 7) and ALMO-EDA2 dispersion. These are shown in Figure 4, with ωB97M-V as the target functional for ALMO-EDA2; analogous plots for other functionals are provided in Figure S8. Differences between MBD and ALMO-EDA2 dispersion are summarized in Table 2 for all functionals tested.

**Figure 4:**
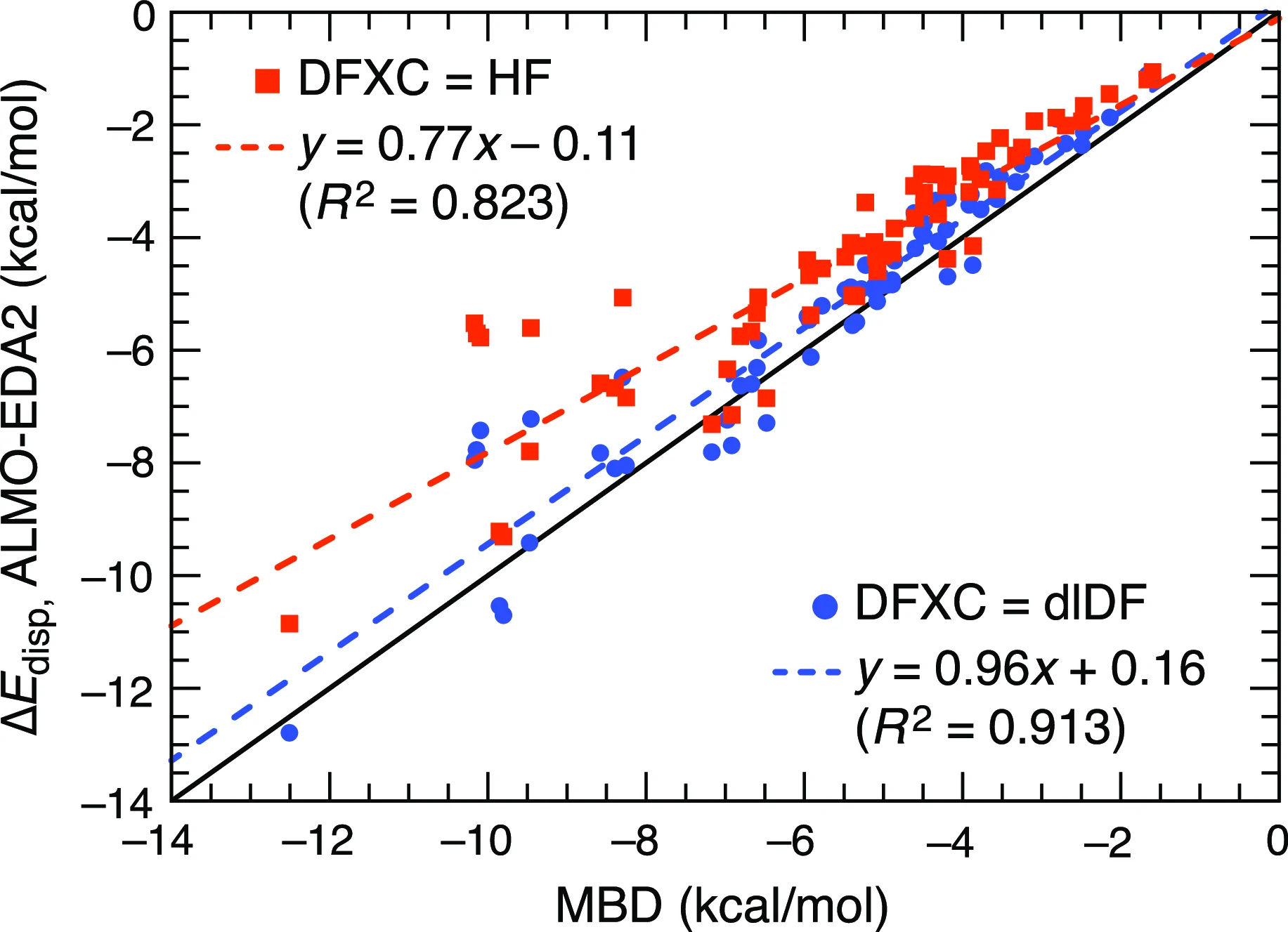
Dispersion energies for the S66 dimers, correlating MBD with ALMO-EDA2­(ωB97M-V) dispersion. The latter uses either HF or dlDF as the dispersion-free functional (as indicated), and linear fits to either data set are shown. All calculations use the def2-QZVPD basis set.

**Table 2:** Differences Between MBD and ALMO-EDA2 Dispersion for the S66 Dimers^a^

	DFXC = dlDF					DFXC = HF				
	MAD^b^		linear regression			MAD^b^		linear regression		
target functional	(kcal/mol)	(%)	slope	intercept (kcal/mol)	*R* ^2^	(kcal/mol)	(%)	slope	intercept (kcal/mol)	*R* ^2^
M06-2X	0.77	14.8	0.88	+0.08	0.933	1.53	27.8	0.69	–0.18	0.856
ωB97M-V	0.59	11.4	0.96	+0.16	0.913	1.19	22.0	0.77	–0.11	0.823
ωB97X-D	0.62	12.1	0.96	+0.07	0.879	1.11	21.3	0.78	–0.17	0.763
PBE0+D3(BJ)	0.46	8.3	0.90	–0.20	0.945	1.12	19.6	0.73	–0.43	0.852
B3LYP + D3(BJ)	0.45	8.2	0.99	+0.11	0.937	0.97	17.8	0.81	–0.14	0.852
BLYP + D3(BJ)	0.69	13.5	0.98	+0.35	0.894	1.24	23.5	0.80	+0.07	0.781

a All calculations use the def2-QZVPD basis set.

b Mean absolute deviation.

MADs between ALMO-EDA2 dispersion energies and MBD values are comparable to the differences between ALMO-EDA2 and SAPT2+(3) dispersion energies. Percentage differences are somewhat larger but still within 15% for each functional tested, when dlDF is used as the dispersion-free functional. In that case, linear regressions afford slopes of 0.9–1.0, with *R* ^2^ ≥ 0.88 and intercepts that are small. In particular, the ωB97M-V functional affords a slope of 0.96, with *R* ^2^ = 0.913 and an intercept of only 0.2 kcal/mol. This indicates very close agreement with the MBD model for dispersion.

Changing the dispersion-free functional to HF degrades these correlations and increases the absolute difference between ALMO-EDA2 and MBD. Mean relative differences approach 30% in this case, and linear regressions exhibit slopes of 0.7–0.8, meaning that ALMO-EDA2 dispersion is 20%–30% less attractive than MBD. Correlation coefficients are also smaller than they were when dlDF was used to isolate the ALMO-EDA2 dispersion energy. Combined with the S22 results from Section 4.1.1, these data point toward dlDF as a better way to isolate dispersion in ALMO-EDA2 calculations, as compared to the HF functional. However, we will see that the picture is more ambiguous for the L7 complexes that are discussed next.

#### 4.1.3 L7 Complexes

Dispersion energies for the S22 and S66 dimers are relatively small, with |*E*_disp_|≲ 5 kcal/mol for more than 2/3 of the S66 dimers and |*E*_disp_|≲ 10 kcal/mol for all but three of the S22 complexes. To increase the diversity of the test set, we turn to the larger complexes in L7.^126^ Here, benchmark CCSD­(T)/CBS interaction energies (based on local orbital approximations) range up to |Δ*E*_int_| = 29 kcal/mol for the guanine–cytosine base pair atop circumcoronene (“C3GC”).^127,128^ For comparison, the largest interaction energy in either S22 or S66 is |Δ*E*_int_| = 20 kcal/mol, for acetic acid in a hydrogen-bonded complex with uracil.^147^ Dispersion energies are also larger in magnitude for L7 (see Figure S9), ranging from |*E*_disp_| ≈ 10 kcal/mol for the guanine trimer (GGG, which is treated as a dimer of G and GG) up to |*E*_disp_| ≈ 50 kcal/mol for C3GC. In contrast, the largest value of |*E*_disp_| in either S22 or S66 is 18 kcal/mol, for the π-stacked adenine–thymine dimer.

Larger dispersion energies are reflected in larger differences between MBD and ALMO-EDA2 dispersion, as summarized in Figure 5. (Results for the individual L7 complexes can be found in Figure S10.) When the DFXC functional is set to dlDF, the difference

21 $$\Delta_{disp}=\Delta E_{disp}^{ALMO‐EDA2}-E_{disp}^{SAPT+MBD}$$

spans a wide range, from −8 kcal/mol to +14 kcal/mol. That said, most differences lie within ±5 kcal/mol except for the pairing of M06-2X with HF.

**Figure 5:**
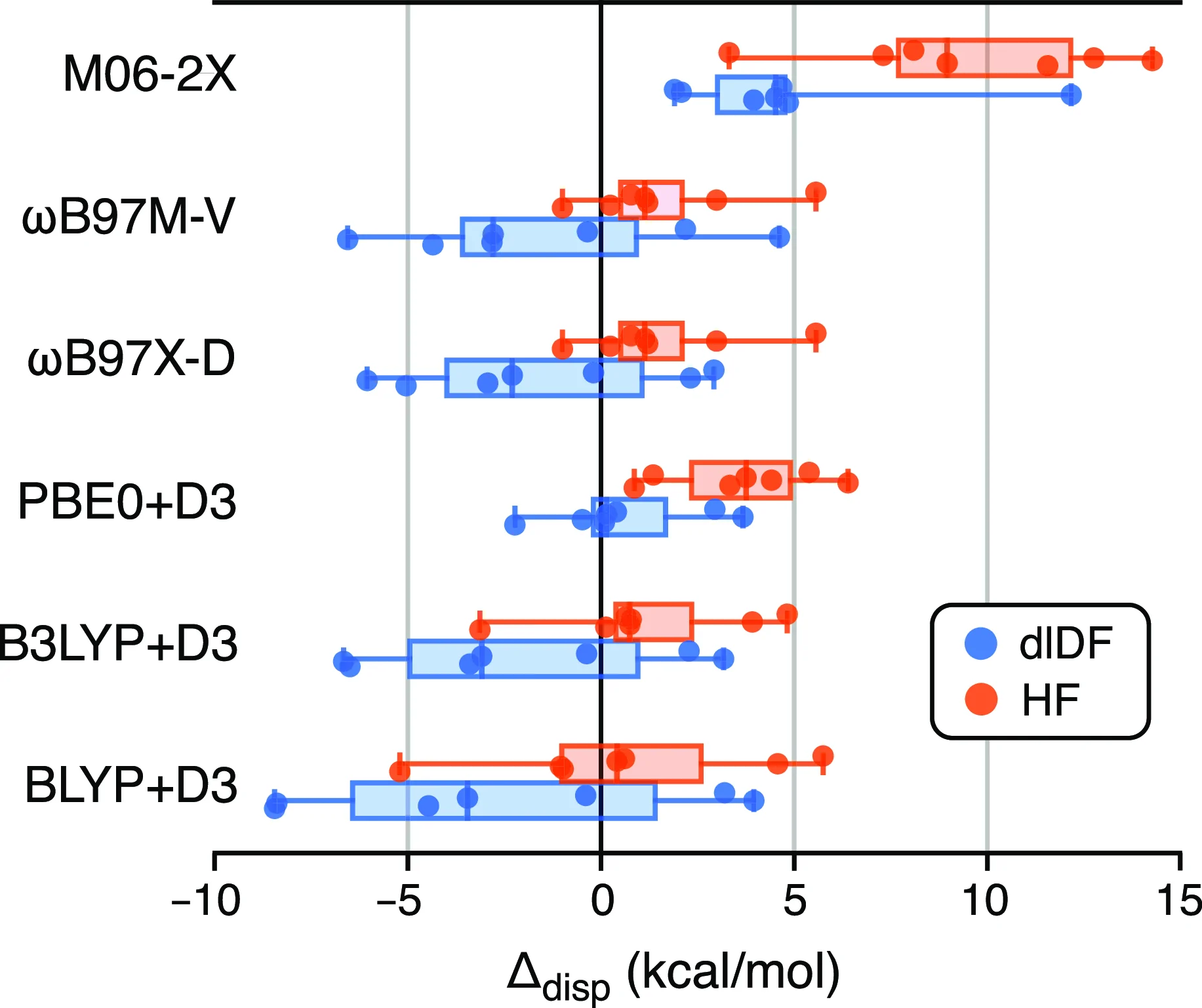
Difference Δ_disp_ (eq 21) between MBD and ALMO-EDA2 dispersion energies for the L7 complexes, using either dlDF or HF as the DFXC functional in the ALMO-EDA2 calculations. Boxes show quartiles and medians across the L7 complexes, and filled circles show the individual data. All calculations use the def2-QZVPD basis set.

Statistics for Δ_disp_ among the L7 data are summarized in Table 3. These differences are larger than they were for the S22 or S66 dimers, reflecting the fact that dispersion is extensive and thus larger for the L7 complexes. When dlDF is used to define ALMO-EDA2 dispersion, differences between S66 and L7 results are rather comparable, functional by functional, although somewhat smaller for PBE0+D3­(BJ). For the HF-based definition of dispersion, there is more variation between S66 and L7 results. Here, M06-2X results for L7 are an outlier at 36% deviation from MBD.

**Table 3:** Summary of Δ_disp_ (eq 21) for the L7 Complexes^a^

	mean absolute deviation			
target functional	DFXC = dlDF		DFXC = HF	
	(kcal/mol)	(%)	(kcal/mol)	(%)
M06-2X	4.9	20.0	9.5	35.6
ωB97M-V	3.4	11.4	1.8	10.6
ωB97X-D	3.1	10.8	1.8	10.9
PBE0+D3(BJ)	1.4	6.6	3.6	16.6
B3LYP + D3(BJ)	3.6	11.5	2.0	10.8
BLYP + D3(BJ)	4.6	15.1	2.7	10.9

a All calculations use the def2-QZVPD basis set.

#### 4.1.4 QUID Results and Transferability

Results for S22 and S66 suggest using dlDF to obtain the best agreement between MBD and ALMO-EDA2 dispersion but the same recommendation cannot be made for the L7 complexes, except in the anomalous case of the PBE0+D3­(BJ) functional. Figure S11 summarizes statistical differences between SAPT and ALMO-EDA2 dispersion for these three data sets.

In view of this ambiguity, we turn to the more numerous QUID data to help determine which dispersion-free functional affords better agreement between MBD and ALMO-EDA2 dispersion energies. The QUID set of biomolecular fragments contains structures with intermolecular separations smaller than the equilibrium distance, so these tests also examine stronger overlap and confinement effects. For these tests, we will use basis sets that are more realistic for everyday applications, meaning def2-ma-TZVP or smaller, rather than the quadruple-ζ basis sets that were used above. For simplicity, we choose ωB97M-V as the target functional for ALMO-EDA2.

Figure 6 shows a side-by-side comparison of ALMO-EDA2 and MBD dispersion for the QUID dimers, for two different choices of the DFXC functional. Although these data were computed using the def2-ma-TZVP basis set, that choice hardly matters and def2-SVP affords nearly identical results (Figure S12). This is consistent with a very weak basis-set dependence for MBD,^145^ because *E*_disp_ converges like the density rather than like the wave function in this model. The equally mild basis-set dependence of Δ*E*_disp_ in the ALMO-EDA2 calculations is perhaps a surprise.

**Figure 6:**
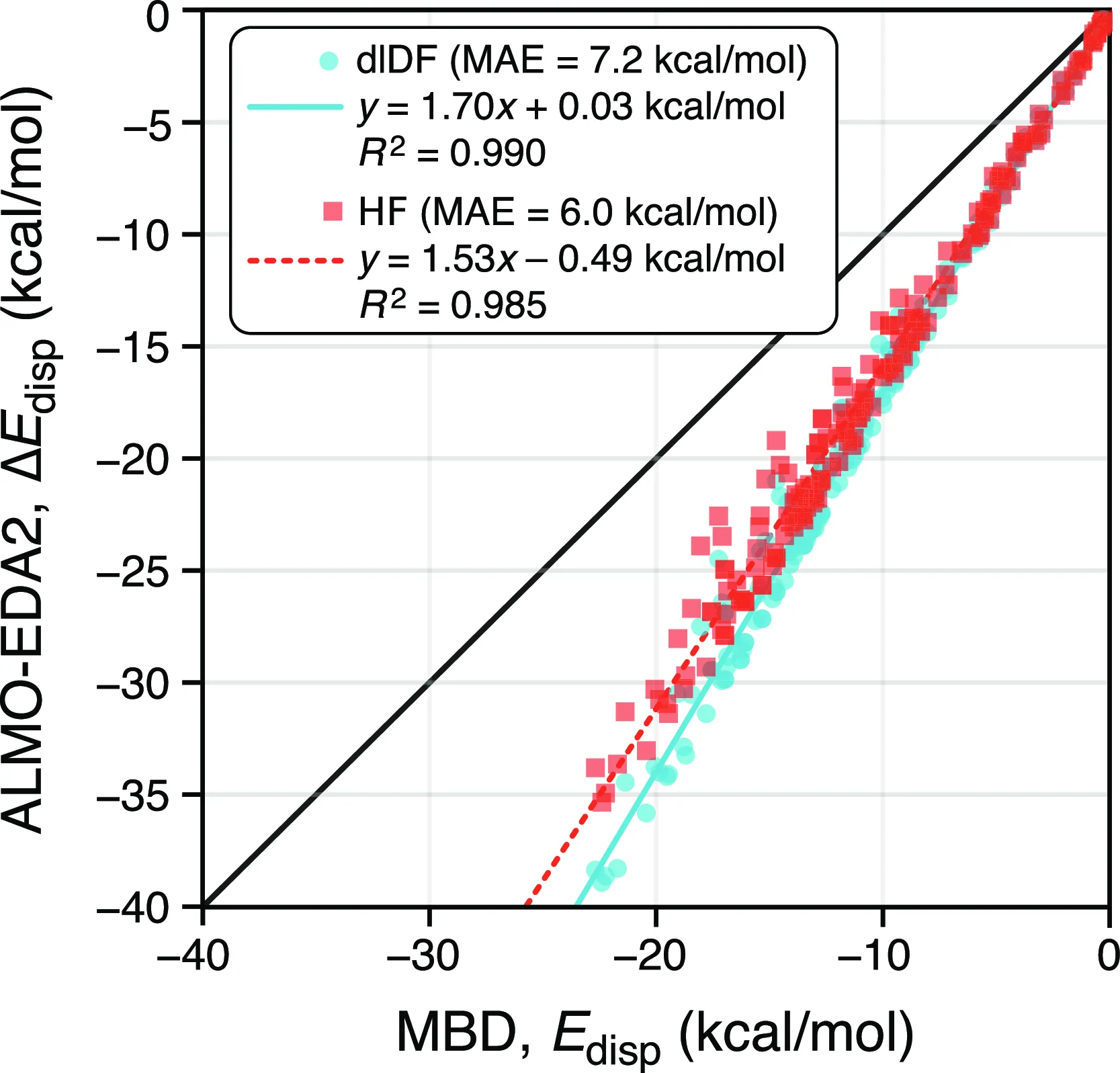
Dispersion energies for the QUID dimers, correlating SAPT­(LRC-ωPBE) + MBD with ALMO-EDA2­(ωB97M-V). The latter uses a quasiclassical reference state and either HF or dlDF as the DFXC functional, as indicated. All calculations use the def2-ma-TZVP basis set. Linear fits are shown along with the *y* = *x* line.

Dispersion energies for the QUID data set span a larger range as compared to the S66 dimers, and while they are not so large (in magnitude) as the L7 dispersion energies, the QUID data are far more numerous. They make it clear that choosing HF for the dispersion-free functional affords slightly better agreement with the MBD model (MAD = 6.0 kcal/mol) than does dlDF (MAD = 7.2 kcal/mol). That said, the difference between these two MADs is smaller than the variation amongst the data. For example, when *E*_disp_^MBD^ = −20 kcal/mol the ALMO-EDA2 dispersion energy is more attractive by ≈ 11 kcal/mol (for HF) or by ≈ 14 kcal/mol (for dlDF). That DFXC = dlDF affords a more attractive dispersion energy as compared to DFXC = HF is inconsistent with the idea that the latter is ascribing too much correlation to dispersion, because it labels *all* correlation as dispersion.^62^ It may simply be the case that the M05-2X functional form that is used for dlDF is not fully compatible with the target functional (ωB97M-V), leading to incomplete cancellation of correlation effects. In any case, results for this larger data set do not justify a change in the recommendation to set DFXC = HF when the target functional is a hybrid.

### 4.2 Other Energy Components

Having performed a detailed analysis of differences between ALMO-EDA2 and SAPT dispersion energies, we next consider other energy components. For dispersion energies, trends gleaned from S66 data were somewhat different than those based on QUID data, so this broader analysis is separated along the same lines. S66 results are presented in Section 4.2.1 and QUID results in Section 4.2.2. The ωB97M-V functional is used for the ALMO-EDA2 calculations in this section, and we assume that the ALMO-EDA2 polarization energy (Δ*E*_pol_ in eq 13) represents the same physics as SAPT induction, so that it makes sense to plot one versus the other. No attempt is made to isolate a charge-transfer component from either the induction or polarization energies.

#### 4.2.1 S66 Dimers

Electrostatics, induction, and Pauli repulsion are plotted in Figure 7 for the S66 dimers, correlating ALMO-EDA2­(ωB97M-V) values with SAPT­(LRC-ωPBE) + MBD results. Near-perfect correlation is found for electrostatics (Figure 7a), consistent with identical definitions when the quasiclassical reference state is used for ALMO-EDA2. Differences in electrostatics can then arise only from differences in the electron densities, computed using ωB97M-V for ALMO-EDA2 versus LRC-ωPBE for SAPT­(KS). These differences are evidently minuscule in energetic terms. Differences in Pauli repulsion energies are also extremely small (Figure 7b), and both components exhibit far less scatter than observed in the corresponding dispersion energies (Figure 4). This is reflected in correlation coefficients *R* ^2^ ≥ 0.995 for electrostatics and Pauli repulsion versus *R* ^2^ = 0.82–0.91 for dispersion.

**Figure 7:**
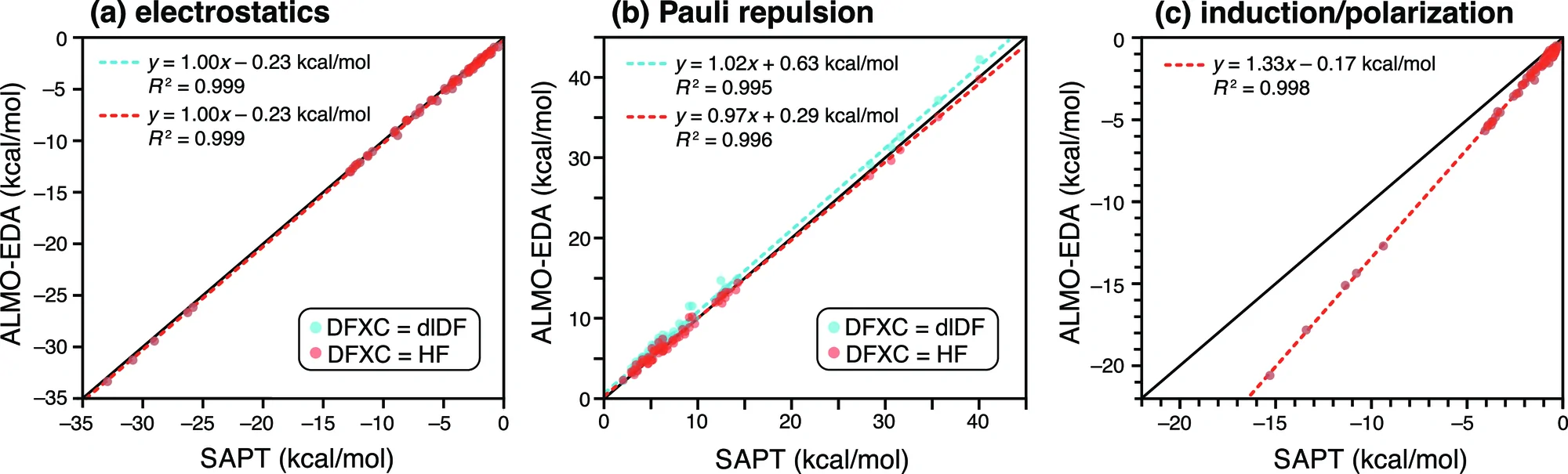
Energy components for the S66 dimers, correlating ALMO-EDA2­(ωB97M-V) and SAPT­(LRC-ωPBE) + MBD for (a) electrostatics, (b) exchange or Pauli repulsion, and (c) induction or polarization (For dispersion, see Figure 4). For SAPT, the energy components are the ones defined in eq 5 and do not include response terms. ALMO-EDA2 calculations use quasiclassical electrostatics (eq 18), with two different choices for the DFXC functional. (Note that the ALMO-EDA2 polarization energy does not depend upon the choice of DFXC functional.) All calculations use the def2-QZVPD basis set.

Induction or polarization energies also exhibit very little scatter but systematic differences are larger (Figure 7c). A linear regression affords *R* ^2^ = 0.998 with a slope of 1.33, meaning that |*E*_ind_| = 15 kcal/mol for SAPT translates into slightly more than 20 kcal/mol for ALMO-EDA2. This is the largest value of |*E*_ind_| in the S66 set and occurs for the acetic acid dimer, which also has the second-largest value of |*E*_int_|. The most strongly interacting S66 complex is the acetic acid–uracil complex, for which energy components are given in Table 4 and discussed below.

**Table 4:** Energy Components for the Acetic Acid–Uracil Complex

comp	energy (kcal/mol)				
	SAPT^a^	ALMO^b^		Δ^c^	
		HF	dlDF	HF	dlDF
electrost	–30.8	–31.3	–31.3	–0.5	–0.5
Pauli repul	35.7	35.1	37.2	–0.6	1.5
induction	–13.4	–17.8	–17.8	–4.4	–4.4
dispersion	–10.1	–5.7	–7.8	4.4	2.4
total int	–18.7	–19.7	–19.7	–1.0	–1.0

a SAPT­(LRC-ωPBE) + MBD/def2-QZVPD.

b ALMO-EDA2­(ωB97M-V)/def2-QZVPD.

c Difference between ALMO and SAPT, defined in the sense of eq 21 for dispersion.

In contemplating differences between SAPT and ALMO energy components, it is worth noting that there is little difference between the total interaction energies computed using either method; see Figure 8. (Use of def2-QZVPD is intended to eliminate any basis-set effects from the comparison.) This agreement is consistent with the generally similar performance of most dispersion-corrected and dispersion-inclusive DFT methods for small dimers such as the S66 complexes.^62,148^ That similarity breaks down in much larger complexes with 100+ atoms and conjugated monomers.^62^

**Figure 8:**
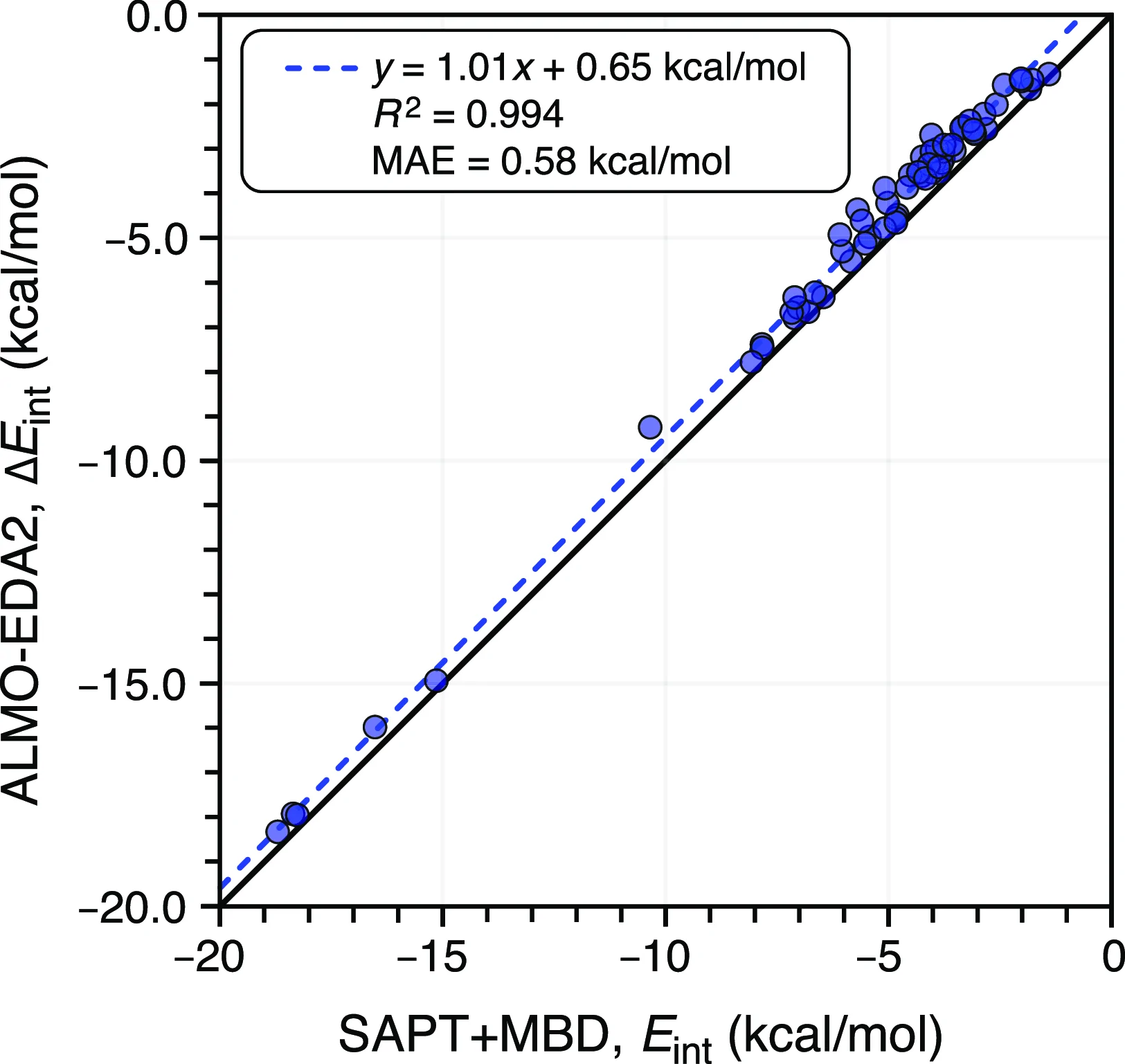
Total interaction energies for the S66 dimers, correlating ALMO-EDA2­(ωB97M-V) results with SAPT­(LRC-ωPBE) + MBD values. The solid line represents *y* = *x* and the dashed line is a linear regression. All calculations employ the def2-QZVPD basis set.

A linear regression correlating the ωB97M-V and SAPT + MBD interaction energies in Figure 8 has a slope of 1.01 and an intercept of 0.65 kcal/mol. Even for the most strongly bound S66 dimers (e.g., the six data points with |*E*_int_|≳ 10 kcal/mol), the two methods are in good agreement despite the fact that ALMO-EDA2 induction is 33% more stabilizing than SAPT induction, according to the linear fit in Figure 7c. Thus, some kind of compensation mechanism must exist among the energy components that emerge from ALMO-EDA2 versus SAPT + MBD.

Table 4 presents an example of such a compensation for the acetic acid–uracil complex . This is the most strongly bound of the S66 complexes, and the ωB97M-V and SAPT + MBD interaction energies agree to within 1.0 kcal/mol. However, ALMO-EDA2 induction is more attractive by Δ_ind_ = −4.4 kcal/mol (defined analogously to Δ_disp_ in eq 21). Depending on the choice of DFXC functional, this is partly or fully compensated by the fact that ALMO-EDA2 dispersion is less attractive as compared to MBD, with Δ_disp_ = 4.4 kcal/mol for DFXC = HF and Δ_disp_ = 2.4 kcal/mol for DFXC = dlDF. The difference in dispersion energies between these two ALMO-EDA2 calculations manifests as a difference in Pauli repulsion, which is more repulsive when dlDF is used and dispersion is more attractive. As a result, the difference Δ_Pauli_ with respect to SAPT exchange repulsion changes sign, depending on the choice of DFXC functional for ALMO-EDA2.

#### 4.2.2 QUID Complexes

Energy components for the QUID complexes are plotted in Figure 9 for calculations using the def2-SVP basis set. This basis set mirrors common practice in many large DFT calculations, although SAPT exchange and induction are often not fully converged in double-ζ basis sets.^145^ Even counterpoise-corrected supramolecular DFT interaction energies may not be converged to sub-kcal/mol levels in double-ζ basis sets, unless diffuse functions are included.^144^ Thus, the same comparisons are plotted in Figure 10 for calculations using def2-ma-TZVP, which is typically converged for both supramolecular DFT^144^ and for SAPT + MBD.^145^ (For side-by-side comparisons between the two basis sets, see Figures S13–S18.) SAPT + MBD calculations for the QUID dimers use fixed values of ω, as discussed in Section 3.2.1.

**Figure 9:**
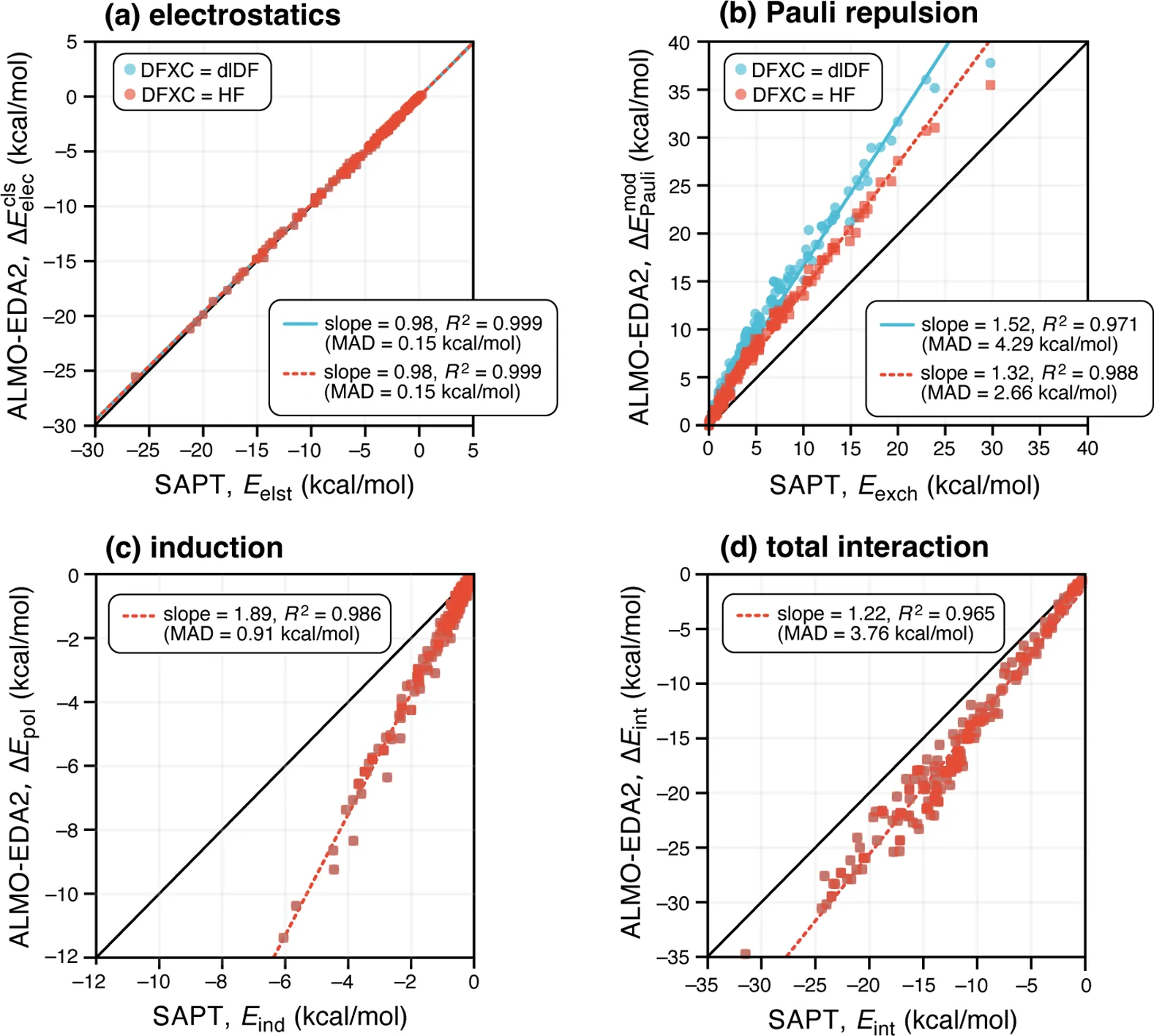
Energy components and total interaction energies for the QUID dimers, correlating ALMO-EDA2­(ωB97M-V) and SAPT­(LRC-ωPBE) + MBD results computed using the def2-SVP basis set for (a) electrostatics, (b) Pauli repulsion, (c) induction or polarization, and (d) the total interaction energy. ALMO-EDA2 results use a quasiclassical reference state and SAPT induction does not contain response terms. Each plot contains 186 data points and linear regressions are shown. Black diagonal lines indicate *y* = *x*.

**Figure 10:**
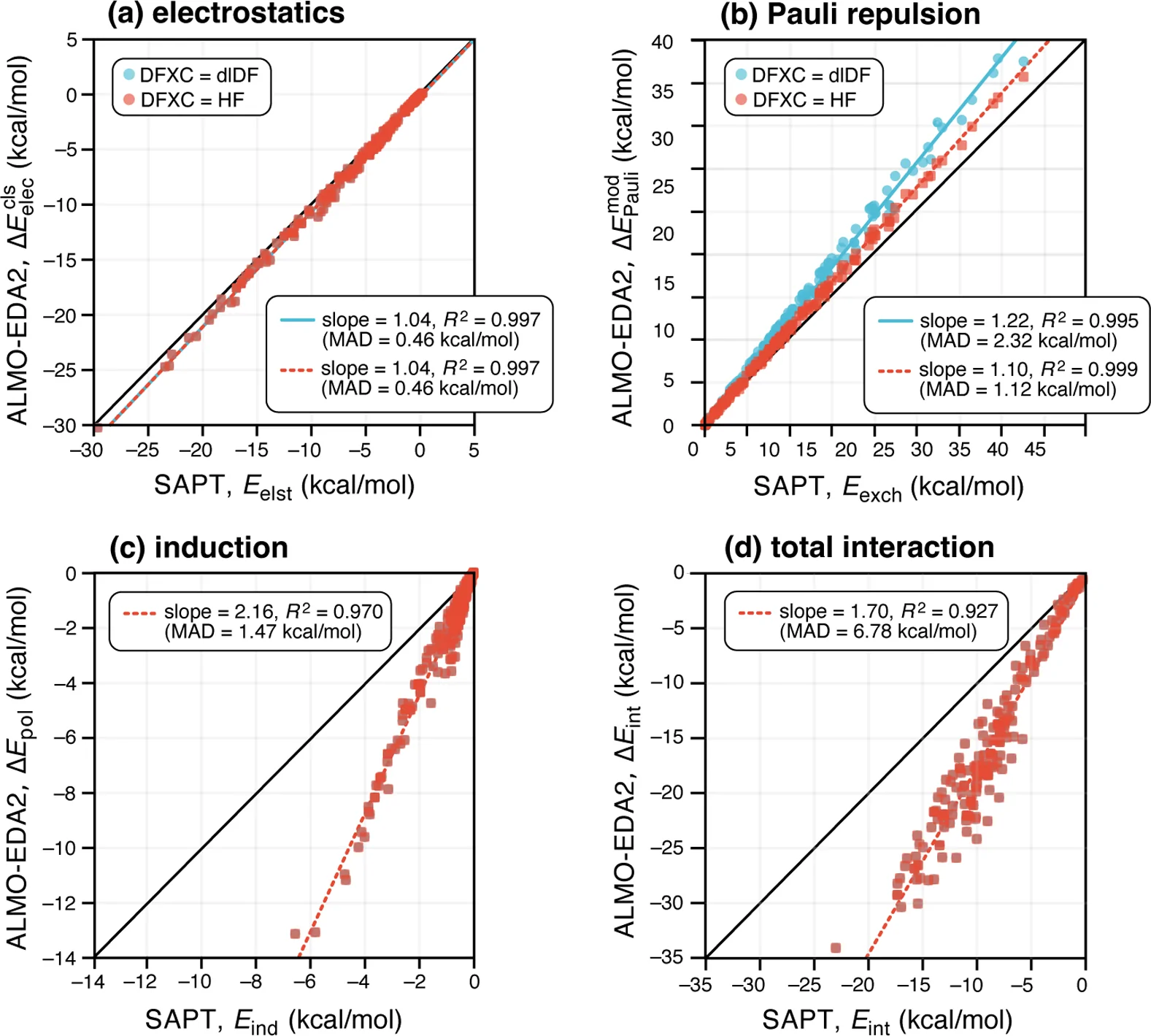
Energy components and total interaction energies for the QUID dimers, correlating ALMO-EDA2­(ωB97M-V) and SAPT­(LRC-ωPBE) + MBD results computed using the def2-ma-TZVP basis set for (a) electrostatics, (b) Pauli repulsion, (c) induction or polarization, and (d) the total interaction energy (For dispersion, see Figure 6.) ALMO-EDA2 results use a quasiclassical reference state and SAPT induction does not contain response terms. Each plot contains 186 data points and linear regressions are shown. Black diagonal lines indicate *y* = *x*.

Whereas ωB97M-V and SAPT + MBD interaction energies agreed to within a MAD of 0.6 kcal/mol for the S66 complexes (Figure 8), differences are larger for the QUID dimers. The MAD in total interaction energies increases from 3.8 kcal/mol for def2-SVP calculations (Figure 9d) to 6.8 kcal/mol for def2-ma-TZVP (Figure 10d). Results presented in Section 4.3 suggest that the def2-ma-TZVP results are converged.

As with the S66 data, electrostatic energies are in excellent agreement between the two EDA methods, with MADs below 0.5 kcal/mol across a range spanning 30 kcal/mol. Although the ALMO-EDA2 results presented in Figures 9a and 10a use the quasiclassical reference state, the antisymmetrized reference state also affords very good correlation with SAPT electrostatics, with *R* ^2^ = 0.94 (Figure S13). In the antisymmetrized case, however, the slope of the linear regression is much larger: 1.77 as compared to slopes near unity in Figures 9a and 10a. This translates into much larger differences between ALMO-EDA2 and SAPT electrostatics, with a MAD of 7 kcal/mol when the antisymmetrized reference state is used as compared to agreement within 0.5 kcal/mol for the quasiclassical reference state.

Pauli repulsion energies (Figures 9b and 10b) exhibit somewhat more scatter than they did for S66. Nevertheless, the MAD for DFXC = HF decreases from 2.7 kcal/mol in def2-SVP to 1.1 kcal/mol in def2-ma-TZVP, for repulsion energies up to about 40 kcal/mol. The corresponding MADs are 4.3 and 2.3 kcal/mol for DFXC = dlDF. These increases for dlDF are consistent with the picture gleaned from the acetic acid–uracil complex (Table 4), for which dlDF affords more attractive dispersion that is compensated by an increase in Pauli repulsion, moving the latter away from the corresponding SAPT value.

As with electrostatics, good correlations with SAPT exchange-repulsion remain even when the definition of electrostatics is antisymmetrized, with *R* ^2^ > 0.93 as shown in Figure S14, although numerical values are very different. Statistical differences between the two (i.e., between Δ*E*_Pauli_ in eq 14 versus Δ*E*_Pauli_^mod^ in eq 18) are summarized in Figure S7. For DFXC = dlDF, the quasiclassical definition reduces the discrepancy between ALMO-EDA2­(ωB97M-V) and SAPT­(LRC-ωPBE) + MBD repulsion energies from 10.3 to 3.3 kcal/mol; for DFXC = HF, the discrepancy is reduced from 8.5 to 1.9 kcal/mol. Clearly, the antisymmetrized version of ALMO-EDA2 should not be compared side-by-side with SAPT.

Also in keeping with S66 results, the SAPT induction energies are about 2× smaller in magnitude than their ALMO-EDA2 counterparts. Linear regression affords slopes of 1.89 (def2-SVP) and 2.16 (def2-ma-TZVP), which translates into MADs of 0.9 and 1.5 kcal/mol, respectively, although some values are significantly larger, up to 6 kcal/mol. The two models for induction remain strongly correlated, with *R* ^2^ ≥ 0.97.

### 4.3 QUID Results in Detail

Having demonstrated that older, smaller data sets such as S22, S66, and L7 may not afford representative results for comparison of SAPT and ALMO-EDA energy components, we next present an in-depth discussion of results for the newer, larger QUID data set of biomolecular fragments. As in the analysis presented in Section 4.2, we use the ωB97M-V functional for the ALMO-EDA2 calculations. As discussed in Section 3.2.1, these SAPT + MBD calculations use fixed values of the range-separation parameter in LRC-ωPBE; see Table S5.

#### 4.3.1 Basis-Set Dependence

Figure 11a plots the basis-set dependence of the mean difference between total interaction energies and energy components computed using either EDA scheme. The largest source of basis-set dependence is the Pauli repulsion term, as we have previously observed for SAPT + MBD.^145^ The MAD between Δ*E*_Pauli_^mod^ and *E*_exch_^SAPT(KS)^ for the QUID dimers decreases from 4.3 kcal/mol (def2-SVP) to 2.3 kcal/mol (def2-ma-TZVP). Other energy components are only weakly dependent on the basis set.

**Figure 11:**
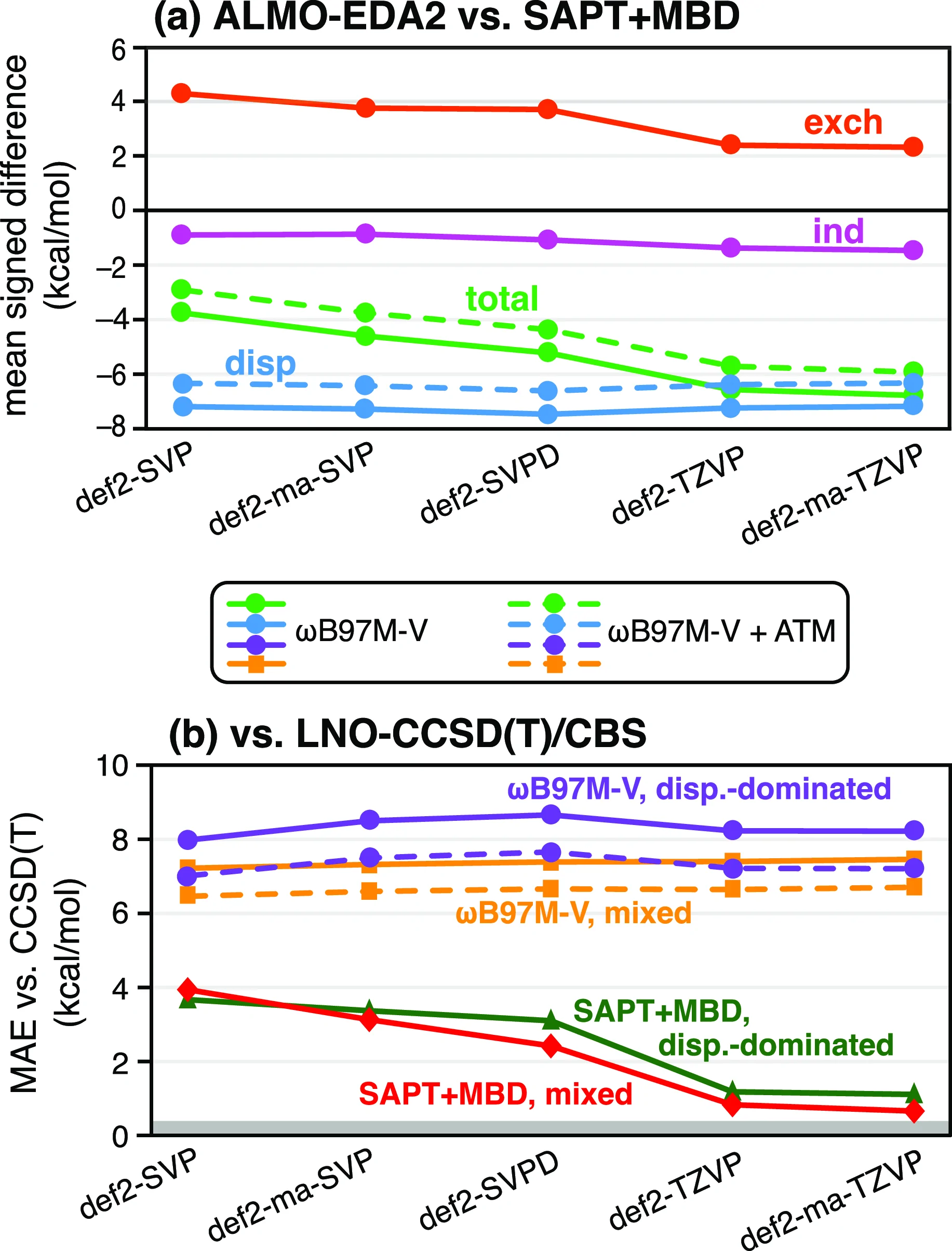
Basis-set convergence for interaction energies in the QUID data set. (a) Mean differences between ALMO-EDA2­(ωB97M-V) and SAPT­(LRC-ωPBE) + MBD energy components. The ALMO-EDA2 calculations employ the quasiclassical reference state and DFXC = dlDF, and are shown with (dashed lines) and without (solid lines) three-body ATM dispersion corrections that are not typically part of ωB97M-V. (b) MAEs in total interaction energies for either method, as compared to LNO-CCSD­(T)/CBS benchmarks from ref 91. The data set is partitioned into dispersion-dominated and mixed-influence dimers, with convergence shown separately for each subset. Again, ALMO-EDA2 results are shown both with and without ATM corrections. The shaded band near zero indicates the uncertainty of 0.39 kcal/mol that is reported for the benchmarks. SAPT­(LRC-ωPBE) calculations use fixed values of ω (Table S5).

The net result is that Pauli repulsion in ALMO-EDA2 is relatively more repulsive in small basis sets, which partially offsets a dispersion energy that is much more attractive but largely invariant to the choice of basis set. This partial compensation is reduced in larger basis sets, as the repulsion energy becomes relatively less so. Thus, agreement between ALMO-EDA2 and SAPT + MBD interaction energies deteriorates as the basis-set quality improves.

#### 4.3.2 Comparison to CCSD­(T)

Figure 11b plots mean absolute errors (MAEs) for the QUID dimers, relative to benchmarks computed at the LNO-CCSD­(T)/CBS level.^91^ Convergence plots as a function of basis set are separated into dispersion-dominated and mixed-influence dimers, according to criteria introduced in Section 3.1, although there is not much difference in the convergence behavior of the two subsets.

For SAPT­(LRC-ωPBE) + MBD, errors in *E*_int_ for the dispersion-dominated subset range from 3.7 kcal/mol (def2-SVP) to 1.1 kcal/mol (def2-ma-TZVP), whereas errors for the mixed-influence dimers range from 3.9 kcal/mol (def2-SVP) to 0.7 kcal/mol (def2-ma-TZVP). The latter value is close to the reported uncertainty in the benchmarks (0.4 kcal/mol).^91^ Thus, SAPT­(LRC-ωPBE) + MBD converges in the anticipated way with regard to basis set,^145^ and converged values are in reasonable agreement with the best available benchmarks.

The ωB97M-V interaction energies exhibit less basis-set dependence. This is not unexpected for noncovalent interactions,^144^ even if thermochemical calculations require triple-ζ basis sets.^149^ Even converged ωB97M-V/def2-ma-TZVP results, however, exhibit discrepancies of 7–8 kcal/mol with respect to the LNO-CCSD­(T)/CBS benchmarks. This is much larger than the errors exhibited by the SAPT­(LRC-ωPBE) + MBD/def2-ma-TZVP calculations.

One partial explanation for this discrepancy may be that the nonlocal VV10 functional,^150^ which comprises the “-V” contribution to ωB97M-V, is based on a two-body dispersion *ansatz*.^151^ Dispersion nonadditivity, defined here at the level of atoms, becomes important as monomer size increases.^98^ This is likely one reason why many dispersion-corrected and dispersion-inclusive DFT methods that agree very well for small dimers (such as those in S22 and S66) diverge from one another in complexes at the hundred-atom scale.^62^

The magnitude of the nonadditive dispersion effects that are missing in ωB97M-V calculations can be estimated by incorporating three-body dispersion corrections of the Axilrod–Teller–Muto (ATM) “triple-dipole” variety.^135^ Such corrections are a part of Grimme’s D3 and D4 dispersion models.^131,135^ Here, we extract the ATM contribution from the D4 model and add it to the dispersion energy computed using ALMO-EDA2­(ωB97M-V). ATM-corrected results, for both Δ*E*_disp_ and Δ*E*_int_, are plotted in Figure 11 alongside the original (uncorrected) ωB97M-V data.

The ATM corrections, which are mostly positive, average 0.8 kcal/mol per complex and 95% of them are smaller than 1.5 kcal/mol (Figure S19). This reduces the disparity with *E*_disp_^MBD^ by that amount (Figure S20) and moves the ωB97M-V interaction energies in the direction of the CCSD­(T) benchmarks. However, the disparity remains sizable and larger than the errors in SAPT + MBD interaction energies (Figure 11b). In future work, we may consider a more detailed examination of SAPT + MBD versus DFT for the QUID data set, as these dimers are large enough to demonstrate pronounced differences between the two approaches. For now, it appears that hybrid SAPT is fulfilling its promise as a systematically improvable means to perform EDA based on accurate interaction energies.^42–44^

#### 4.3.3 Influence of the δ_HF_ Correction

Adding the δ_HF_ correction to the SAPT induction energy in eq 5c affords

22 $$E_{ind}^{SAPT\left(KS\right)}=E_{ind}^{\left(20\right)}+E_{exch‐ind}^{\left(20\right)}+\delta_{HF}$$

The effect is to make SAPT induction more attractive. Typical changes for the QUID dimers are <1 kcal/mol although there are outliers where |δ_HF_| ranges up to 4 kcal/mol; see Figure 12. Basis-set effects on δ_HF_ are minimal, as we observed previously.^145^

**Figure 12:**
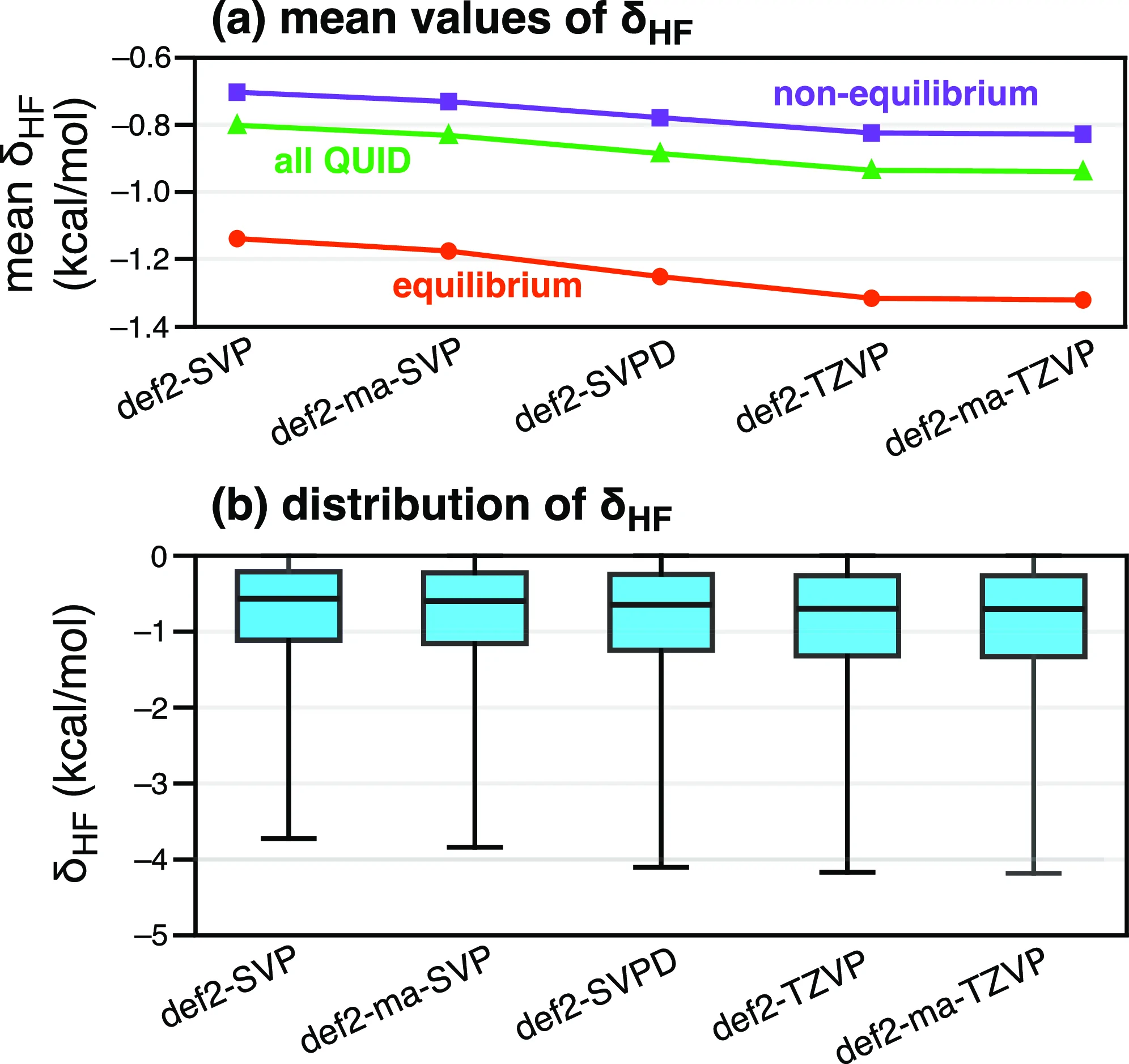
Summary of δ_HF_ corrections for the QUID data set. (a) Value of δ_HF_ averaged over all QUID dimers, as a function of basis set. Equilibrium (*R* = *R*_e_) and nonequilibrium structures have been separated for comparison. (b) Statistical summary of the δ_HF_ correction in each basis set, for all of the QUID dimers. Center lines in the box-and-whiskers plots indicate the median value and the boxes delineate the first and third quartiles. Whiskers span the minimum and maximum values of δ_HF_.

The δ_HF_ correction improves the agreement between SAPT­(LRC-ωPBE) + MBD induction energies and ALMO-EDA2­(ωB97M-V) polarization energies, reducing the linear regression slopes from 1.9 to 1.1 (def2-SVP) and from 2.2 to 1.3 (def2-ma-TZVP); see Figure 13. MADs between these induction/polarization energies are reduced to 0.25 kcal/mol (def2-SVP) and 0.53 kcal/mol (def2-ma-TZVP) when δ_HF_ is included. Thus, the factor-of-two disparity between ALMO-EDA2 polarization and SAPT + MBD induction (Figure 10) is an artifact of the second-order treatment of induction. This is not entirely surprising since ALMO polarization is variational and thus infinite-order in the same sense as the δ_HF_ correction.

**Figure 13:**
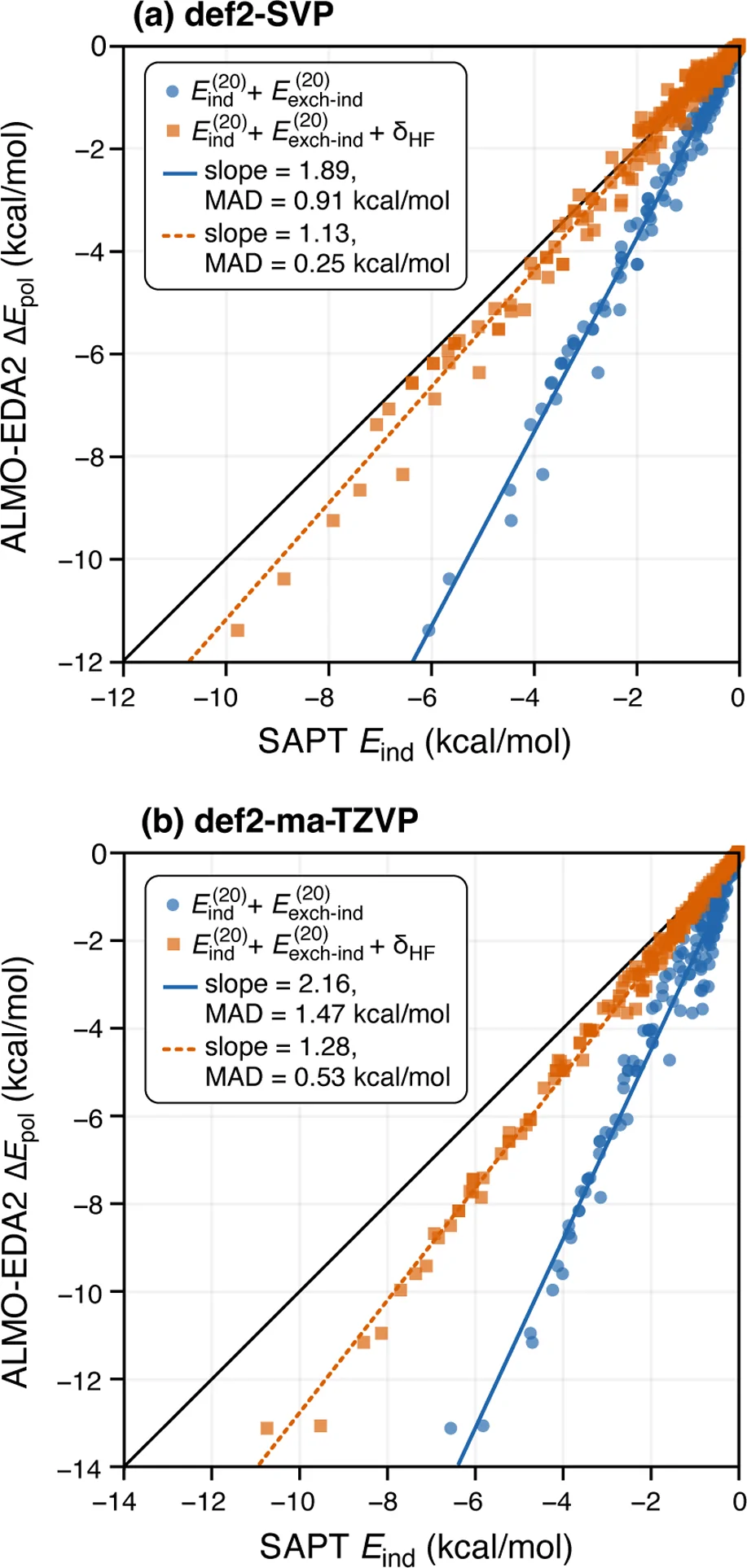
ALMO-EDA2­(ωB97M-V) polarization energies versus SAPT­(LRC-ωPBE) + MBD induction energies for the QUID data set, using (a) def2-SVP and (b) def2-ma-TZVP. SAPT values are shown with and without δ_HF_. (Values without δ_HF_ do not include response corrections.) Linear regressions and the *y* = *x* line are also plotted.

That said, inclusion of δ_HF_ actually degrades the agreement with CCSD­(T) benchmarks, as shown in Figure 14. These data again underscore that triple-ζ basis sets significantly improve agreement between SAPT + MBD and CCSD­(T), which is heartening since triple-ζ basis sets are necessary in order to converge SAPT + MBD energy components.^145^ Thus, improvement upon using def2-ma-TZVP looks like genuine physics rather than error cancellation. The ALMO-EDA2 results are less sensitive to the basis set, due to the use of counterpoise correction.^144^

**Figure 14:**
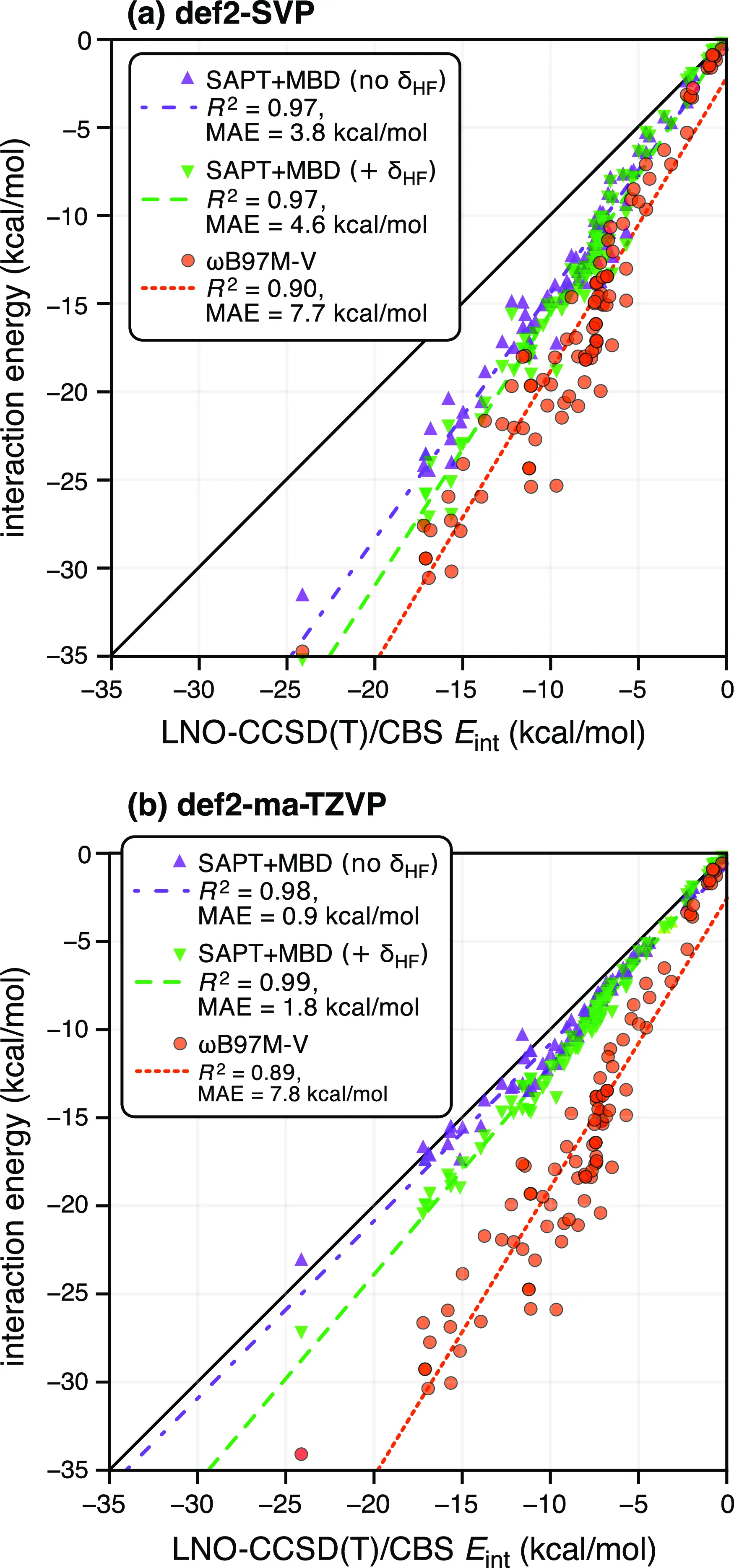
QUID interaction energies versus LNO-CCSD­(T)/CBS benchmarks. The vertical axis is the ωB97M-V or SAPT­(LRC-ωPBE) + MBD interaction energy computed using either (a) def2-SVP or (b) def2-ma-TZVP. SAPT interaction energies are shown with and without δ_HF_; the former do not include response corrections. Linear regressions and the *y* = *x* line are also shown.

While we have applied δ_HF_ universally in these tests, it has elsewhere been suggested that δ_HF_ should only be included in cases when polarization effects are large,^152^ as assessed using the ratio

23 $$\phi =\frac{\left|E_{ind}^{\left(20\right)}+E_{exch‐ind}^{\left(20\right)}\right|}{\left|E_{\mathrm{int}}\right|}$$

Empirically, it is suggested to include δ_HF_ only when ϕ > 0.125.^152^ This criterion is satisfied for only 28 (def2-SVP) or 58 (def2-ma-TZVP) of 186 data points in the QUID data set, including all of the dimers for which |*E*_ind_| > 3 kcal/mol (def2-SVP) or |*E*_ind_| > 2 kcal/mol (def2-ma-TZVP), as shown in Figure S21. For the def2-ma-TZVP basis set, turning on δ_HF_ only when ϕ > 0.125 improves the agreement with the CCSD­(T) benchmarks slightly but systematically (Figure S22), although the effect is much less pronounced in def2-SVP, where the agreement between SAPT­(LRC-ωPBE) + MBD and CCSD­(T) interaction energies is less good. With or without the δ_HF_ correction, the SAPT calculations are close to the benchmarks, more so when def2-ma-TZVP is used.

#### 4.3.4 Transferability Between EDAs

The QUID data set is intended to model protein–ligand interaction motifs, which is an important use case for noncovalent quantum chemistry and for EDA in particular.^153^ As such, quantitative comparisons between different EDA schemes are noteworthy and the analysis above has shown that strict transferability is only possible for electrostatics. Figure 15a attempts to summarize the transferability (or lack thereof) between SAPT + MBD and ALMO-EDA2 using the def2-ma-TZVP basis set. (For an analogous transferability map using def2-SVP, see Figure S23.) For this purpose we include a statistical summary of ALMO-EDA2 based on antisymmetrized electrostatics, in order to emphasize how dramatically that choice alters the comparison to SAPT electrostatics. Because that change does not alter total interaction energies, but simply repartitions the energy components, it must be offset somewhere else. Most of the difference is absorbed by the Pauli repulsion term. Meanwhile, a switch in the DFXC functional amounts to a small repartition between dispersion and Pauli repulsion (Figure 15b).

**Figure 15:**
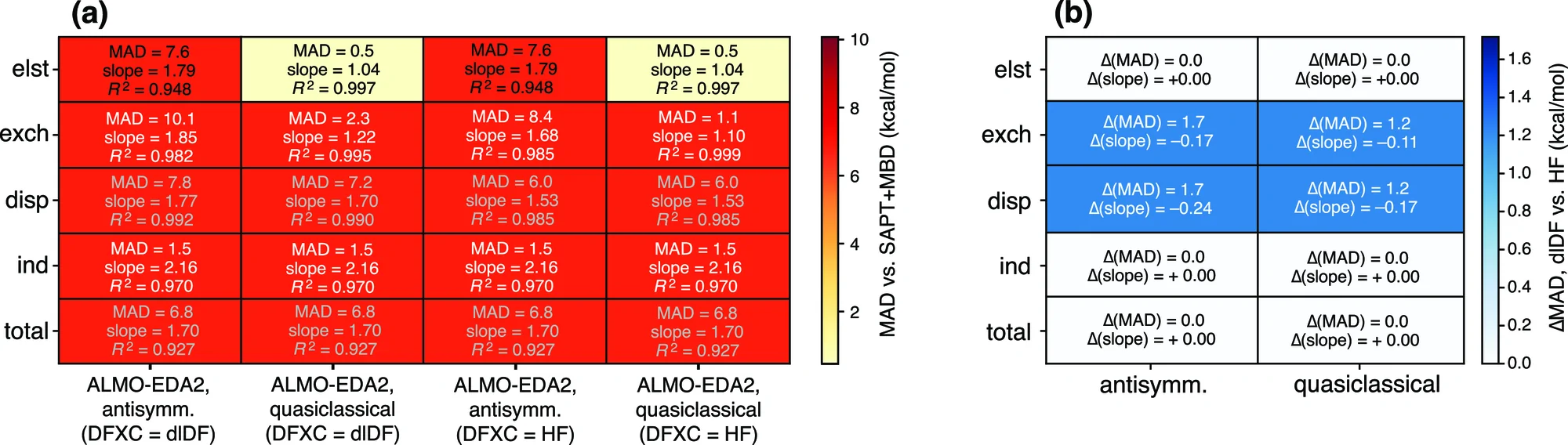
Transferability map for the QUID dimers. (a) Statistical comparison of SAPT­(LRC-ωPBE) + MBD to ALMO-EDA2­(ωB97M-V), using both the original (antisymmetrized electrostatics) and quasiclassical variants of ALMO-EDA2 with two different DFXC functionals. The summary includes MADs, linear regression slopes, and correlation coefficients *R* ^2^. (b) Change in the MADs and slopes when HF replaces dlDF as the DFXC functional in ALMO-EDA2. All calculations use the def2-ma-TZVP basis set. Gray, white, and black fonts are simply guides for the eye.

#### 4.3.5 Topology Dependence

The difference between DFXC functionals in the ALMO-EDA2 calculations is concentrated in the short-range regime, where overlap between fragment densities redistributes exchange repulsion and dispersion without changing the total interaction energy. The switch from dlDF to HF reduces the discrepancy in the Pauli repulsion energies, with respect to SAPT values, from 4.3 to 2.7 kcal/mol (def2-SVP) and from 2.3 to 1.1 kcal/mol (def2-ma-TZVP). The corresponding dispersion discrepancies change from 7.2 to 5.6 kcal/mol (def2-SVP), and from 7.2 to 6.0 kcal/mol (def2-ma-TZVP). The induction energy is rigorously unaffected by this switch, and the electrostatics energy is practically unchanged.

These observations motivate us to consider two related diagnostics. The first is the signed total interaction-energy difference

24 $$\Delta_{\mathrm{int}}=\Delta E_{\mathrm{int}}^{ALMO‐EDA2}-E_{\mathrm{int}}^{SAPT+MBD}$$

Mean values of Δ_int_ are plotted in Figure 16 for the three different topology classes contained within the QUID data set: folded, semifolded, and linear.^91^ Total interaction energies are systematically more attractive for ωB97M-V as compared to SAPT­(LRC-ωPBE) + MBD, in both the def2-SVP and def2-ma-TZVP basis sets, with the difference being largest for the folded subset where the interaction energies themselves are largest in magnitude. These results clearly demonstrate that def2-SVP calculations are at least 3–4 kcal/mol away from basis-set convergence.

**Figure 16:**
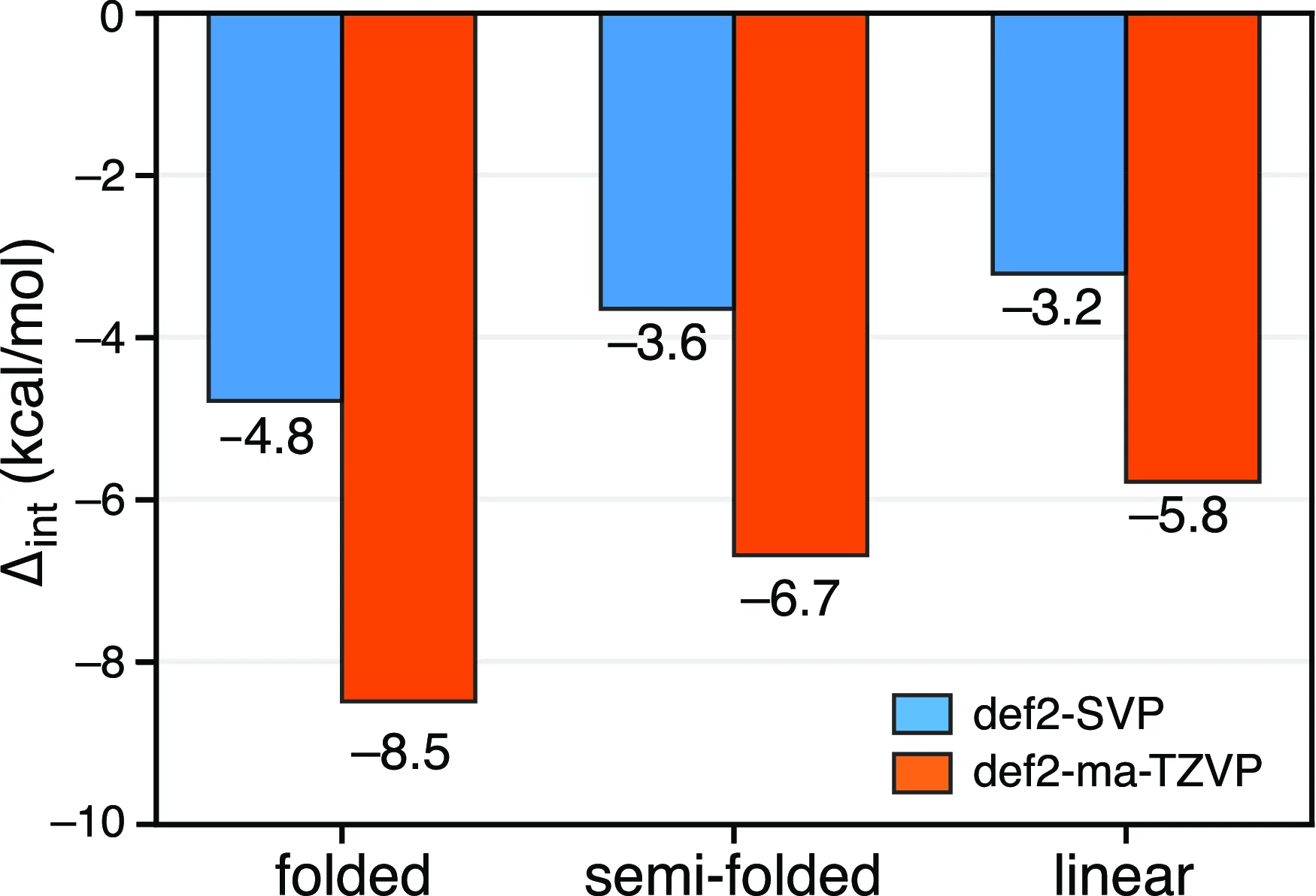
Mean values of Δ_int_ (eq 24) for the QUID dimers, grouped according to dimer topology.

Second, we define a metric to quantify the exchange/dispersion repartition engendered by switching the DFXC functional from HF to dlDF:

25 $$\begin{array}{ll} \Theta_{disp/rep} & =\Delta E_{Pauli}^{\mathrm{mod}}\left(dlDF\right)-\Delta E_{Pauli}^{\mathrm{mod}}\left(HF\right)\\ & =\Delta E_{disp}\left(HF\right)-\Delta E_{disp}\left(dlDF\right). \end{array}$$

This equation assumes that the quasiclassical ALMO-EDA2 reference state is employed. As such, the Pauli repulsion energies are Δ*E*_Pauli_^mod^ from eq 18.

Unsurprisingly, the repulsion-to-dispersion redistribution correlates with the magnitude of the Pauli repulsion itself, as shown in Figure 17. At the same time, the three topology classes have very different average values of Δ*E*_Pauli_^mod^, which scales with density overlap and is largest for the folded dimers. The mean value of Δ*E*_Pauli_^mod^ is 14.0 kcal/mol for the folded dimers, 10.8 kcal/mol for the semifolded complexes, and 9.0 kcal/mol for the linear geometries. Together with the correlation between |Θ_disp/rep_| and Δ*E*_Pauli_^mod^, this demonstrates that topology organizes the difference between EDA energy components.

**Figure 17:**
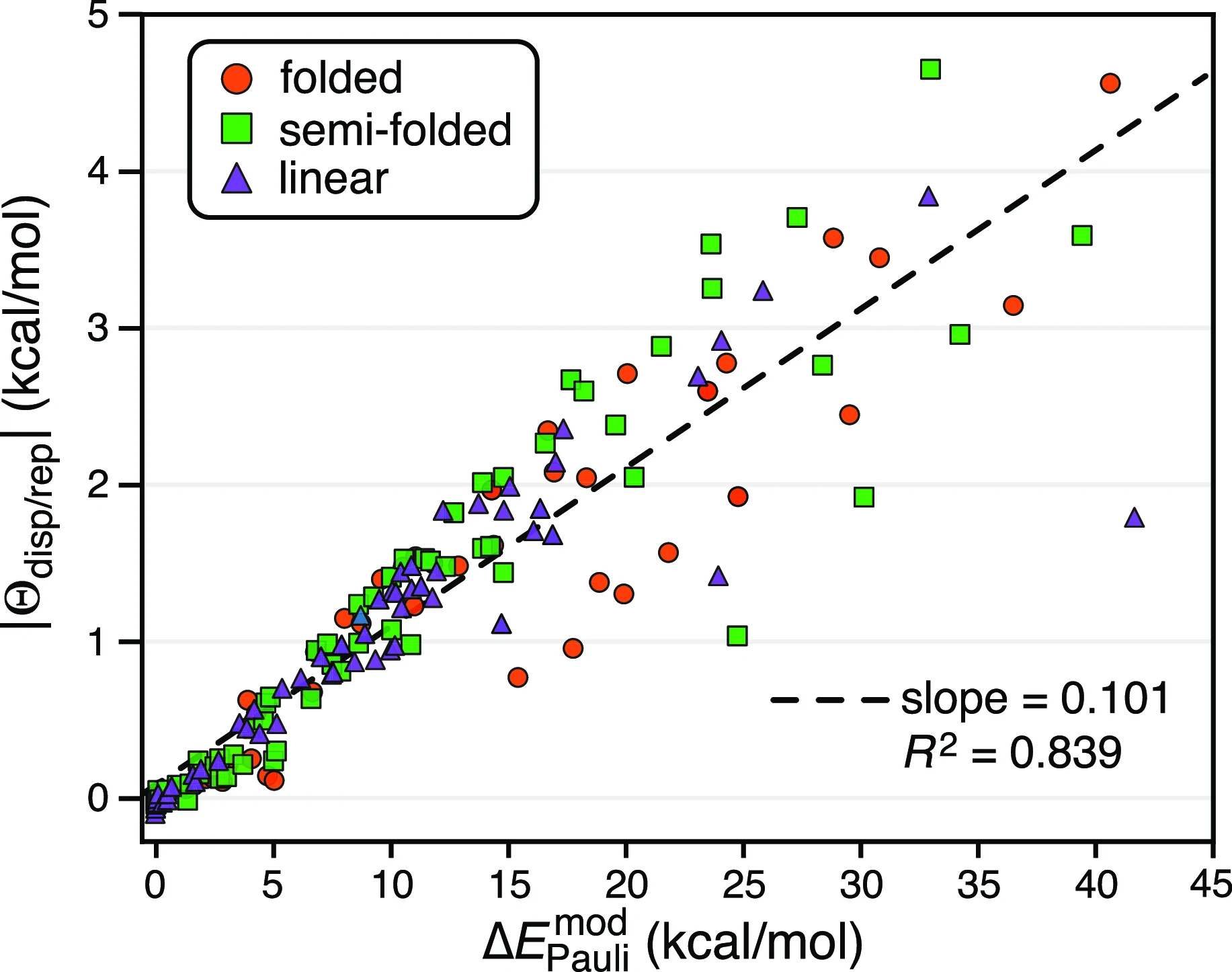
Redistribution |Θ_disp/rep_| (eq 25) plotted against the value of Δ*E*_Pauli_^mod^, for calculations at the ALMO-EDA2­(ωB97M-V)/def2-ma-TZVP level.

A scan along the intermolecular separation also helps to understand this repartitioning among the ALMO-EDA2 energy components. Figure 18 shows the average value of |Θ_disp/rep_| as a function of *q* = *R*/*R*_e_ for each of the three QUID topology classes. The quantity |Θ_disp/rep_| can be read as the average increase in Pauli repulsion or, equivalently, the average reduction in dispersion when dlDF is substituted for HF as the dispersion-free functional (*cf*. Table 4). The scan over *q* clearly indicates the short-range nature of this repartition. It also demonstrates that the numerical value of |Θ_disp/rep_| is largest for the folded dimers as they have the most close contacts, although the difference (relative to the semifolded and linear topologies) is slight compared to the rather strong distance dependence of |Θ_disp/rep_|. By *q* = 2.0, |Θ_disp/rep_| ≤ 0.1 kcal/mol for all three topology classes.

**Figure 18:**
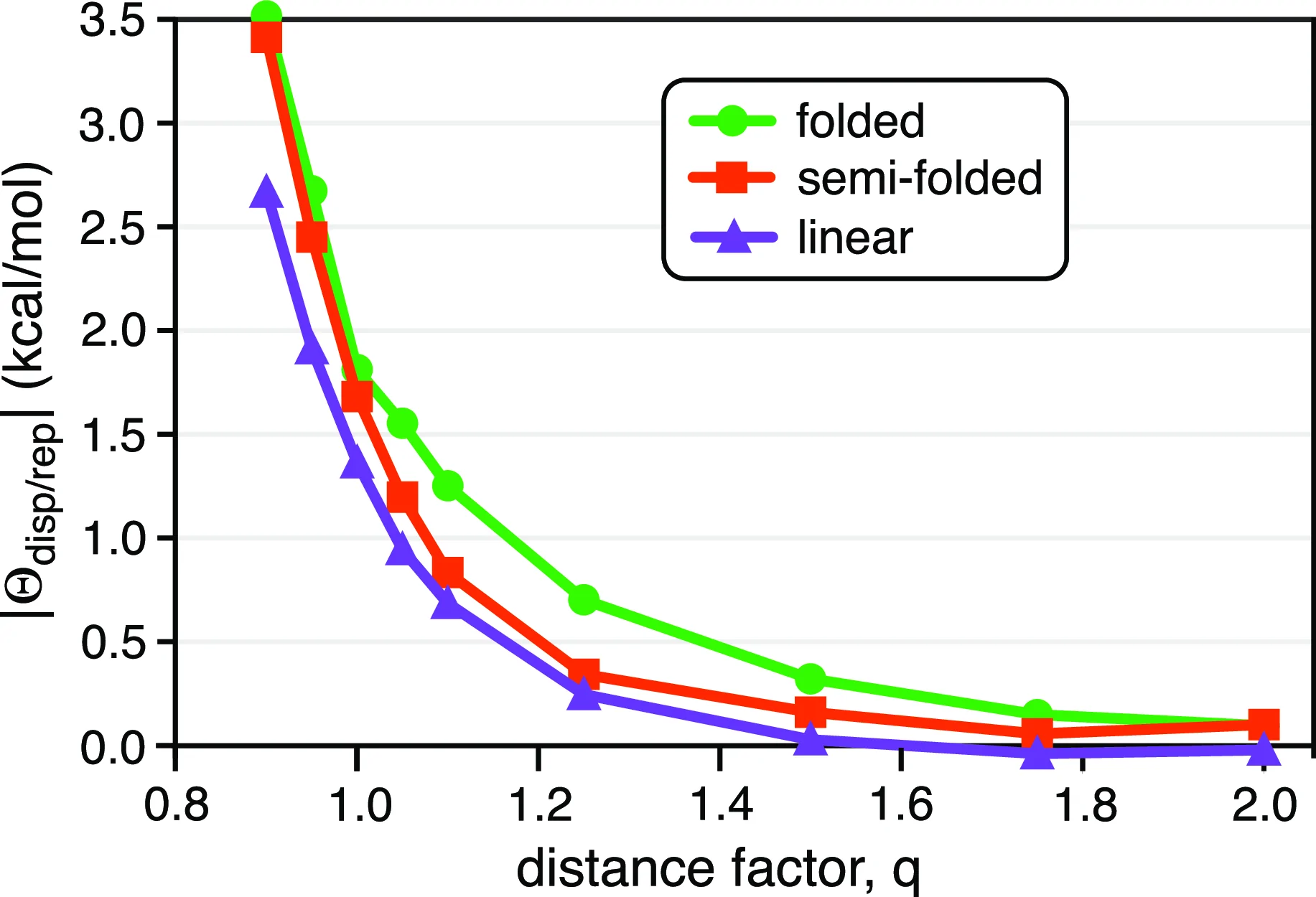
Distance-resolved exchange/dispersion repartition (|Θ_disp/rep_|, eq 25) evaluated for the QUID dimers using ALMO-EDA2­(ωB97M-V)/def2-ma-TZVP. Each data point is an average over all dimers in the indicated topology class (folded, semifolded, or linear) at a given value of the ratio *q* = *R*/*R*_e_.

## 5 Conclusions

The purpose of this study is not to recommend SAPT energy components over ALMO-EDA2­(DFT) values or *vice versa*. Rather, we aim to understand the extent to which energy components computed using one of these EDAs are correlated with those obtained from the other, and to establish calibration plots that allow for translation between the two schemes.

Results for the S22 data set suggest that the MBD model used in SAPT + MBD^40,41^ is a serviceable surrogate for third-order SAPT2+(3) dispersion. Taking MBD as the dispersion benchmark for the S66 data set, calculations in small dimers demonstrate that ALMO-EDA2­(DFT) dispersion energies are closer to MBD values when the dispersion-free dlDF functional is used to isolate Δ*E*_disp_ in the ALMO-EDA2­(DFT) calculations. This conclusion holds for a variety of target density functionals and might suggest using dlDF for best correspondence with SAPT calculations. However, the same pattern does not persist in larger van der Waals complexes.

We have also demonstrated that the use of an antisymmetrized reference state for ALMO-EDA2 can yield good statistical correlation—but poor numerical correspondence—with SAPT energy components. When a “quasiclassical” (direct-product) reference state is used instead, analogous to the usual choice for SAPT calculations, then both SAPT + MBD and ALMO-EDA2­(DFT) afford near-equivalent values for electrostatics. The same is not true for other energy components, however. Nevertheless, strong statistical correlations between ALMO-EDA2­(DFT) and SAPT + MBD energy components suggest that physical explanations (especially trends) that are gleaned from one of these EDA schemes are likely to be reproduced by the other. Previous work has demonstrated this explicitly in the case of π-stacking.^107^

Thus, the choice of EDA scheme may rest upon finding a method that affords accurate interaction energies. To that end, much of the present analysis has been performed using ωB97M-V, a state-of-the-art functional for noncovalent interactions. The accuracy of SAPT is improvable in principle, although most applications stick to second-order perturbation theory (SAPT0) for reasons of cost. In that case, a hybrid dispersion model is necessary in order to obtain accurate results.^37,38,42–44^ Comparison against CCSD­(T) benchmarks for dimers of biomolecular fragments suggests that SAPT­(LRC-ωPBE) + MBD outperforms ωB97M-V for total interaction energies. Use of triple-ζ basis sets improves the accuracy of SAPT + MBD although it is closer to the benchmarks already in double-ζ basis sets. Three-body dispersion corrections improve the ωB97M-V interaction energies by ∼1 kcal/mol with respect to CCSD­(T) benchmarks, but errors remain larger than those obtained using SAPT + MBD. Thus, SAPT + MBD emerges as a useful method for obtaining both accuracy and physical insight into noncovalent phenomena.

## Supplementary Material





## Data Availability

Calculations were performed using Q-Chem.^130^ A free trial license for Q-Chem can be obtained from https://www.q-chem.com/try, or else it can be accessed via the IQmol user interface that is available at https://www.iqmol.org. Sample Q-Chem input files are available in the Supporting Information. Raw data are available in CSV format as part of the Supporting Information.

## Notes

Additional figures and tables (PDF) Raw data (CSV, ZIP) (PDF)
(ZIP)

## §

Columbus State Community College, Delaware, Ohio 43015 USA
