# Supplementary material for: Comparing Symmetry-Adapted Perturbation Theory to Intermolecular Energy Decomposition Analysis Based on Absolutely Localized Molecular Orbitals
Source: J Chem Theory Comput. 2026 Oct 10;22(20):10700–20. doi: 10.1021/acs.jctc.6c01446 (PMC13718554; doi:10.1021/acs.jctc.6c01446)
Supplement: Supplementary file 1 [file ct6c01446_si_001.pdf]

Supporting Information for:

Comparing Symmetry-Adapted Perturbation Theory to Intermolecular Energy Decomposition Analysis Based on Absolutely Localized Molecular Orbitals

Narayanan Puthillathu,[1,2] Montgomery Gray,[1,†] and John M. Herbert[1,2,*]

[1] *Department of Chemistry & Biochemistry, The Ohio State University, Columbus, Ohio*
[2] *Biophysics Graduate Program, The Ohio State University, Columbus, Ohio*

October 8, 2026

List of Figures

S1 Ternary diagram indicating how the 186 QUID dimers cover the $(E_{\mathrm{elst}}, E_{\mathrm{exch}}, E_{\mathrm{disp}})$ space. Calculations were performed at the SAPT(LRC-ωPBE) + MBD/def2-ma-TZVP level and plotted according to fractions $|E_i|/(|E_{\mathrm{elst}}| + |E_{\mathrm{exch}}| + |E_{\mathrm{disp}}|)$ for $i \in \{\mathrm{elst}, \mathrm{exch}, \mathrm{disp}\}$. (The triangular interior grid denotes increments of 0.25 along each fractional component axis.) The dimers are characterized as electrostatics-dominated if $|E_{\mathrm{elst}}| \geq 2|E_{\mathrm{disp}}|$ and dispersion-dominated if $|E_{\mathrm{disp}}| \geq 2|E_{\mathrm{elst}}|$. Otherwise, they are classified as having mixed-influence interactions. S5

S2 Sample ALMO-EDA2 input file for $(H_2O)_2$ using Q-Chem v. 6.3, with B3LYP+D3(BJ) as the target functional and dlDF as the dispersion-free functional. The setting `EDA_CLS_DISP` = `TRUE` evaluates the monomer exchange-correlation terms in the dispersion expression using undistorted monomer densities. The dimer density remains antisymmetrized. The setting `EDA2` = 2 enables `EDA_CLS_ELEC`, which reports classical electrostatics from isolated monomer densities. This should correspond most closely to SAPT electrostatics in Eq. (10). S6

S3 Sample input file for GDD tuning on the H_2O molecule, using Q-Chem v. 6.3. The result is the value $\omega = 0.488$ bohr^{-1} that is used for H_2O in the `$lrc_omega` input section of Fig. S4. S6

S4 Sample input file for a SAPT(LRC-ωPBE) + MBD calculation on the dimer of N-methylacetamide and water, using Q-Chem v. 6.3. Range-separation parameters in the `$lrc_omega` input section were obtained by GDD tuning,[1] in a separate step that is illustrated for H_2O in Fig. S3. . . . . S7

S5 Sample Q-Chem v. 7.0 input file to extract the ATM (nonadditive dispersion) correction from a DFT+D4 calculation. The correction is geometry- but not density-dependent so the level of theory is not important. By setting the C_6 and C_8 scaling factors (s_6 and s_8, respectively) to zero, pairwise dispersion is turned off and the computed dispersion correction will be the three-body ATM part only. S8

† Present address: Columbus State Community College, Delaware, Ohio 43015 USA
* herbert@chemistry.ohio-state.edu

S6 Dispersion energies for the S22 dimers, correlating SAPT2+(3)/aug-cc-pVTZ values from Ref. 2 with ALMO-EDA2/def2-QZVPD values from the present work, using target functionals that include (a) ωB97M-V, (b) M06-2X, (c) B3LYP+D3(BJ), (d) BLYP+D3(BJ), (e) PBE0+D3(BJ), and (f) ωB97X-D. For each target functional, ALMO-EDA2 dispersion energies (using isolated-monomer densities as discussed in Section 2 .3 .2) are plotted using either HF or dlDF as the dispersion-free functional. Linear fits are indicated for either definition. A slope smaller than unity indicates that ALMO-EDA2 dispersion is less attractive than SAPT2+(3) dispersion, which is the case for all of the functionals when DFXC = HF. For DFXC = dlDF, the slopes are slightly larger and range from 0.91–1.01. Statistical differences and linear fit parameters are provided in Table S1. Panels (a) and (b) are the same as those in Fig. 2. . . . . S9
S7 MADs between the ALMO-EDA2(ωB97M-V) Pauli repulsion energy and the SAPT(LRC-ωPBE) + MBD exchange repulsion energy for the QUID data set. Two different reference states and DFXC functionals are compared. Statistics aggregate data from the def2-SVP and def2-ma-TZVP basis sets. S10
S8 Dispersion energies for the S66 dimers, correlating SAPT(LRC-ωPBE) + MBD to ALMO-EDA2 for target functionals that include (a) ωB97M-V, (b) M06-2X, (c) B3LYP+D3(BJ), (d) BLYP+D3(BJ), (e) PBE0+D3(BJ), and (f) ωB97X-D. In each case, we compare ALMO-EDA2 results using either dlDF or HF as the dispersion-free functional. All calculations use the def2-QZVPD basis set. Statistical differences and linear fit parameters are provided in Table 2. . . . . S11
S9 Dispersion energies for the L7 complexes from ALMO-EDA2 calculations using various target functionals, as indicated, with DFXC = HF in each case. SAPT(LRC-ωPBE) + MBD results are also shown. All calculations employ the def2-QZVPD basis set. S12
S10 Difference $\Delta_{\rm disp}$ (eq. 21) between MBD and ALMO-EDA2 dispersion energies for the L7 complexes, using either (a) DFXC = dlDF or (b) DFXC = HF in the ALMO-EDA2 calculations, for various target functionals. All calculations use the def2-QZVPD basis set. These are the same data that are plotted in Fig. 5, but here the individual complexes in L7 are shown. . . S13
S11 Summary of statistical differences between ALMO-EDA2 dispersion and $E_{\rm disp}$ obtained from (a) SAPT2+(3)/aug-cc-pVTZ, for the S22 data set; (b) SAPT(LRC-ωPBE) + MBD/def2-QZVPD, for the S66 data set; and (c) SAPT(LRC-ωPBE) + MBD/def2-QZVPD, for the L7 data set. All calculations use the def2-QZVPD basis set. For each horizontal line, corresponding a particular target functional, the two endpoints correspond to DFXC = dlDF (left endpoint) and DFXC = HF (right endpoint). Panels (a) and (b) plot MADs whereas panel (c) plots signed mean errors to show the change in sign. S14
S12 Dispersion energies for the QUID dimers, correlating SAPT(LRC-ωPBE) + MBD with ALMO-EDA2(ωB97M-V) dispersion for (a) def2-SVP and (b) def2-ma-TZVP. ALMO-EDA2 dispersion energies use either DFXC = HF or DFXC = dlDF, as indicated, and linear fits to either data set are shown. Panel (b) is the same as Fig. 6. . . . . S15
S13 Electrostatic interactions in the QUID data set, comparing results for two different basis sets. The four panels compare electrostatic energies from SAPT(LRC-ωPBE) + MBD and ALMO-EDA2(ωB97M-V) calculations, using (a) an antisymmetrized reference state with DFXC = dlDF, (b) a quasiclassical reference state with DFXC = dlDF, (c) an antisymmetrized reference state with DFXC = HF, and (d) a quasiclassical reference state with DFXC = HF. S16
S14 Pauli repulsion interactions in the QUID data set, comparing results for two different basis sets. The four panels compare $E_{\rm exch}$ from SAPT(LRC-ωPBE) + MBD to Pauli repulsion from ALMO-EDA2(ωB97M-V) using (a) an antisymmetrized reference state with DFXC = dlDF, (b) a quasiclassical reference state with DFXC = dlDF, (c) an antisymmetrized reference state with DFXC = HF, and (d) a quasiclassical reference state with DFXC = HF. S17

S15 Dispersion interactions in the QUID data set, comparing results for two different basis sets. The four panels compare $E_{\rm disp}$ from SAPT(LRC-ωPBE) + MBD to $\Delta E_{\rm disp}$ from ALMO-EDA2(ωB97M-V), using (a) an antisymmetrized reference state with DFXC = dlDF, (b) a quasiclassical reference state with DFXC = dlDF, (c) an antisymmetrized reference state with DFXC = HF, and (d) a quasiclassical reference state with DFXC = HF. S18
S16 Comparison of the induction energy from SAPT(LRC-ωPBE) + MBD with the polarization energy from ALMO-EDA2(ωB97M-V), for the QUID data set. Unlike the other energy components in Figs. S13–S15, $\Delta E_{\rm pol}$ does not depend on either the reference state (antisymmetrized or quasiclassical) or the choice of dispersion-free functional. S19
S17 Total interaction energies in the QUID data set, comparing SAPT(LRC-ωPBE) + MBD and ωB97M-V results in two different basis sets. S19
S18 Comparison of Pauli repulsion and dispersion energies for SAPT(LRC-ωPBE) + MBD versus ALMO-EDA(ωB97M-V) for the QUID data set, using different reference states (antisymmetrized or quasiclassical) and two different dispersion-free functionals (DFXC = dlDF or HF) for the ALMO-EDA2 calculations, as indicated. These panels contain the same data as in Figs. S14 and S15 but presented in a different way. S20
S19 Three-body ATM dispersion corrections for the QUID data set, sorted by topology categories. The middle bars are averages within each category and whiskers delineate minimum and maximum values. The ATM corrections are mostly positive (repulsive); three exceptions at large separation are indicated in red. S21
S20 Correlation between SAPT(LRC-ωPBE) + MBD dispersion and quasiclassical ALMO-EDA2(ωB97M-V) dispersion, comparing the latter both with and without three-body ATM. S21
S21 ALMO-EDA2(ωB97M-V) polarization energies versus SAPT(LRC-ωPBE) + MBD induction energies in either of two different basis sets. Results are shown for SAPT induction computed either with or without the $\delta_{\rm HF}$ correction, as in Fig. 13, and also for a procedure that adds $\delta_{\rm HF}$ only when $\phi > 0.125$ [see Eq. (23)]. The dashed line indicates $y = x$. S22
S22 SAPT(LRC-ωPBE) + MBD interaction energies in two different basis sets, as compared to LNO-CCSD(T)/CBS benchmarks. Results are shown for SAPT induction computed either with or without the $\delta_{\rm HF}$ correction, as in Fig. 14, and also for a procedure that adds $\delta_{\rm HF}$ only when $\phi > 0.125$ [see Eq. (23)]. The dashed line indicates $y = x$. S22
S23 Transferability map for the QUID dimers. (a) Statistical comparison of SAPT(LRC-ωPBE) + MBD to ALMO-EDA2(ωB97M-V), using both the original (antisymmetrized electrostatics) and quasiclassical electrostatics variants of the latter, with two different DFXC functionals. The statistical summary includes MADs, linear regression slopes, and correlation coefficients R^2. (b) Change in the MADs and slopes when HF replaces dlDF as the DFXC functional in the ALMO-EDA2 calculations. All calculations use the def2-ma-TZVP basis set. For a def2-SVP transferability map, see The corresponding def2-ma-TZVP version of these data appears in Fig. 15. . . . . S23

List of Tables

S1 Statistical differences between ALMO-EDA2 and SAPT2+(3) dispersion energies for the S22 dimers, including linear fit parameters.[a] S24
S2 Statistical differences between ALMO-EDA2 and SAPT2+(3) dispersion energies for the S22 dimers, calculated with distorted monomer densities.[a] S24
S3 Monomer-specific range-separation parameters employed in SAPT(LRC-ωPBE) + MBD calculations on the S66 data set, obtained using GDD tuning with the def2-QZVPD basis set. S25
S4 Monomer-specific range-separation parameters employed in SAPT(LRC-ωPBE) + MBD calculations on the L7 data set, obtained using GDD tuning with the def2-TZVPD basis set. S25

S5 Range separation parameters for the eleven QUID reference monomers comparing fixed values used in the original calculations with tuned values from the GDD procedure in two basis sets. S26
S6 Range-separation parameters ω and signed interaction energy differences for the QUID complexes in two different basis sets, comparing fixed versus GDD-tuned values of ω. S27

References

[1] Gray, M.; Herbert, J. M. Simplified tuning of long-range corrected density functionals for use in symmetry-adapted perturbation theory. *J. Chem. Phys.* **2021,** *155*, 034103.

[2] Flick, J. C.; Kosenkov, D.; Hohenstein, E. G.; Sherrill, C. D.; Slipchenko, L. V. Accurate prediction of noncovalent interaction energies with the effective fragment potential method: Comparison of energy components to symmetry-adapted perturbation theory for the S22 test set. *J. Chem. Theory Comput.* **2012,** *8*, 2835–2843.

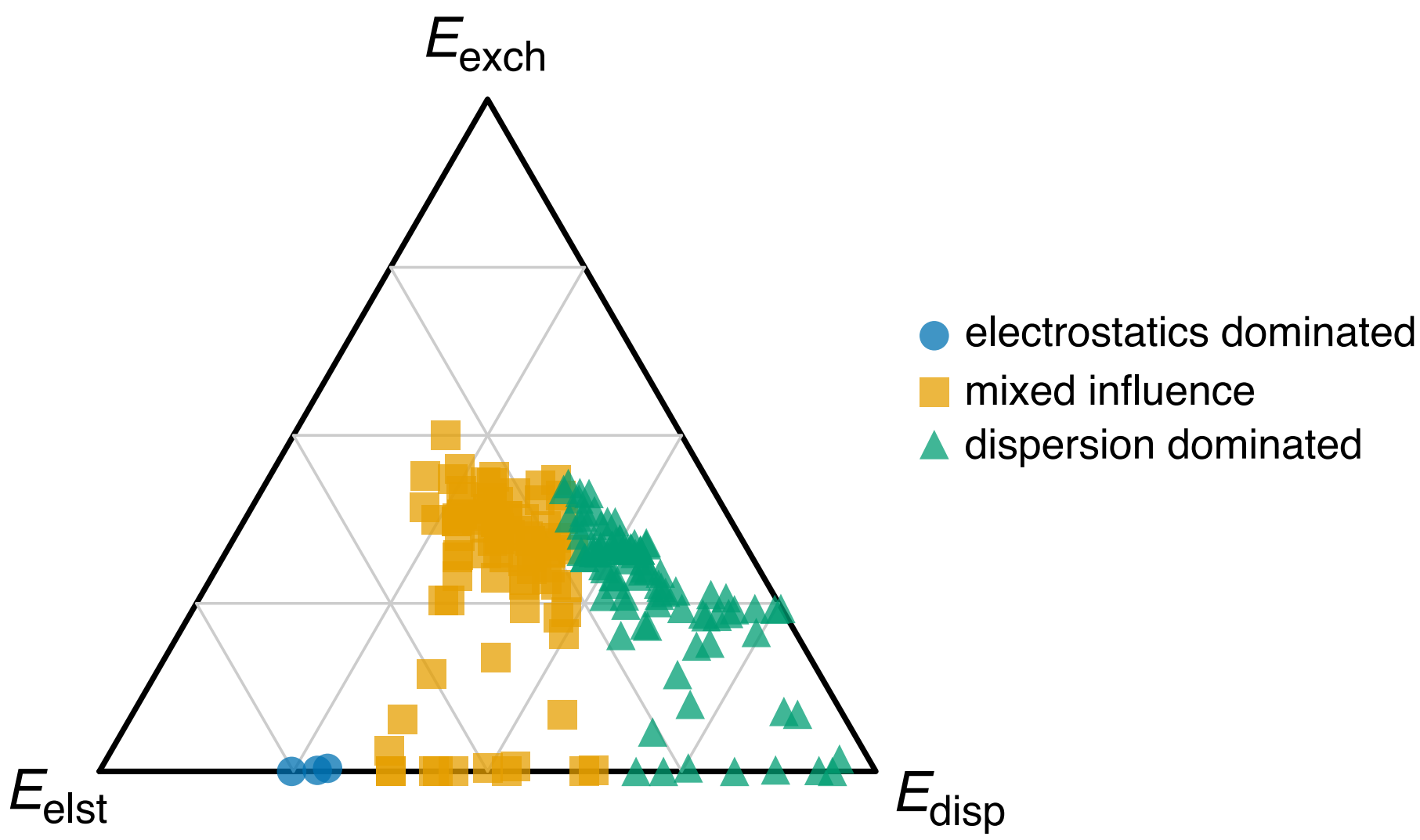

Fig. S1: Ternary diagram indicating how the 186 QUID dimers cover the $(E_{\rm elst}, E_{\rm exch}, E_{\rm disp})$ space. Calculations were performed at the SAPT(LRC-ωPBE) + MBD/def2-ma-TZVP level and plotted according to fractions $|E_i|/(|E_{\rm elst}| + |E_{\rm exch}| + |E_{\rm disp}|)$ for $i \in \{\rm elst, exch, disp\}$. (The triangular interior grid denotes increments of 0.25 along each fractional component axis.) The dimers are characterized as electrostatics-dominated if $|E_{\rm elst}| \geq 2|E_{\rm disp}|$ and dispersion-dominated if $|E_{\rm disp}| \geq 2|E_{\rm elst}|$. Otherwise, they are classified as having mixed-influence interactions.

```
$molecule
0 1
--
0 1
  O   -0.702196054  -0.056060256   0.009942262
  H   -1.022193224   0.846775782  -0.011488714
  H    0.257521062   0.042121496   0.005218999
--
0 1
  O    2.220871067   0.026716792   0.000620476
  H    2.597492682  -0.411663274   0.766744858
  H    2.593135384  -0.449496183  -0.744782026
$end

$rem
  JOBTYPE      EDA
  EDA_CLS_DISP TRUE   ! whether to use classical dispersion
  EDA2         2
  METHOD       B3LYP
  DFT_D        D3_BJ
  BASIS        def2-QZVPD
  DISP_FREE_C  NONE
  DISP_FREE_X  dlDF   ! alternatively, could be set to HF
  EDA_BSSE     TRUE   ! remove BSSE from the CT term
  THRESH                 14
  SCF_CONVERGENCE        8
  FRZ_ORTHO_DECOMP_CONV  8
$end
```

Fig. S2: Sample ALMO-EDA2 input file for $(H_2O)_2$ using Q-Chem v. 6.3, with B3LYP+D3(BJ) as the target functional and dlDF as the dispersion-free functional. The setting `EDA_CLS_DISP` = `TRUE` evaluates the monomer exchange-correlation terms in the dispersion expression using undistorted monomer densities. The dimer density remains antisymmetrized. The setting `EDA2` = `2` enables `EDA_CLS_ELEC`, which reports classical electrostatics from isolated monomer densities. This should correspond most closely to SAPT electrostatics in Eq. (10).

```
$molecule
0 1
  O        -0.393279494201    0.003953021781    0.000000000000
  H         0.189026010864   -0.758658480198    0.000000000000
  H         0.204253483337    0.754705458417    0.000000000000
$end

$rem
  METHOD     LRC-WPBE
  BASIS      DEF2-QZVPD
  OMEGA      300
  OMEGA_GDD  TRUE
  SCF_CONVERGENCE 8
  THRESH          14
$end
```

Fig. S3: Sample input file for GDD tuning on the H_2O molecule, using Q-Chem v. 6.3. The result is the value $\omega = 0.488$ bohr^{-1} that is used for H_2O in the `$lrc_omega` input section of Fig. S4.

```
$molecule
0 1
--
0 1
  C         0.515412733288   -0.473460405067    1.756757393235
  H         1.575035656996   -0.398467830102    1.526071885281
  H         0.288020716047   -1.480514194147    2.098540996396
  H         0.270089531224    0.209395869031    2.566654908928
  C        -0.375217632919   -0.155953832138    0.576958503198
  O        -1.597606497592   -0.183546666900    0.667022309200
  N         0.286297004241    0.155070994674   -0.567930261545
  H         1.293525977450    0.155908910863   -0.563750686813
  C        -0.418406599686    0.481569001856   -1.785663524799
  H        -1.480654596566    0.408419858701   -1.573763163570
  H        -0.190587557573    1.494438050957   -2.115204309627
  H        -0.165908734698   -0.212859757941   -2.585694049672
--
0 1
  O        -0.393279494201    0.003953021781    0.000000000000
  H         0.189026010864   -0.758658480198    0.000000000000
  H         0.204253483337    0.754705458417    0.000000000000
$end

$rem
  JOBTYPE     XSAPT
  METHOD      LRC-wPBE
  BASIS       def2-QZVPD
  LRC_DFT     TRUE
  SCF_GUESS   AUTOSAD
  THRESH         14
  SCF_CONVERGENCE 8
$end

$xpol
  EMBED NONE
  DFT-LRC
  PRINT 3
$end

$sapt
  PRINT 3
  ORDER 2
  ALGORITHM  AO
  BASIS      PROJECTED
  DISPERSION MBD
$end

$lrc_omega
  348
  488
$end
```

Fig. S4: Sample input file for a SAPT(LRC-ωPBE) + MBD calculation on the dimer of N-methylacetamide and water, using Q-Chem v. 6.3. Range-separation parameters in the `$lrc_omega` input section were obtained by GDD tuning,[1] in a separate step that is illustrated for H_2O in Fig. S3.

```
$molecule
0 1
--
0 1
  O   -0.702196054  -0.056060256   0.009942262
  H   -1.022193224   0.846775782  -0.011488714
  H    0.257521062   0.042121496   0.005218999
--
0 1
  O    2.220871067   0.026716792   0.000620476
  H    2.597492682  -0.411663274   0.766744858
  H    2.593135384  -0.449496183  -0.744782026
$end

$rem
  METHOD HF
  BASIS  STO-3G
  DFT_D  D4
  DFT_D4_S6 0  ! set s_6 = 0
  DFT_D4_S8 0  ! set s_8 = 0
  DFT_D4_S9 100000000
  DFT_D4_A1 6620000
  DFT_D4_A2 544870000
  SYM_IGNORE TRUE
$end
```

Fig. S5: Sample Q-Chem v. 7.0 input file to extract the ATM (nonadditive dispersion) correction from a DFT+D4 calculation. The correction is geometry- but not density-dependent so the level of theory is not important. By setting the C_6 and C_8 scaling factors (s_6 and s_8, respectively) to zero, pairwise dispersion is turned off and the computed dispersion correction will be the three-body ATM part only.

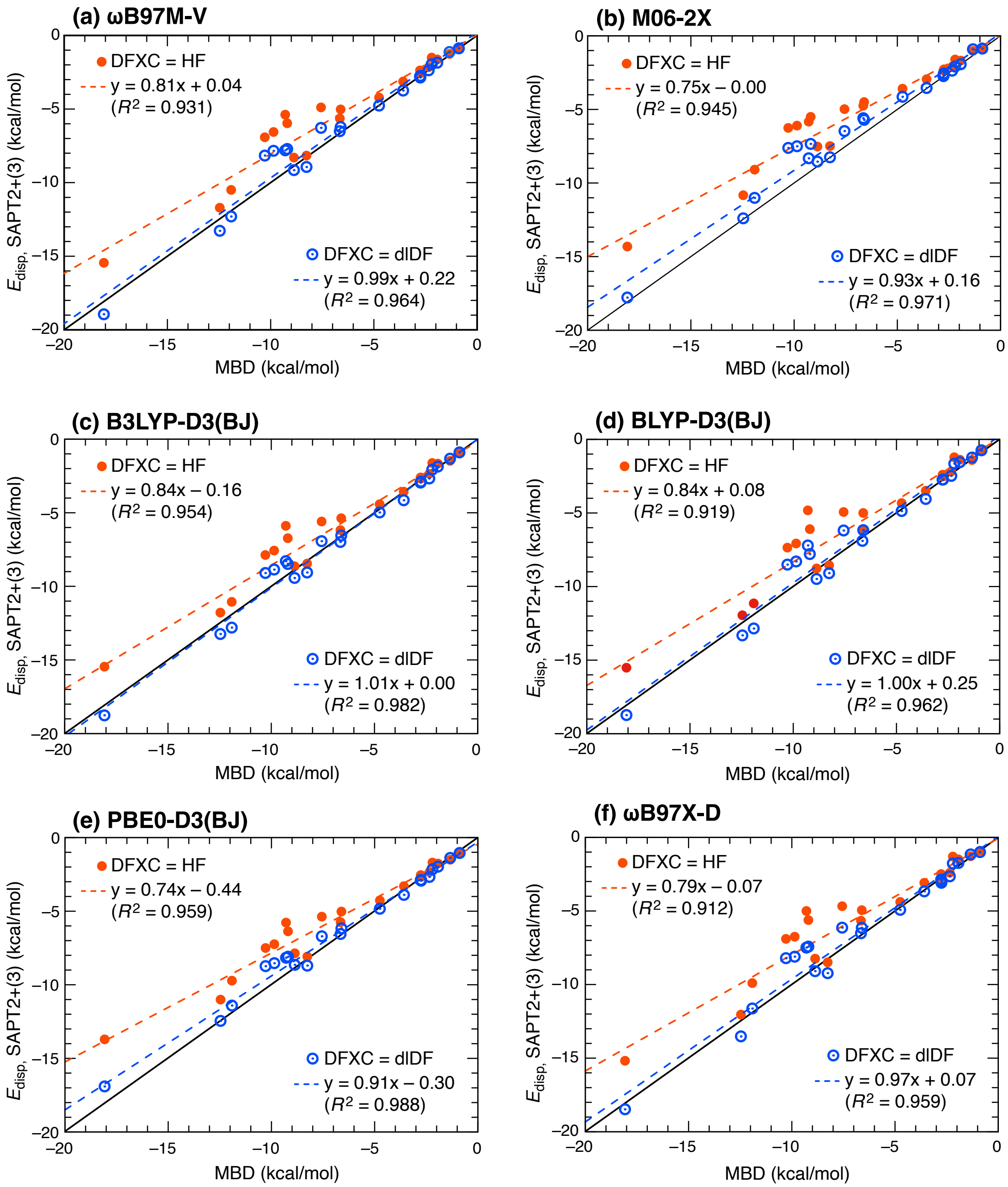

Fig. S6: Dispersion energies for the S22 dimers, correlating SAPT2+(3)/aug-cc-pVTZ values from Ref. 2 with ALMO-EDA2/def2-QZVPD values from the present work, using target functionals that include (a) ωB97M-V, (b) M06-2X, (c) B3LYP+D3(BJ), (d) BLYP+D3(BJ), (e) PBE0+D3(BJ), and (f) ωB97X-D. For each target functional, ALMO-EDA2 dispersion energies (using isolated-monomer densities as discussed in Section 2.3.2) are plotted using either HF or dlDF as the dispersion-free functional. Linear fits are indicated for either definition. A slope smaller than unity indicates that ALMO-EDA2 dispersion is less attractive than SAPT2+(3) dispersion, which is the case for all of the functionals when DFXC = HF. For DFXC = dlDF, the slopes are slightly larger and range from 0.91–1.01. Statistical differences and linear fit parameters are provided in Table S1. Panels (a) and (b) are the same as those in Fig. 2.

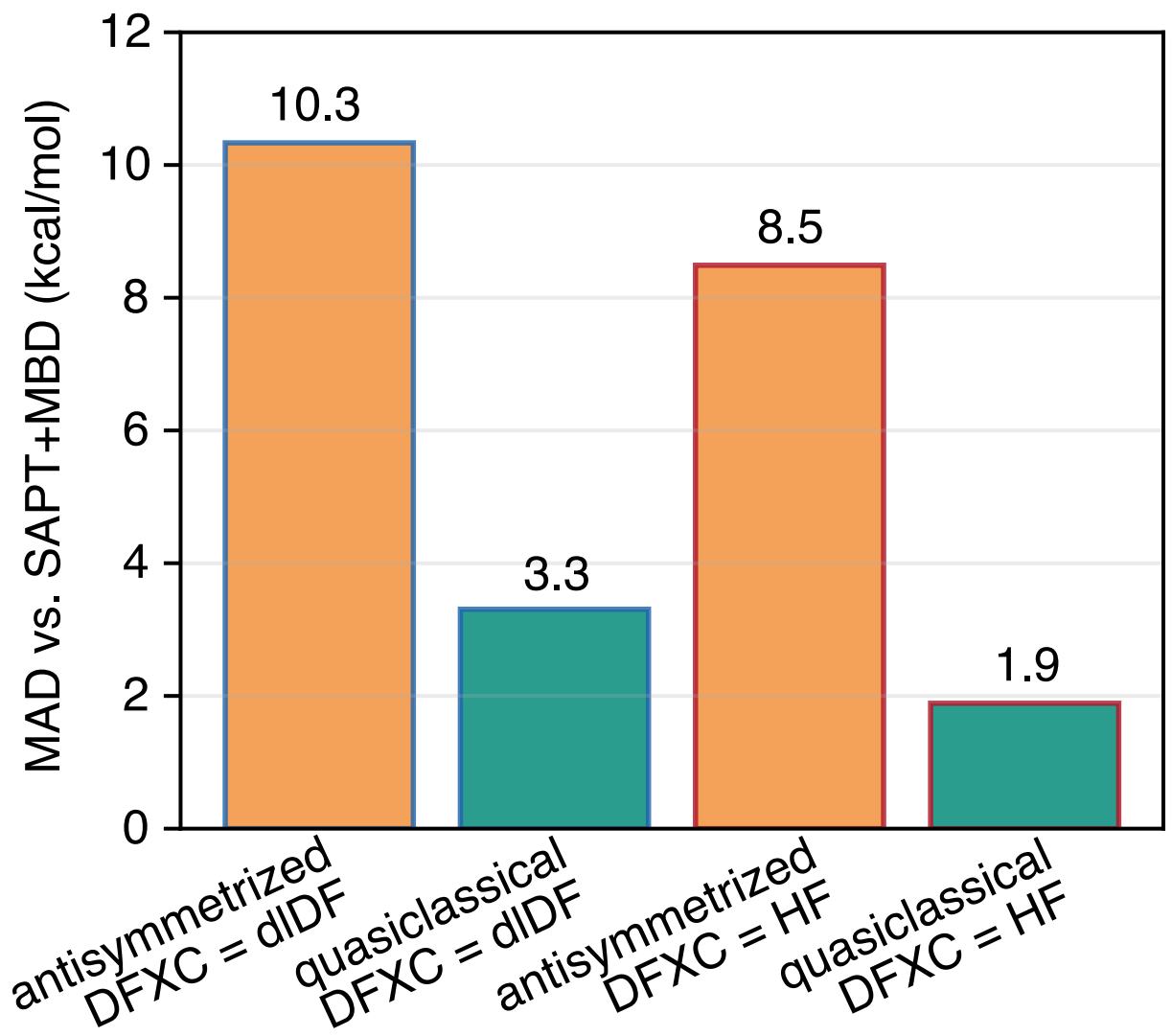

Fig. S7: MADs between the ALMO-EDA2(ωB97M-V) Pauli repulsion energy and the SAPT(LRC-ωPBE) + MBD exchange repulsion energy for the QUID data set. Two different reference states and DFXC functionals are compared. Statistics aggregate data from the def2-SVP and def2-ma-TZVP basis sets.

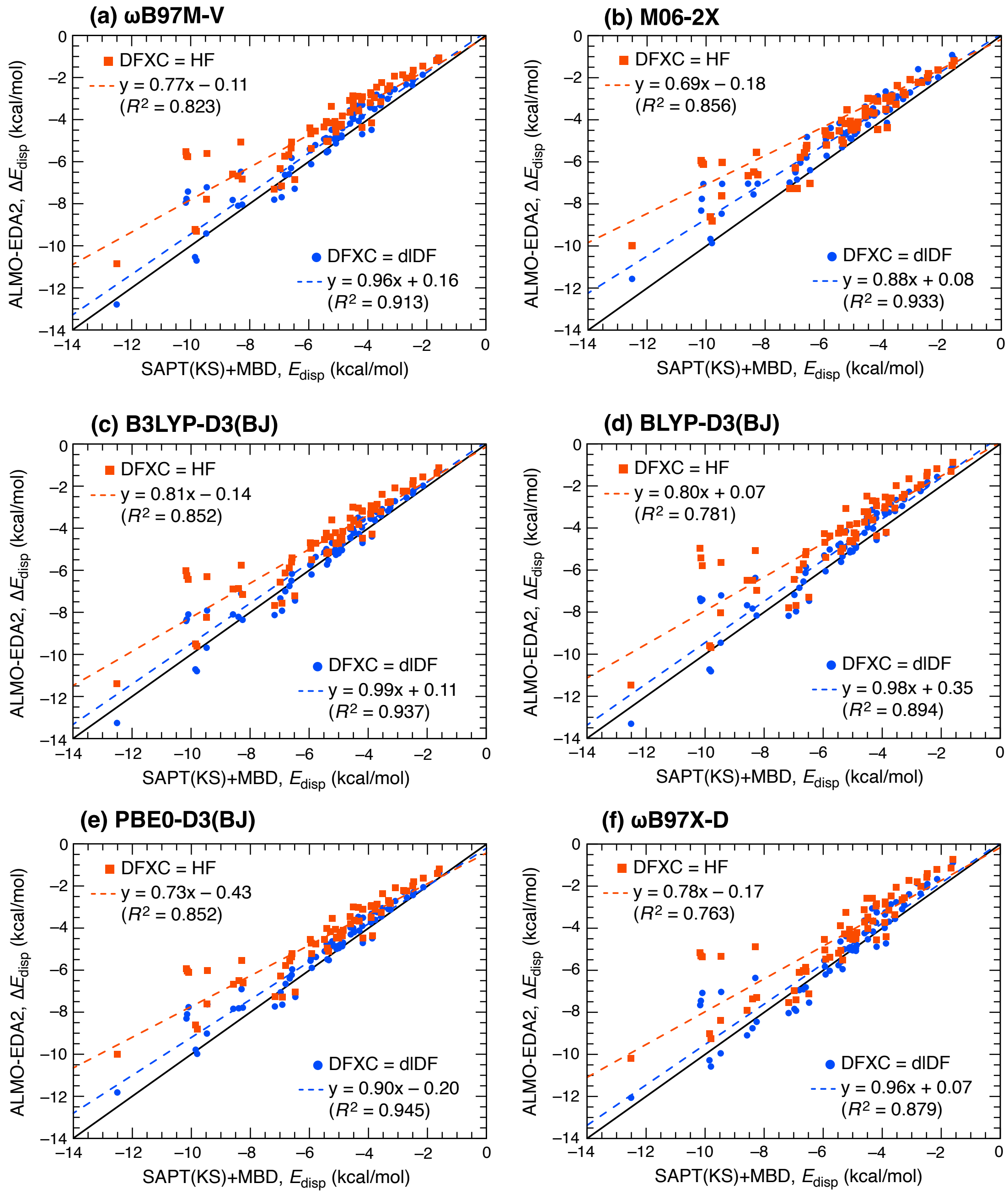

Fig. S8: Dispersion energies for the S66 dimers, correlating SAPT(LRC-ωPBE) + MBD to ALMO-EDA2 for target functionals that include (a) ωB97M-V, (b) M06-2X, (c) B3LYP+D3(BJ), (d) BLYP+D3(BJ), (e) PBE0+D3(BJ), and (f) ωB97X-D. In each case, we compare ALMO-EDA2 results using either dlDF or HF as the dispersion-free functional. All calculations use the def2-QZVPD basis set. Statistical differences and linear fit parameters are provided in Table 2.

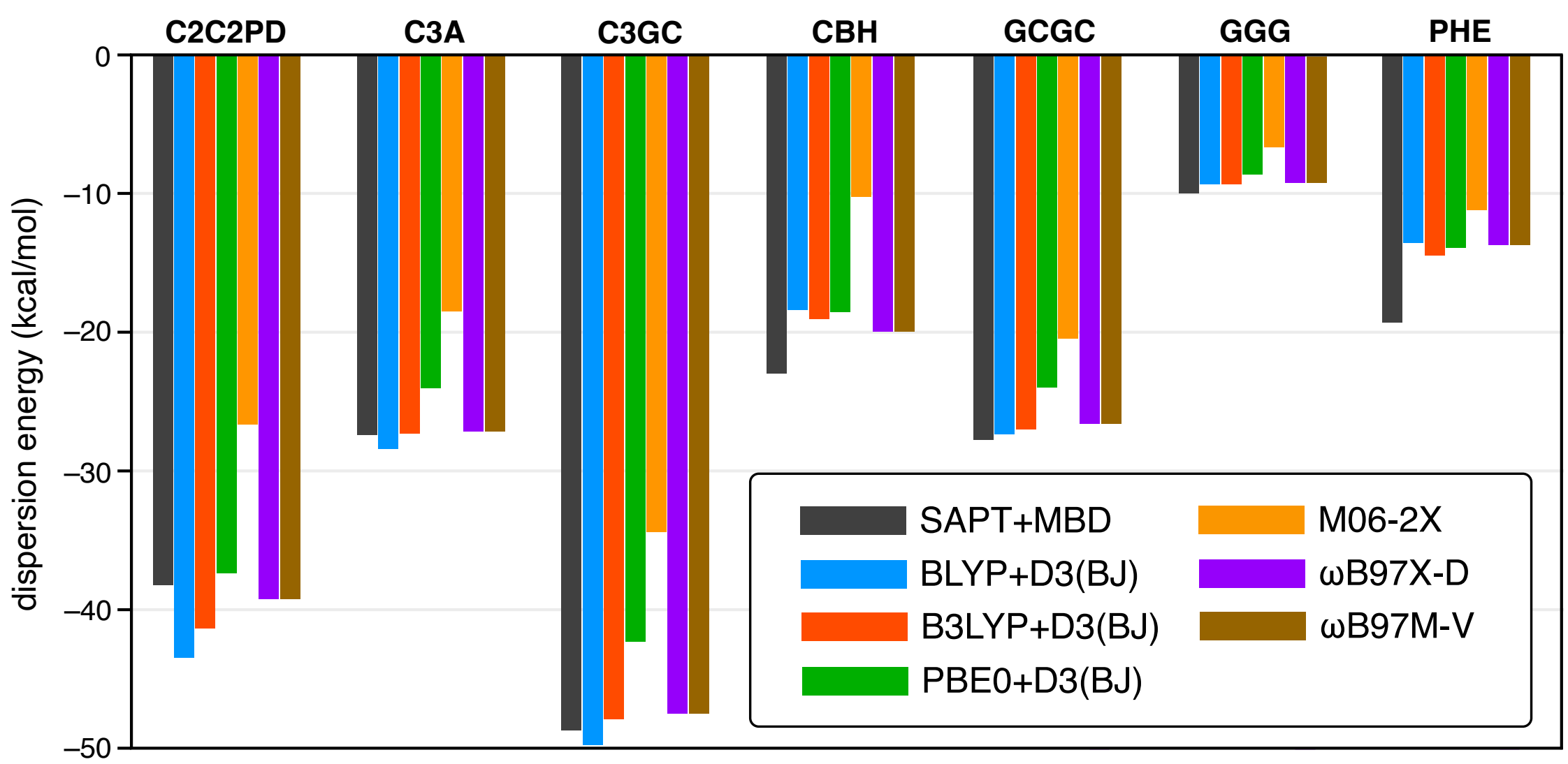

Fig. S9: Dispersion energies for the L7 complexes from ALMO-EDA2 calculations using various target functionals, as indicated, with DFXC = HF in each case. SAPT(LRC-ωPBE) + MBD results are also shown. All calculations employ the def2-QZVPD basis set.

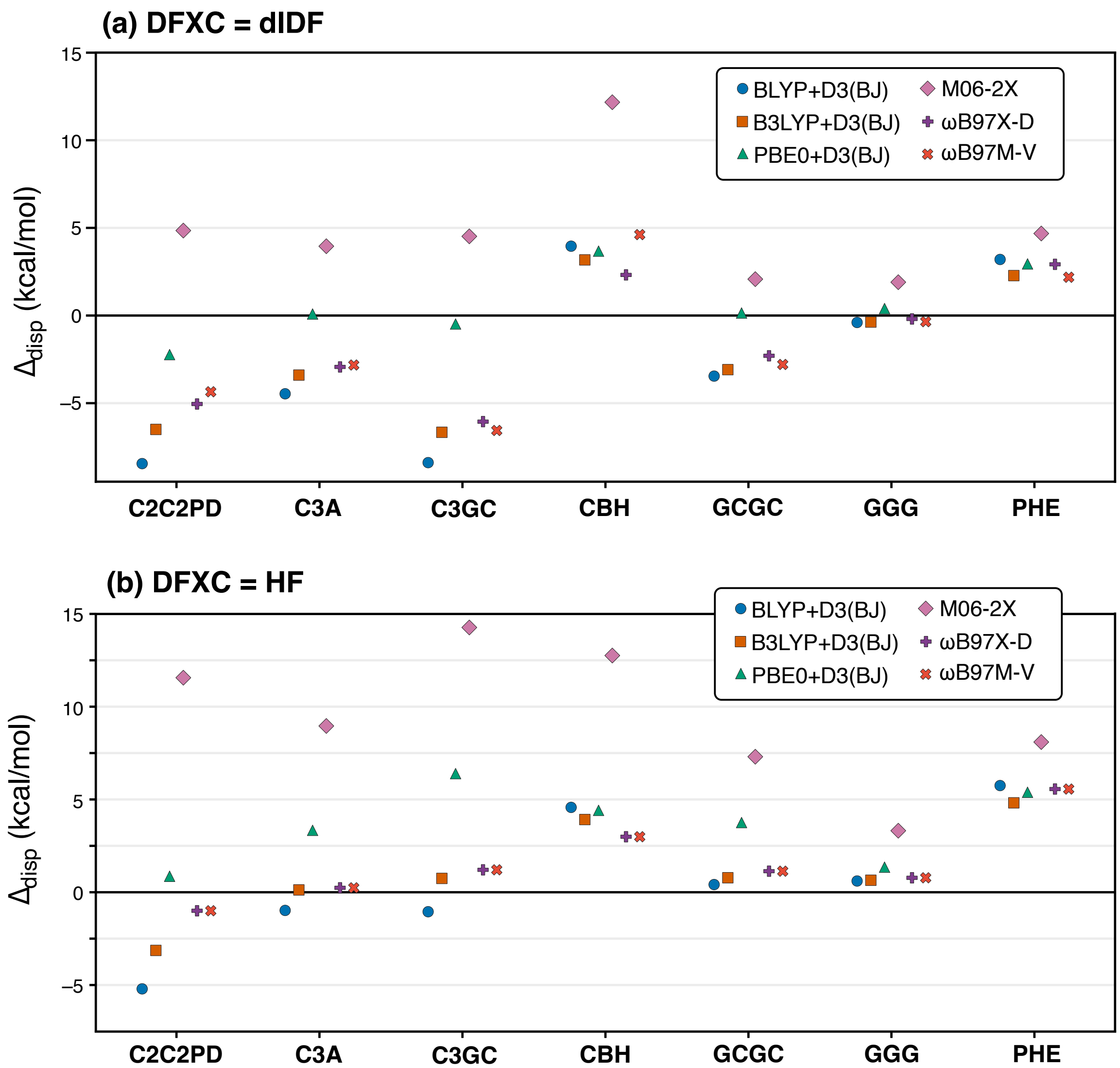

Fig. S10: Difference $\Delta_{\rm disp}$ (eq. 21) between MBD and ALMO-EDA2 dispersion energies for the L7 complexes, using either (a) DFXC = dlDF or (b) DFXC = HF in the ALMO-EDA2 calculations, for various target functionals. All calculations use the def2-QZVPD basis set. These are the same data that are plotted in Fig. 5, but here the individual complexes in L7 are shown.

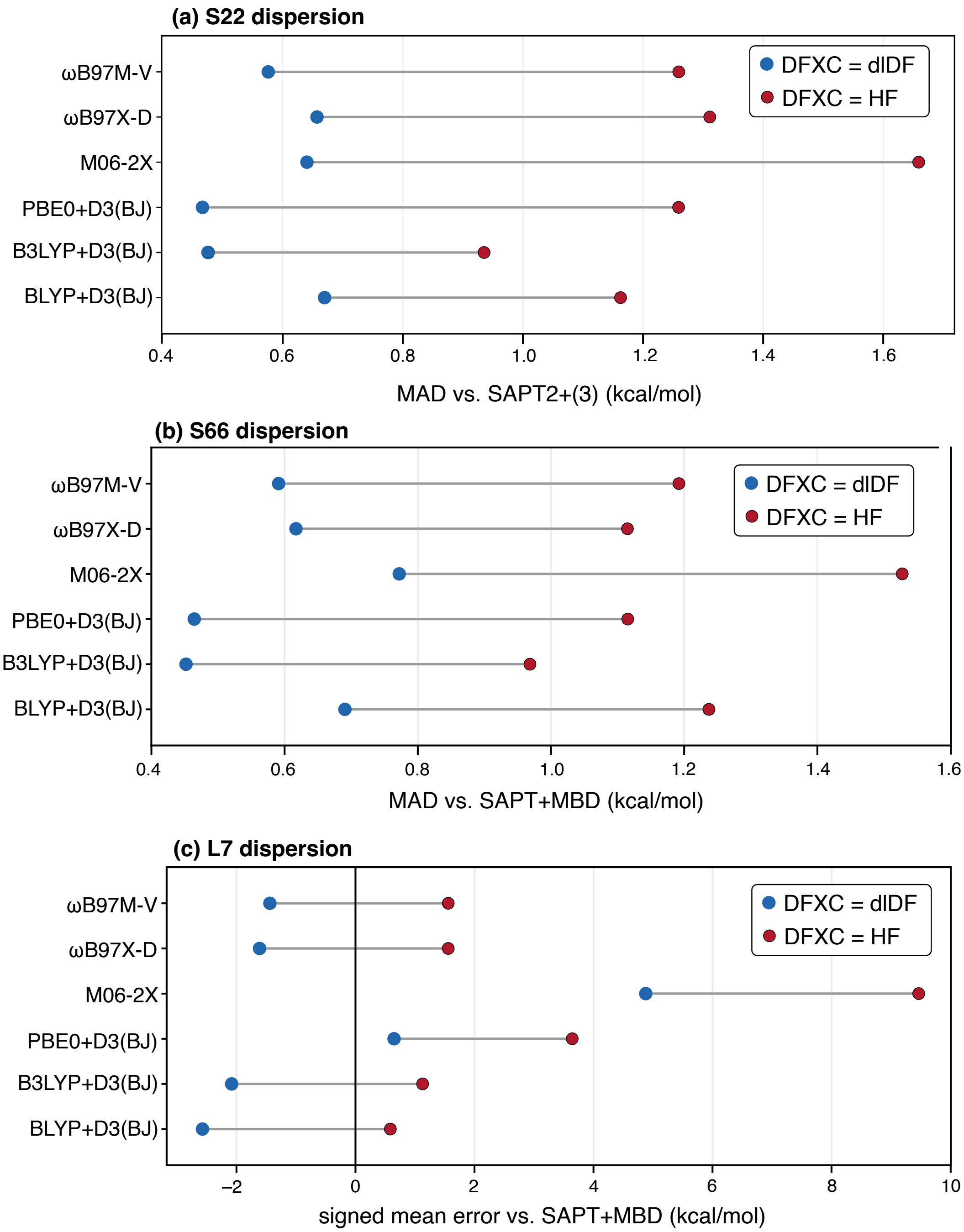

Fig. S11: Summary of statistical differences between ALMO-EDA2 dispersion and E_{disp} obtained from (a) SAPT2+(3)/aug-cc-pVTZ, for the S22 data set; (b) SAPT(LRC-ωPBE) + MBD/def2-QZVPD, for the S66 data set; and (c) SAPT(LRC-ωPBE) + MBD/def2-QZVPD, for the L7 data set. All calculations use the def2-QZVPD basis set. For each horizontal line, corresponding a particular target functional, the two endpoints correspond to DFXC = dlDF (left endpoint) and DFXC = HF (right endpoint). Panels (a) and (b) plot MADs whereas panel (c) plots signed mean errors to show the change in sign.

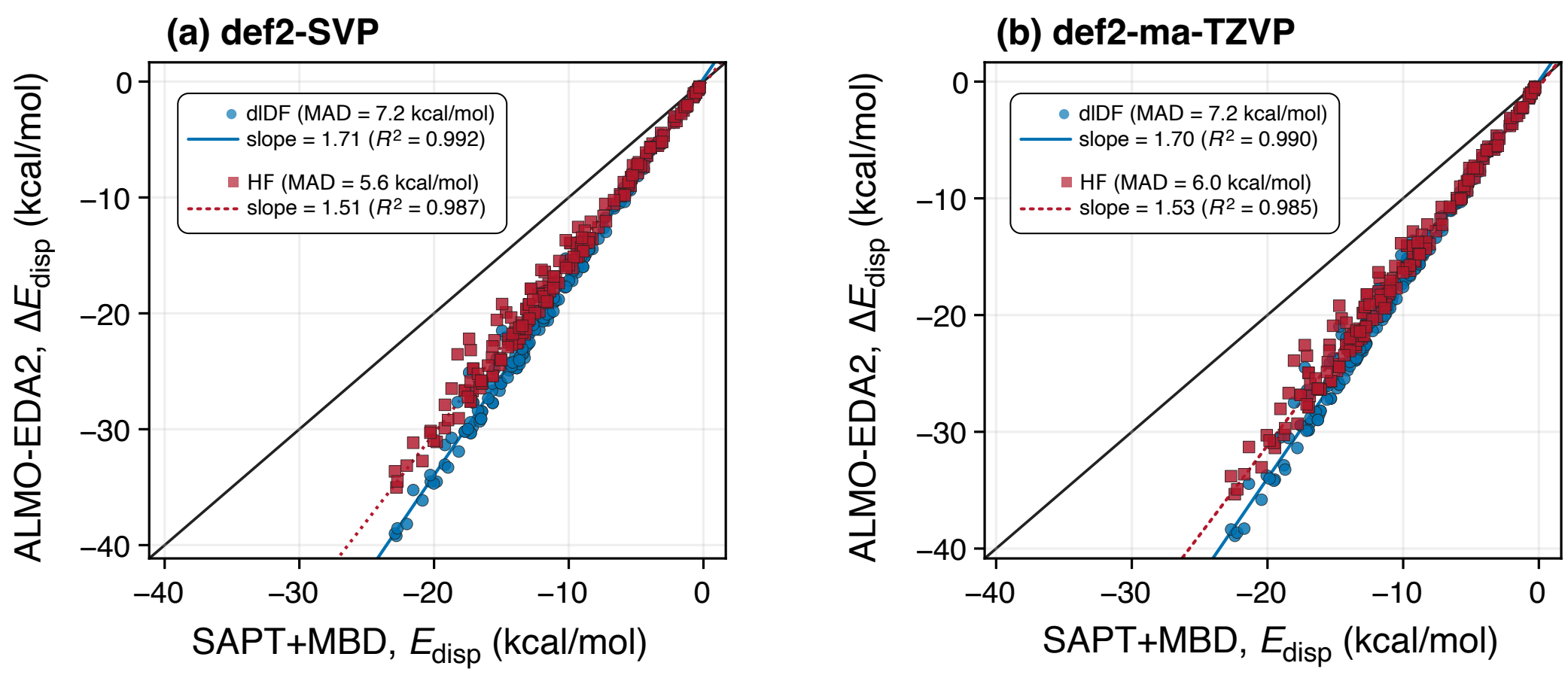

Fig. S12: Dispersion energies for the QUID dimers, correlating SAPT(LRC-ωPBE) + MBD with ALMO-EDA2(ωB97M-V) dispersion for (a) def2-SVP and (b) def2-ma-TZVP. ALMO-EDA2 dispersion energies use either DFXC = HF or DFXC = dlDF, as indicated, and linear fits to either data set are shown. Panel (b) is the same as Fig. 6.

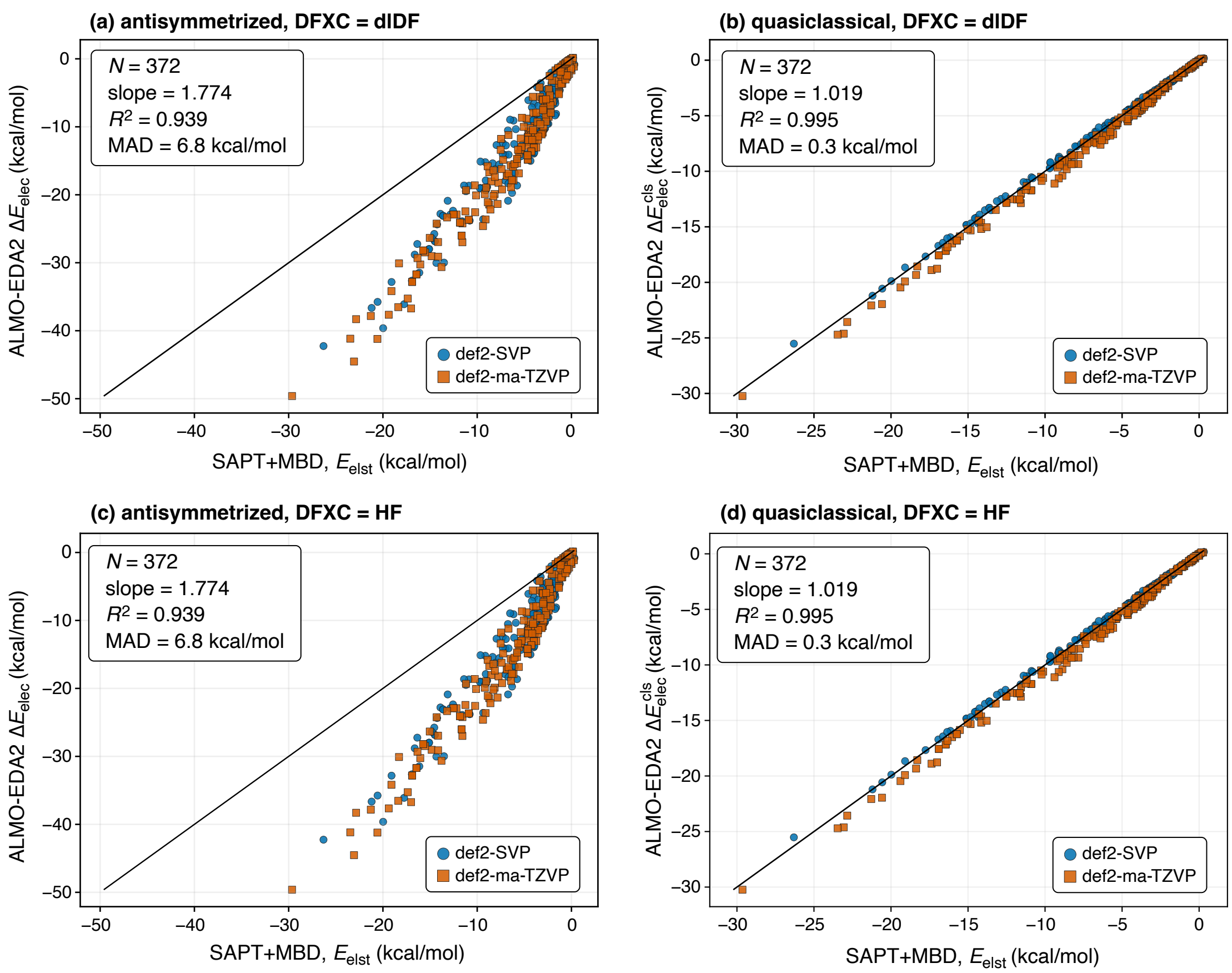

Fig. S13: Electrostatic interactions in the QUID data set, comparing results for two different basis sets. The four panels compare electrostatic energies from SAPT(LRC-ωPBE) + MBD and ALMO-EDA2(ωB97M-V) calculations, using (a) an antisymmetrized reference state with DFXC = dlDF, (b) a quasiclassical reference state with DFXC = dlDF, (c) an antisymmetrized reference state with DFXC = HF, and (d) a quasiclassical reference state with DFXC = HF.

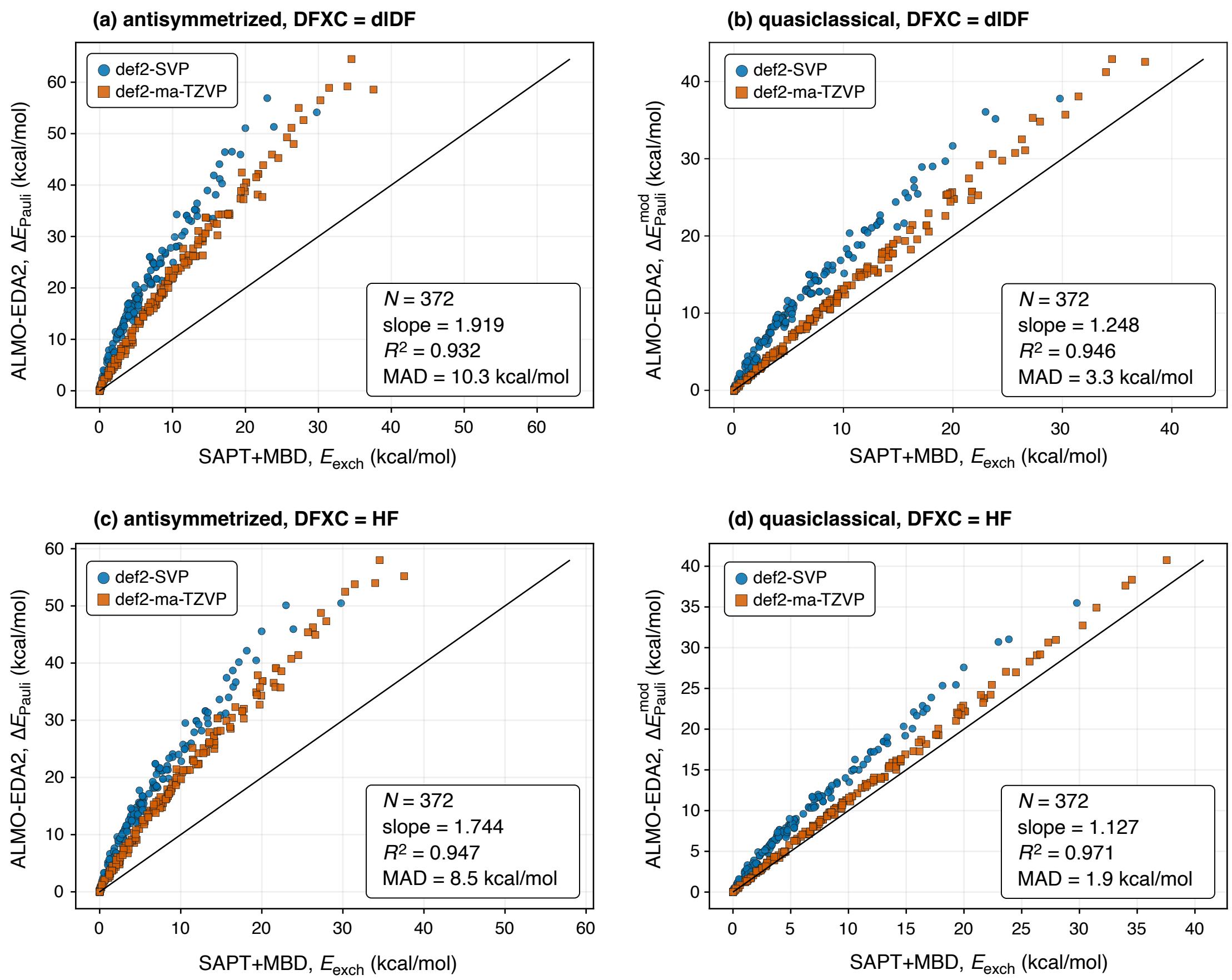

Fig. S14: Pauli repulsion interactions in the QUID data set, comparing results for two different basis sets. The four panels compare E_{exch} from SAPT(LRC-ωPBE) + MBD to Pauli repulsion from ALMO-EDA2(ωB97M-V) using (a) an antisymmetrized reference state with DFXC = dlDF, (b) a quasiclassical reference state with DFXC = dlDF, (c) an antisymmetrized reference state with DFXC = HF, and (d) a quasiclassical reference state with DFXC = HF.

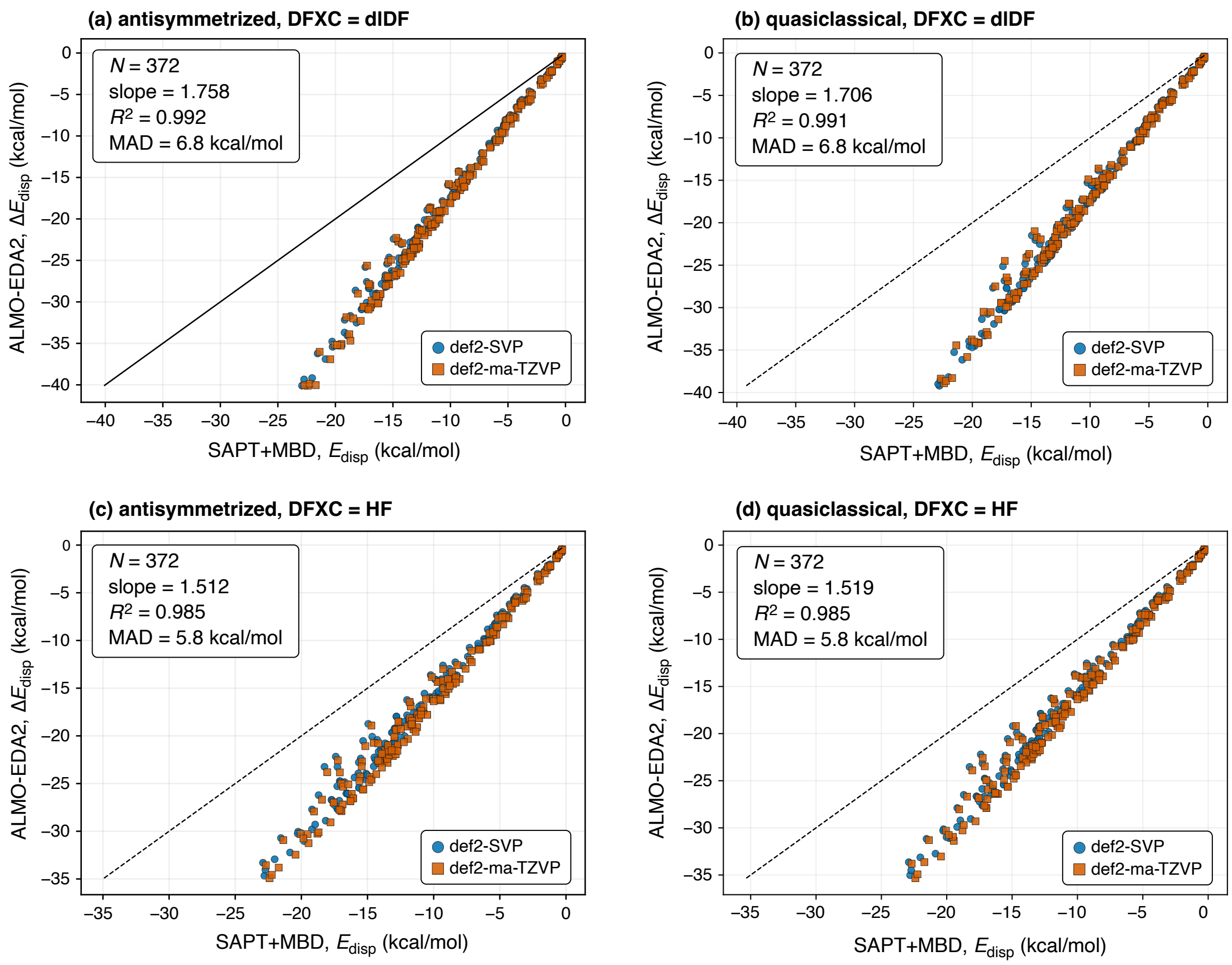

Fig. S15: Dispersion interactions in the QUID data set, comparing results for two different basis sets. The four panels compare E_{disp} from SAPT(LRC-ωPBE) + MBD to ΔE_{disp} from ALMO-EDA2(ωB97M-V), using (a) an antisymmetrized reference state with DFXC = dlDF, (b) a quasiclassical reference state with DFXC = dlDF, (c) an antisymmetrized reference state with DFXC = HF, and (d) a quasiclassical reference state with DFXC = HF.

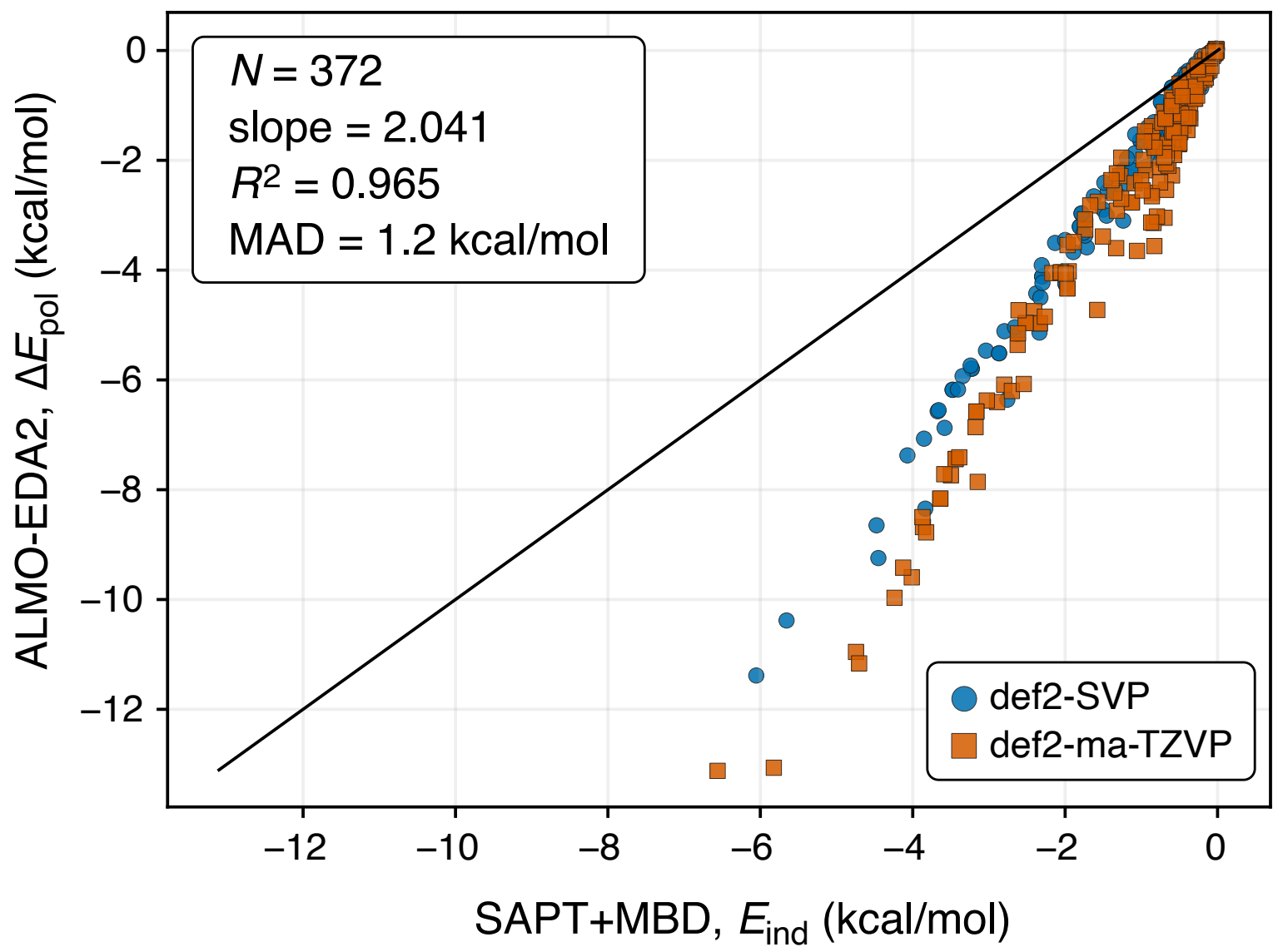

Fig. S16: Comparison of the induction energy from SAPT(LRC-ωPBE) + MBD with the polarization energy from ALMO-EDA2(ωB97M-V), for the QUID data set. Unlike the other energy components in Figs. S13–S15, ΔE_{pol} does not depend on either the reference state (antisymmetrized or quasiclassical) or the choice of dispersion-free functional.

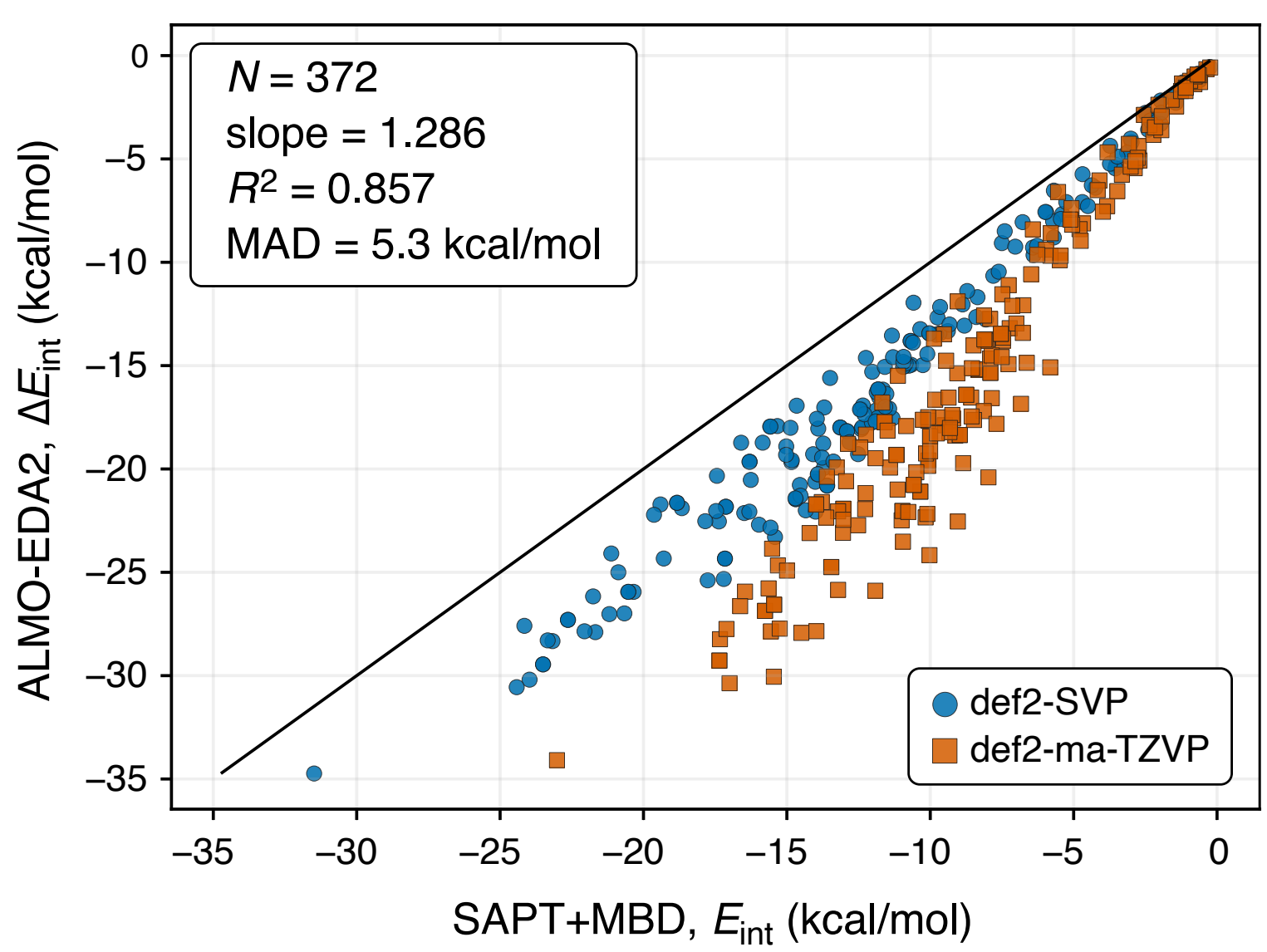

Fig. S17: Total interaction energies in the QUID data set, comparing SAPT(LRC-ωPBE) + MBD and ωB97M-V results in two different basis sets.

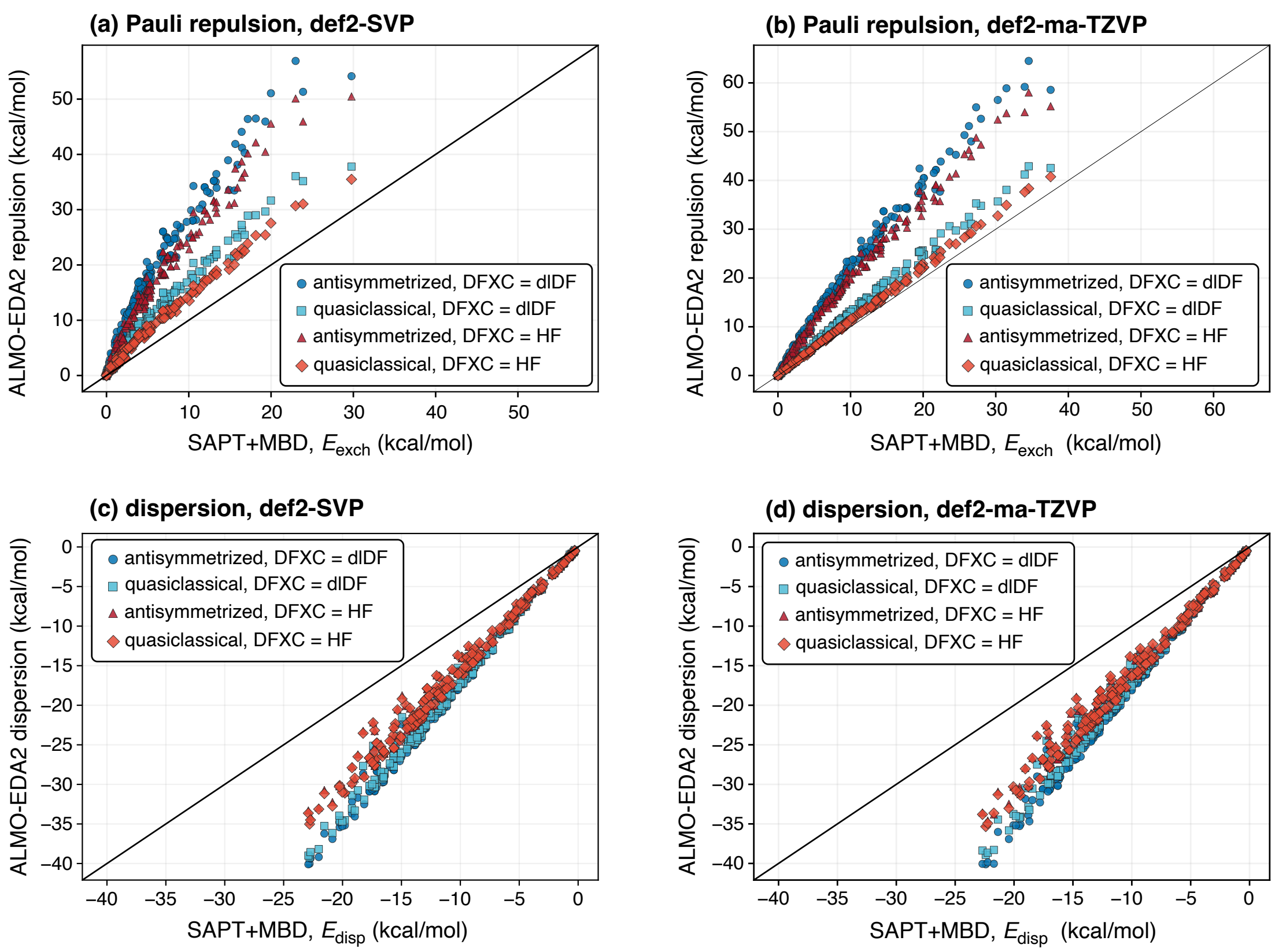

Fig. S18: Comparison of Pauli repulsion and dispersion energies for SAPT(LRC-ωPBE) + MBD versus ALMO-EDA(ωB97M-V) for the QUID data set, using different reference states (antisymmetrized or quasiclassical) and two different dispersion-free functionals (DFXC = dlDF or HF) for the ALMO-EDA2 calculations, as indicated. These panels contain the same data as in Figs. S14 and S15 but presented in a different way.

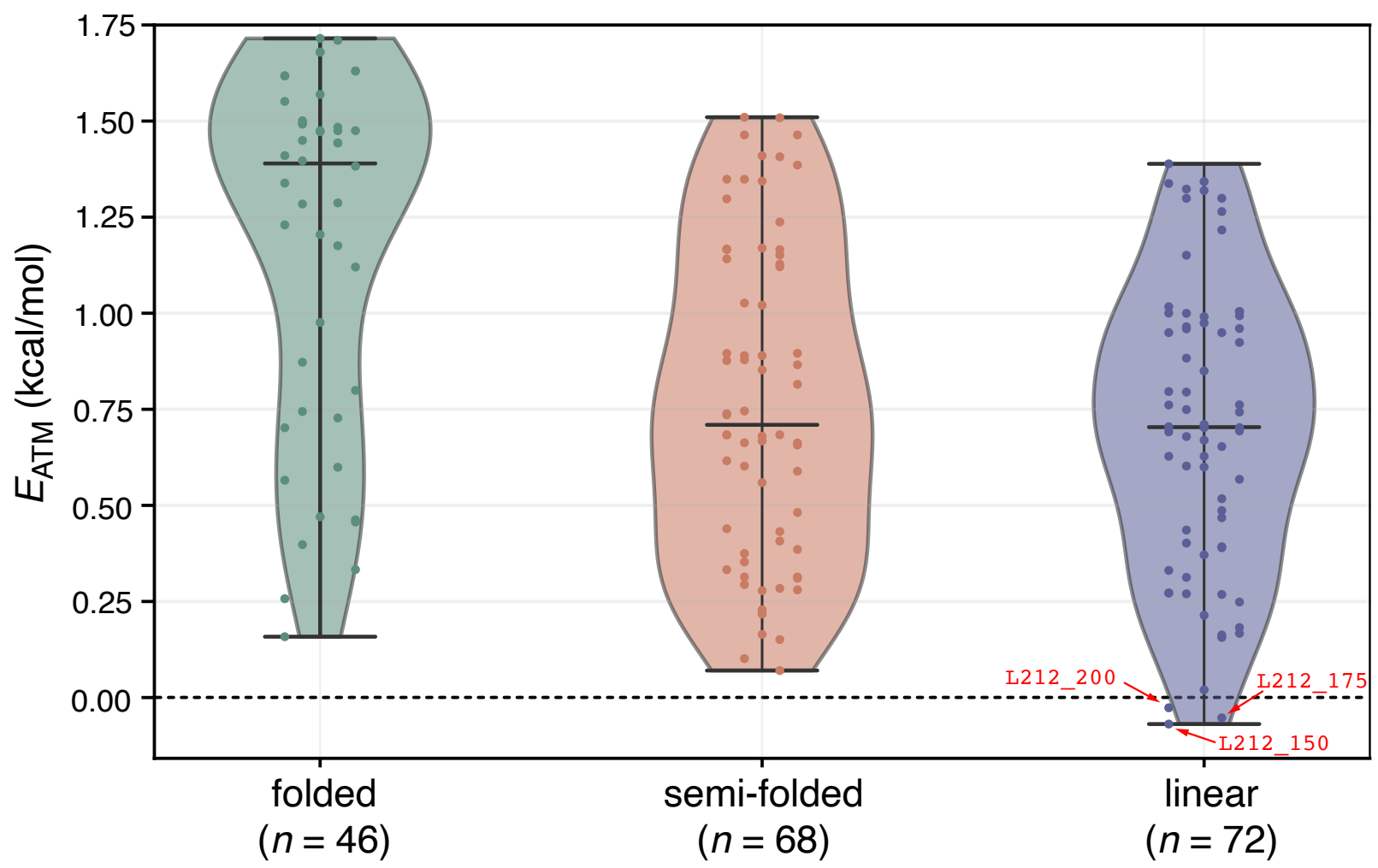

Fig. S19: Three-body ATM dispersion corrections for the QUID data set, sorted by topology categories. The middle bars are averages within each category and whiskers delineate minimum and maximum values. The ATM corrections are mostly positive (repulsive); three exceptions at large separation are indicated in red.

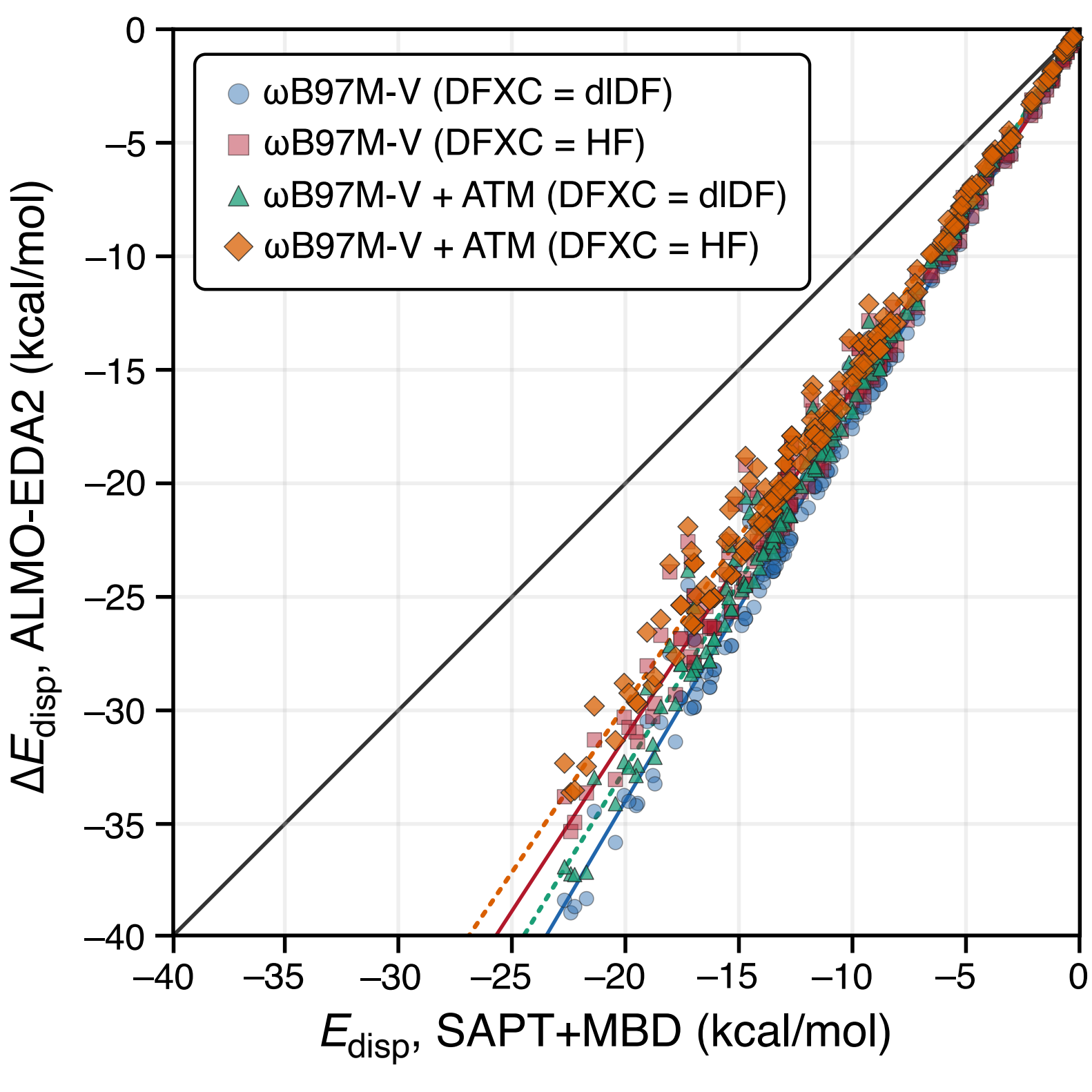

Fig. S20: Correlation between SAPT(LRC-ωPBE) + MBD dispersion and quasiclassical ALMO-EDA2(ωB97M-V) dispersion, comparing the latter both with and without three-body ATM.

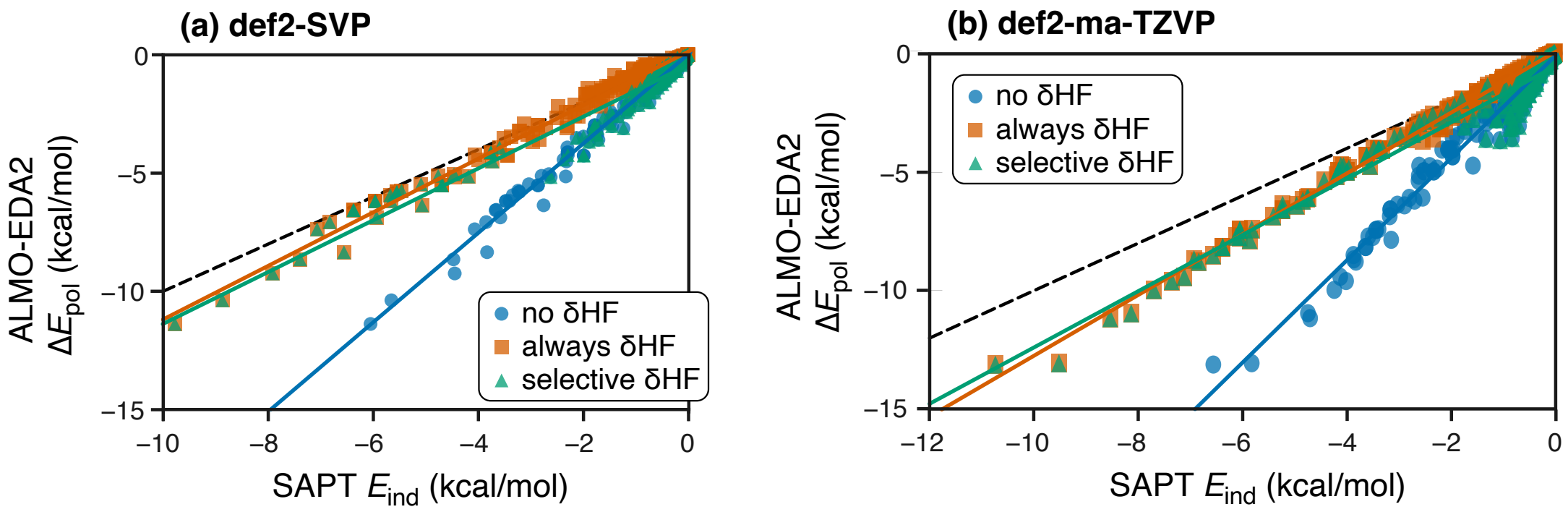

Fig. S21: ALMO-EDA2(ωB97M-V) polarization energies versus SAPT(LRC-ωPBE) + MBD induction energies in either of two different basis sets. Results are shown for SAPT induction computed either with or without the δ_{HF} correction, as in Fig. 13, and also for a procedure that adds δ_{HF} only when $\phi > 0.125$ [see Eq. (23)]. The dashed line indicates $y = x$.

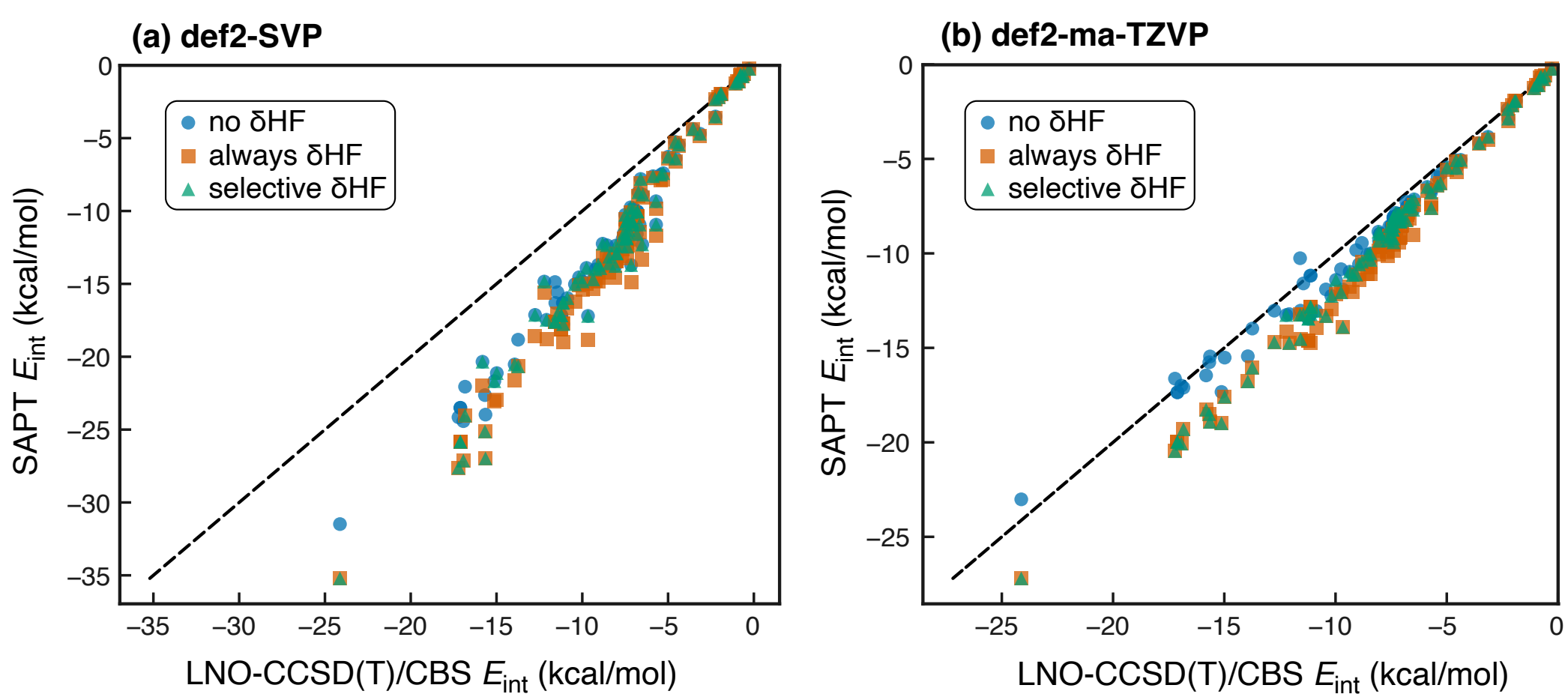

Fig. S22: SAPT(LRC-ωPBE) + MBD interaction energies in two different basis sets, as compared to LNO-CCSD(T)/CBS benchmarks. Results are shown for SAPT induction computed either with or without the δ_{HF} correction, as in Fig. 14, and also for a procedure that adds δ_{HF} only when $\phi > 0.125$ [see Eq. (23)]. The dashed line indicates $y = x$.

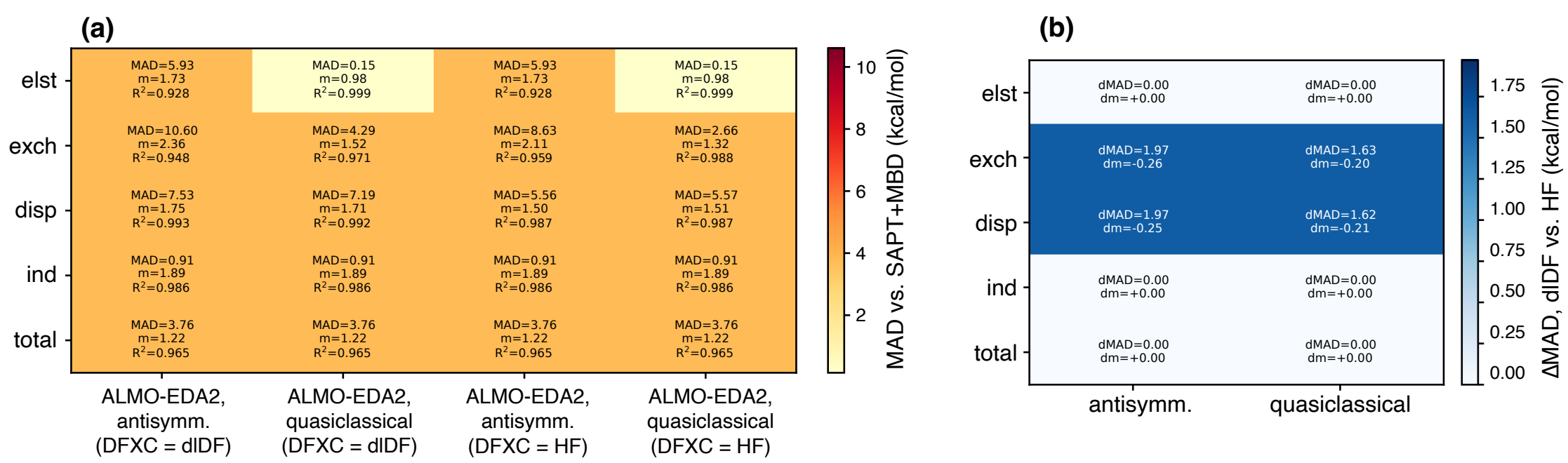

Fig. S23: Transferability map for the QUID dimers. (a) Statistical comparison of SAPT(LRC-ωPBE) + MBD to ALMO-EDA2(ωB97M-V), using both the original (antisymmetrized electrostatics) and quasiclassical electrostatics variants of the latter, with two different DFXC functionals. The statistical summary includes MADs, linear regression slopes, and correlation coefficients R^2. (b) Change in the MADs and slopes when HF replaces dlDF as the DFXC functional in the ALMO-EDA2 calculations. All calculations use the def2-ma-TZVP basis set. For a def2-SVP transferability map, see The corresponding def2-ma-TZVP version of these data appears in Fig. 15.

Table S1: Statistical differences between ALMO-EDA2 and SAPT2+(3) dispersion energies for the S22 dimers, including linear fit parameters.[a]

Target Functional	DFXC = dlDF MAD[b] (kcal/mol)	MAPD[c] (%)	slope	intercept (kcal/mol)	R^2	DFXC = HF MAD[b] (kcal/mol)	MAPD[c] (%)	slope	intercept (kcal/mol)	R^2
M06-2X	0.64	9.4	0.93	+0.16	0.971	1.66	23.1	0.75	−0.00	0.945
ωB97M-V	0.58	7.7	0.99	+0.22	0.964	1.26	17.4	0.81	+0.04	0.931
ωB97X-D	0.66	10.4	0.97	+0.07	0.959	1.31	17.4	0.79	−0.07	0.912
PBE0+D3(BJ)	0.47	7.0	0.91	−0.30	0.988	1.26	15.6	0.74	−0.44	0.959
B3LYP+D3(BJ)	0.48	6.9	1.01	+0.00	0.982	0.94	12.3	0.84	−0.16	0.954
BLYP+D3(BJ)	0.67	10.3	1.00	+0.25	0.962	1.16	17.4	0.84	+0.08	0.919
MBD[d]	0.71	13.8	1.03	−0.47	0.985					

[a]SAPT2+(3)/aug-cc-pVTZ (from Ref. 2) vs. ALMO-EDA/def2-QZVPD. This table augments Table 1 with linear fit parameters. [b]Mean absolute deviation. [c]Mean absolute percentage deviation. [d]SAPT(LRC-ωPBE) + MBD/def2-QZVPD, which does not require a DFXC functional but is included here for comparison to the ALMO-EDA2 values.

Table S2: Statistical differences between ALMO-EDA2 and SAPT2+(3) dispersion energies for the S22 dimers, calculated with distorted monomer densities.[a]

Target Functional	DFXC = dlDF MAD[b] (kcal/mol)	MAPD[c] (%)	DFXC = HF MAD[b] (kcal/mol)	MAPD[c] (%)
ωB97M-V	0.51	8	1.11	14
ωB97X-D	0.68	11	1.88	25
PBE0+D3(BJ)	0.39	6	1.61	19
B3LYP+D3(BJ)	0.29	5	1.18	15
BLYP+D3(BJ)	0.29	5	0.39	6

[a]SAPT2+(3)/aug-cc-pVTZ (Ref. 2) versus ALMO-EDA2/def2-QZVPD, with the latter computed using $\tilde{\rho}_M(\mathbf{r})$; see Section 2.3.2. [b]Mean absolute deviation. [c]Mean absolute percentage deviation.

Table S3: Monomer-specific range-separation parameters employed in SAPT(LRC-ωPBE) + MBD calculations on the S66 data set, obtained using GDD tuning with the def2-QZVPD basis set.

Complex	ω_{GDD} (bohr^{-1})		Complex	ω_{GDD} (bohr^{-1})		Complex	ω_{GDD} (bohr^{-1})	
	mono. 1	mono. 2		mono. 1	mono. 2		mono. 1	mono. 2
01	0.505	0.503	23	0.378	0.342	45	0.435	0.324
02	0.505	0.430	24	0.344	0.344	46	0.351	0.323
03	0.504	0.412	25	0.352	0.351	47	0.338	0.340
04	0.505	0.351	26	0.343	0.343	48	0.350	0.353
05	0.429	0.430	27	0.339	0.353	49	0.338	0.349
06	0.428	0.412	28	0.345	0.343	50	0.342	0.437
07	0.429	0.352	29	0.354	0.343	51	0.435	0.437
08	0.429	0.503	30	0.341	0.425	52	0.337	0.391
09	0.411	0.427	31	0.342	0.423	53	0.339	0.379
10	0.413	0.411	32	0.342	0.438	54	0.341	0.502
11	0.413	0.352	33	0.348	0.418	55	0.343	0.429
12	0.412	0.505	34	0.323	0.323	56	0.343	0.411
13	0.352	0.429	35	0.323	0.328	57	0.344	0.351
14	0.351	0.410	36	0.329	0.329	58	0.355	0.355
15	0.350	0.350	37	0.338	0.327	59	0.436	0.504
16	0.351	0.507	38	0.337	0.338	60	0.437	0.391
17	0.341	0.342	39	0.341	0.336	61	0.322	0.394
18	0.502	0.354	40	0.342	0.328	62	0.323	0.377
19	0.428	0.355	41	0.341	0.322	63	0.340	0.391
20	0.391	0.391	42	0.343	0.338	64	0.350	0.424
21	0.377	0.377	43	0.343	0.327	65	0.353	0.437
22	0.392	0.342	44	0.424	0.324	66	0.412	0.349

Table S4: Monomer-specific range-separation parameters employed in SAPT(LRC-ωPBE) + MBD calculations on the L7 data set, obtained using GDD tuning with the def2-TZVPD basis set.

Complex	ω_{GDD} (bohr^{-1})	
	monomer 1	monomer 2
C2C2PD	0.273	0.273
C3A	0.321	0.248
C3GC	0.295	0.248
CBH	0.260	0.260
GCGC	0.296	0.296
GGG	0.321	0.297
PHE	0.295	0.272

Table S5: Range separation parameters for the eleven QUID reference monomers comparing fixed values used in the original calculations with tuned values from the GDD procedure in two basis sets.

Monomer	Structure	Fragment[a]	ω (bohr^{-1})		
			fixed[b]	GDD-tuned	
				def2-SVP	def2-TZVP
F1	F1B1	1	0.505	0.279	0.267
F2	F2B1	1	0.505	0.284	0.271
L1	L1B1	1	0.505	0.282	0.270
L2	L2B1	1	0.505	0.281	0.268
L3	L3B1	1	0.505	0.276	0.264
L4	L4B1	1	0.505	0.280	0.269
SF1	SF1B1	1	0.505	0.291	0.277
SF2	SF2B1	1	0.505	0.284	0.270
SF3	SF3B1	1	0.505	0.267	0.258
Benzene	F1B1	2	0.503	0.347	0.341
Imidazole	F1I1	2	0.503	0.387	0.368

[a]Fragment indices follow the monomer partition of the reference configuration; ligand monomers (benzene and imidazole) carry fragment index 2 and protein monomers carry fragment index 1. [b]Used for the SAPT(LRC-ωPBE) + MBD calculations on the QUID dimers that are reported in the main text.

Table S6: Range-separation parameters ω and signed interaction energy differences for the QUID complexes in two different basis sets, comparing fixed versus GDD-tuned values of ω.

Monomer	n^a	ω (bohr^{-1})		differences (kcal/mol)^b		
		fixed^c	GDD	average	minimum	maximum
**def2-SVP basis set**						
F1	6	0.505	0.279	0.71	0.40	1.01
F2	40	0.505	0.284	0.49	0.01	0.96
L1	4	0.505	0.282	0.51	0.36	0.69
L2	60	0.505	0.281	0.28	−0.02	0.92
L3	6	0.505	0.276	0.37	0.14	0.76
L4	2	0.505	0.280	0.55	0.33	0.76
SF1	3	0.505	0.291	1.05	0.56	1.77
SF2	60	0.505	0.284	0.40	0.01	1.56
SF3	5	0.505	0.267	0.70	0.30	1.24
Benzene	92	0.503	0.347	0.31	−0.02	0.83
Imidazole	94	0.503	0.387	0.51	−0.02	1.77
**def2-TZVP basis set**						
F1	6	0.505	0.267	1.12	0.77	1.60
F2	40	0.505	0.271	0.87	0.10	1.76
L1	4	0.505	0.270	0.88	0.74	1.04
L2	60	0.505	0.268	0.53	−0.02	1.47
L3	6	0.505	0.264	0.76	0.48	1.14
L4	2	0.505	0.269	0.93	0.68	1.17
SF1	3	0.505	0.277	1.47	0.79	2.35
SF2	60	0.505	0.270	0.69	0.01	2.10
SF3	5	0.505	0.258	1.136	0.62	1.57
Benzene	92	0.503	0.341	0.62	−0.01	1.58
Imidazole	94	0.503	0.368	0.82	−0.02	2.35

[a]Number of dimers contributing to the average in each row. Each dimer contributes once to a protein-monomer group and once to a ligand-monomer group, so the reported row counts are not additive. [b]Statistical summary of $\Delta E = E_{\rm int}(\omega_{\rm GDD}) - E_{\rm int}(\omega_{\rm fixed})$, including all n dimer interaction energies that contain the monomer as protein or a ligand. [c]Used for the SAPT(LRC-ωPBE) + MBD calculations on the QUID dimers that are reported in the main text.
